# DFT Insights on Ligand Photodissociation Pathways in Ruthenium–Terpyridine Complexes: ^3^MLCT- or ^3^MC-Triggered?

**DOI:** 10.1021/acs.inorgchem.6c00971

**Published:** 2026-04-22

**Authors:** Stefano Scoditti, Gloria Mazzone, Emilia Sicilia, Luca Salassa

**Affiliations:** a 226245 Donostia International Physics Center, Paseo Manuel de Lardizabal 4, Donostia 20018, Spain; b Polimero eta Material Aurreratuak: Fisika, Kimika eta Teknologia, Kimika Fakultatea, Euskal Herriko Unibertsitatea UPV/EHU, Donostia-San Sebastian 20018, Spain; c Department of Chemistry and Chemical Technologies, 428781 Università della Calabria, Arcavacata di Rende (CS) 87036, Italy; d Ikerbasque, Basque Foundation for Science, Bilbao 48011, Spain

## Abstract

We computationally elucidated the mechanism of acetonitrile (ACN) photodissociation in Ru-terpyridine PACT complexes from Turro’s series, [Ru­(tpy)­(L)­(ACN)]^*n*+^, where tpy = 2,2′:6′,2″-terpyridine and L = 4,4′-dimethyl-2,2′-dipyridylbipyridine (**1**), acetylacetonate (**2**), or 1,3-diphenylpropane-1,3-dione (**3**). DFT reproduced the experimental ^3^MLCT–^3^MC energy-gap trends and revealed an atypical dissociative ^3^MC minimum dominated by tpy distortion rather than pronounced Ru–ACN elongation. From this distorted ^3^MC state, we located a transition structure for ACN loss leading to a pentacoordinate intermediate (^3^RuP), demonstrating that photosubstitution can proceed without a canonical elongated-bond ^3^MC geometry. For **1**, the ^3^MC → ^3^RuP dissociation barrier exceeded the ^3^MLCT → ^3^MC internal-conversion barrier, whereas for **2**, the two processes were competitive, rationalizing experimental photodissociation quantum yields. Relaxed scans suggest that both ^3^MC- and ^3^MLCT-mediated pathways for ACN loss are plausible and competitive across the series. The strongest support for ^3^MLCT-mediated dissociation is found for complex **1**, where a transition state has been localized. Finally, sterically tuned tpy derivatives bearing ortho- or meta-methyl substituents (**4**, **5**) biased ^3^MLCT–^3^MC conversion by stabilizing the distorted ^3^MC state while leaving the ACN-loss barrier largely unchanged, providing a design strategy to tune photosubstitution.

## Graphical abstract

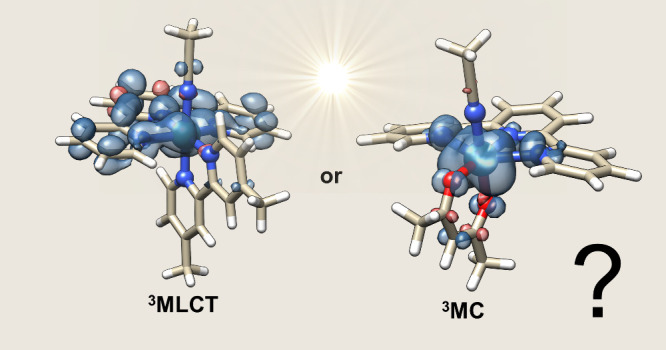

## Introduction

The photochemistry of Ru­(II) polypyridyl complexes has been extensively studied over the past several decades. Initial investigations were primarily driven by fundamental interest, as well as the potential of this class of compounds in solar energy conversion applications.^1^ More recently, Ru polypyridyl complexes have demonstrated remarkable versatility, finding roles in a wide range of technological domains, including photoredox catalysis^2^ and photoresponsive polymers.^3^ In the biomedical field, Ru­(II) diamine derivatives initially attracted attention as long-lived luminescent probes for imaging and sensing.^4,5^ Over time, their application space has broadened significantly. These complexes have emerged as effective caging scaffolds for the light-triggered release of neurotransmitters^6^ and as photocatalysts for proximity-based protein labeling.^7^ Another critical medical use of their photoactivity is in phototherapy, where Ru-based compounds are being developed for both photodynamic therapy (PDT)^8^ and photoactivated chemotherapy (PACT),^9^ which are promising strategies for spatiotemporally controlled therapeutic interventions, including in clinical settings.^10^

The interplay between excited states is a critical factor underlying all uses of Ru polypyridyl complexes, as it governs their photophysical behavior and photochemical reactivity. This remains a subject of intense study, with time-resolved optical and X-ray techniques, supported by computational modeling, providing key insights into their kinetic and structural evolution.^11–15^

In PDT and PACT, specifically, the relative energies of the ^3^MLCT, ^3^LC/^3^LLCT, and ^3^MC states determine whether the complexes form long-lived excited states capable of promoting singlet oxygen sensitization or electron transfer processes with cellular components (PDT) or undergo intramolecular transformations, such as ligand release, to generate reactive Ru–OH_2_ species (PACT) and/or to uncage bioactive pharmacophores. The most widely accepted mechanism of photosubstitution in polypyridyl Ru­(II) compounds is illustrated in Figure 1. Upon irradiation, a ^1^MLCT excited state is formed, followed by an ultrafast intersystem crossing (ISC) process, which does not require an energy barrier, to populate a ^3^MLCT excited state. Subsequently, the ^3^MLCT state undergoes thermally activated conversion to a ^3^MC, from which ligand dissociation forms a penta-coordinated complex. The reactivity and stability of this five-coordinate species depend strongly on the ligands at the metal center. Ru polypyridyl complexes often restore six-coordinate geometry by binding a solvent molecule (e.g., water) via either singlet or triplet pathways.^16^ Unlike the barrierless ^1^MLCT–^3^MLCT transition, the ^3^MLCT–^3^MC conversion requires overcoming an activation energy barrier (ΔG^‡^). In addition, ^3^MLCT–^3^MC energy gap (ΔG_r_) is strongly system-dependent, and in some cases, the ^3^MC state may lie lower in energy than the ^3^MLCT state.^17,18^

**Figure 1:**
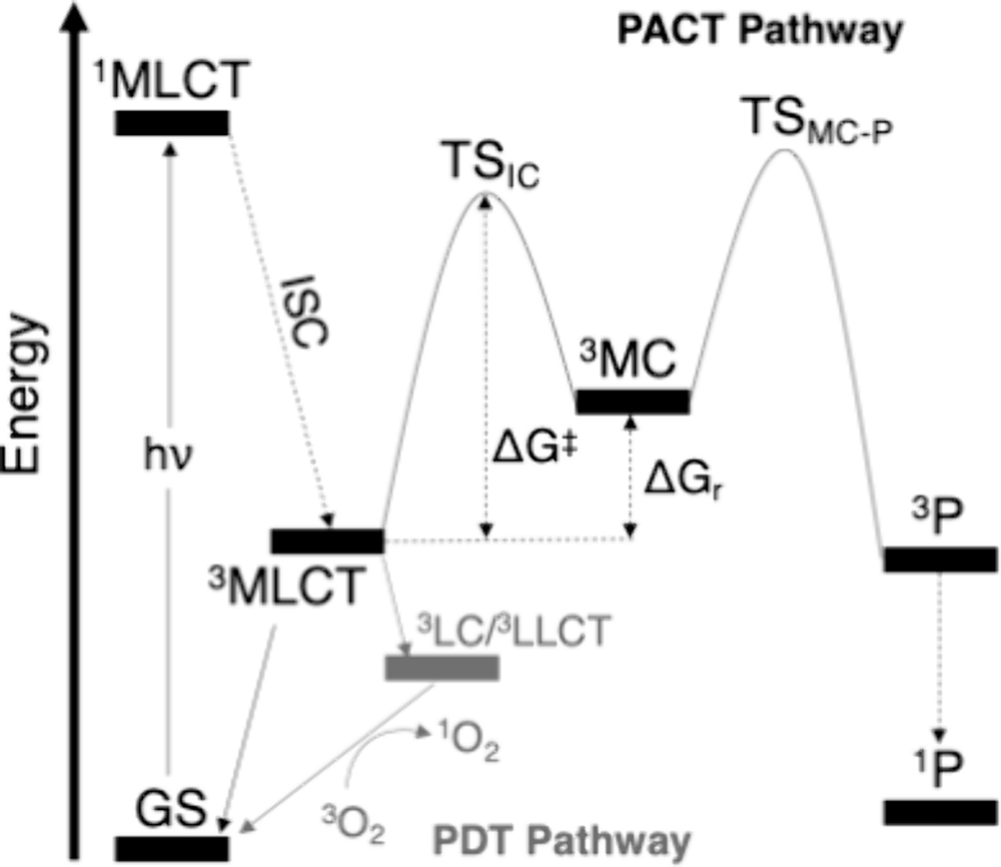
Schematic representation of excited-state interplay in polypyridyl Ru­(II) complexes involved in PDT (gray) and PACT (black). Acronyms: ISC = Intersystem Crossing; GS = Ground State; MLCT = Metal-to-Ligand Charge Transfer; LC = Ligand-Centered; LLCT = Ligand-to-Ligand Charge Transfer; MC = Metal-Centered; *P* = Photodissociation product.

Recent work by Turro and Bonnet has challenged this traditional view of the ligand photodissociation mechanism.^19–22^ Their findings suggest that photosubstitution of acetonitrile or thioether ligands in terpyridine Ru complexes can occur without involving ^3^MC states. Notably, Turro and colleagues demonstrated that complexes of the type [Ru­(tpy)­(L)­(ACN)]^n+^ (tpy = 2,2’:6’,2’’-terpyridine, L = bidentate ligand, and ACN = CH_3_CN) undergo photoinduced ACN dissociation with quantum yields that increase as the ^3^MLCT–^3^MC energy gap widens (Figure 2).^21^ This counterintuitive trend implies that photosubstitution may proceed directly from the ^3^MLCT state, bypassing the thermally activated ^3^MC intermediate, which would otherwise serve only as a nonreactive deactivation pathway. Such results introduce a new paradigm in the photochemistry of metal complexes and applications, such as PACT, renewing interest in the mechanisms underlying ligand photosubstitution in Ru polypyridyl systems.

**Figure 2:**
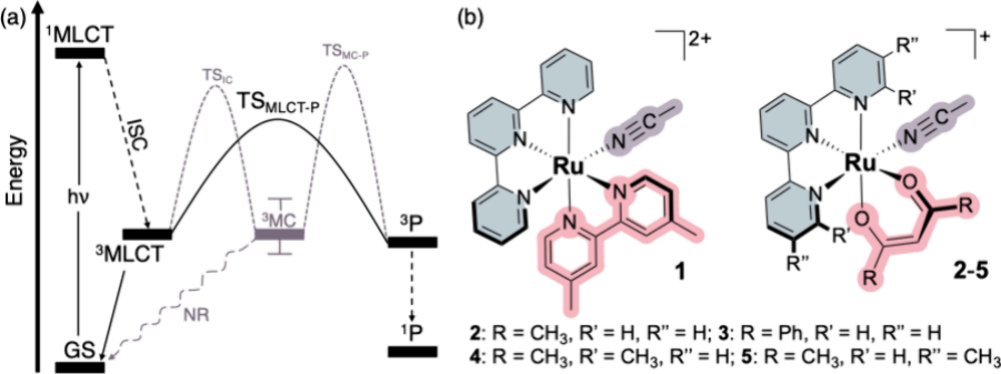
(a) Proposed ^3^MLCT-mediated pathway for the photodissociation of Ru–tpy complexes, as described by Turro and co-workers; (b) schematic representation of the Ru–tpy complexes investigated in this study. Acronyms: NR = nonradiative decay.

Turro and co-workers supported their proposed mechanism by integrating photophysical measurements with Density Functional Theory (DFT) calculations, including characterization of the spin densities of ^3^MLCT and ^3^MC states and orbital analyses across several complexes (*vide infra*). However, a complete computational exploration of the full mechanism was not undertaken for this class of compounds.

To address this issue, we carried out a detailed DFT study on selected Ru­(II) complexes reported in the literature. Specifically, we investigated the photodissociation pathway of three Ru complexes (**1**–**3**) from Turro’s series, [Ru­(tpy)­(L)­(ACN)]^n+^, where the bidentate ligand L is 4,4’-dimethyl-2,2’-dipyridylbipyridine (**1**), acetylacetonate (**2**) or 1,3-diphenylpropane-1,3-dione (**3**). In addition, two newly designed derivatives (**4** and **5**), based on the structure of complex **2**, were considered. In these complexes, methyl groups were introduced at the ortho or meta positions of the terminal pyridyl rings of the tpy ligand. This modification aims to tune the ^3^MLCT–^3^MC energy gap and assess its impact on reactivity, in analogy with previous findings on [Ru­(bpy)_2_(phen)]^2+^ derivatives reported by Takashima, where ortho-methyl substitution was shown to strongly influence the IC mechanism by promoting structural distortion.^23^

Our results indicate that the ^3^MLCT–^3^MC internal conversion is not necessarily rate-determining (as often assumed), and that photodissociation can proceed through multiple, complex pathways. Transition-state localization indicates that, in some complexes, ACN can dissociate directly from the ^3^MLCT state, consistent with Turro’s hypothesis. However, monodentate ligand release can also proceed via a more complex pathway in which ^3^MC states are central. Notably, the key ^3^MC state is atypical: rather than being defined by a marked Ru–ACN bond elongation, dissociation is driven by distortion from planarity of the tridentate tpy ligand.

We hope this work contributes to the ongoing debate by providing a comprehensive computational perspective on the excited-state reactivity of Ru polypyridyl complexes. A full account of the computational results for this study is provided in the Supporting Information (Figures S1–S9, Tables S1–S6).

## Results and Discussion

### Early Computational Evidence of a ^3^MLCT-Driven Photodissociation

Several studies by Turro and co-workers provided valuable insights into the ACN photodissociation mechanism through DFT calculations.^19–21^ Their findings revealed a linear correlation between the quantum yield (Φ) for ACN ligand exchange in water and two key parameters: the Mulliken spin density (SD) on the Ru center in the ^3^MLCT state and the percentage of Ru d-character in the ground-state highest-occupied molecular orbital (HOMO). The lowest SD values and highest Φ values were observed in complexes containing strongly electron-donating bidentate ligands, such as acetylacetonate (acac) and acac-type ligands. These ligands enhance orbital mixing between Ru and the coordinated acac, increasing ligand character in the excited state while reducing spin localization on the metal. According to Turro’s analysis, a lower Ru-centered SD weakens the Ru–ACN bond by diminishing Ru π back-donation, thereby facilitating photodissociation. While these correlations align well within ligand series (e.g., bpy- or acac-based complexes), they do not fully capture the experimental order of Φ values and rely on subtle variations in the predictive descriptors, which may be sensitive to the computational methods used. Moreover, although a slightly higher spin density in the ^3^MLCT state seemed to correlate with a lower photosubstitution quantum yield, the actual differences were very small, and given the known basis-set dependence of Mulliken populations, this trend should be viewed with caution.^24^

To initiate our study, we examined the ^3^MLCT and ^3^MC excited-state geometries of two model parent complexes previously investigated by Turro, namely **1** and **2** (Figure 2). These compounds present distinct ligand environments: **1** contains a 4,4’-dimethyl-2,2’-bipyridine ligand, while **2** features an acac ligand. They were selected as representative models of distinct ^3^MLCT–^3^MC internal conversion (IC) barriers and photosubstitution efficiency. Specifically, **1** exhibits a low IC energy barrier (585 ± 35 cm^–1^) and a low quantum yield at 450 nm (Φ = 0.0031). In contrast, **2** presents a significantly higher IC energy barrier (1222 ± 287 cm^–1^), corresponding to a markedly increased quantum yield (Φ = 0.014).^21^

Based on previously reported ^3^MLCT structures and Φ_450_ values for **1** and **2**,^20^ one would expect a longer Ru–N­(ACN) bond in **2** than in **1**, reflecting a weaker metal–ligand interaction in **2**. However, the experimental trend is the opposite: the Ru–N bond distance is 2.032 Å in **1** and 2.004 Å in **2**. The ^3^MLCT states of both complexes using our selected computational protocol (B3LYP-D3/SDD/6–31G** vs PBE/SDD/TZVP)^20^ show SDs of 0.926 au for **1** and 0.864 au for **2**, consistent with the expected literature trend of increased ligand character in **2**. Moreover, the computed Ru–N bond lengths remained inverted relative to expectations: 2.055 Å for **1** and 2.045 Å for **2**. This apparent inconsistency suggests that Ru–ACN bond lengths and SD values may not be reliable predictors for the bonding dynamics involved in photodissociation.

For this reason, we focused on the change in the C–N bond length of the ACN ligand and its corresponding vibrational stretching frequency as an additional informative parameter to assess Ru π-backbonding and its correlation with photodissociation. For comparison, a free ACN molecule optimized using the same computational protocol shows a C–N bond length of 1.161 Å and a vibrational frequency of 2366 cm^–1^. In both **1** and **2**, coordination to Ru­(II) leads to a slight C–N bond shortening (<0.05 Å) and a modest increase in vibrational frequency in both the ground and ^3^MLCT states (Table S1), indicating a strengthening of the C–N bond. This behavior contrasts with typical π-backbonding, which weakens the bond and results in a redshift of the stretching frequency. Similar blueshifts have been reported for ACN coordinated to metal centers such as Ag­(I).^25^ These findings suggest minimal π-backdonation to the acetonitrile ligand and no significant difference between **1** and **2** in this regard. Supporting this conclusion, NBO analysis of both ^3^MLCT states confirms the absence of appreciable Ru→ACN π-backbonding (*data not shown*).

Kraka and co-workers recently analyzed the bond strength order (BSO) of the Ru–ACN bond in Ru–tpy complexes reported by Turro, including compounds **1** and **2**.^26^ They found that complexes with the highest photosubstitution quantum yields in water possess the strongest Ru–ACN bond in the triplet state, challenging the notion that spin density or static bond strength alone dictates photodissociation. Instead, this behavior points to a more nuanced interplay of excited-state electronic factors, emphasizing the need for detailed mechanistic investigation. Computationally, the ^3^MC state remains poorly characterized in this series, with key transition states for ^3^MLCT-to-^3^MC conversion and ACN dissociation yet to be located. To address these gaps, compounds **1** and **2** were selected for targeted analysis of their photodissociation pathways.

### ^3^MLCT and ^3^MC States and Their Internal Conversion

The optimized ^3^MLCT and ^3^MC excited-state structures of **1** and **2**, together with selected geometrical parameters, are shown in Figure 3 and Table 1. SD surfaces indicate that in the ^3^MLCT state, the unpaired electron density is delocalized over the Ru center (0.926 and 0.864 au) and the tridentate tpy ligand. In contrast, in the ^3^MC state of both complexes, the SD is almost entirely localized on the Ru atom (1.775 and 1.762 au). The difference in SD on Ru between the two complexes is significantly smaller in the ^3^MC state than in the ^3^MLCT state, consistent with the different influence exerted by the bpy and acac ligands on the Ru-tpy fragment. For both **1** and **2**, the ^3^MLCT structures show only minor deviations from the GS, whereas the ^3^MC states display pronounced distortions in the Ru coordination sphere. In the ^3^MC states of both complexes, the Ru–N­(ACN) bond length increases only modestly relative to the ground state (no more than 0.05 Å), whereas the tpy ligand deviates strongly from planarity, with the Ru–N2 and Ru–N4 bonds elongated by nearly 0.3 Å. In addition, no partial dissociation or significant distortion of either the bpy or acac ligand is observed in these complexes, suggesting that their role in the photodissociation is primarily electronic rather than structural.

**Figure 3:**
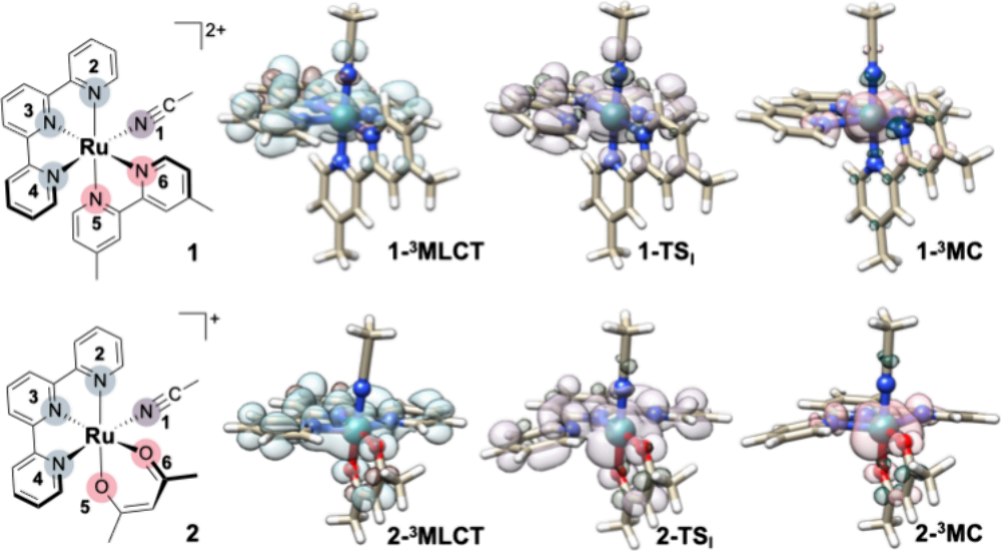
Atom numbering and optimized structures of the ^3^MLCT, ^3^MC triplet states, and the MLCT–MC internal conversion transition state (TS_I_) for **1** and **2**, obtained within the unrestricted DFT formalism.

**Table 1:** Selected Geometrical Parameters for the Ground State (GS), Triplet Excited States (^3^MLCT and ^3^MC), and the ^3^MLCT → ^3^MC Conversion Transition State (TS_I_) of Complexes **1** and **2** ^a^

	1				2			
	GS	^3^ **MLCT**	TS_I_	^3^ **MC**	GS	^3^ **MLCT**	TS_I_	^3^ **MC**
Ru–N1	2.039	2.055	2.045	2.055	2.015	2.045	2.086	2.065
Ru–N2	2.098	2.082	2.161	2.392	2.091	2.085	2.215	2.385
Ru–N3	1.993	2.022	2.083	2.256	1.966	2.009	2.091	2.188
Ru–N4	2.098	2.083	2.158	2.403	2.091	2.085	2.215	2.380
Ru–N5/O5	2.073	2.069	2.094	2.085	2.066	2.026	2.025	2.043
Ru–N6/O6	2.101	2.094	2.088	2.092	2.101	2.048	2.047	2.050
N1–Ru–N3	89.4	85.5	84.9	84.8	93.6	94.6	95.7	95.4
N2–Ru–N4	158.6	153.4	146.6	137.7	159.8	155.1	147.0	141.5
N3–Ru–N5/O5	96.6	97.7	99.3	99.1	87.3	86.4	86.2	86.6
N3–Ru–N6/O6	174.4	176.0	177.1	176.1	178.0	177.7	177.4	176.2
N2–C–C–N3	0.6	–1.7	–6.9	–11.1	–1.6	1.9	16.5	16.1
SD (Ru)		0.926	1.220	1.789		0.864	1.363	1.762
ΔG^‡^			1.2				3.6	
ΔG_r_				–4.4				0.3

a Bond distances are given in Å and angles in degrees. Activation (ΔG^‡^) and reaction (ΔG_r_) free energies for the ^3^MLCT → ^3^MC conversion in water are reported in kcal mol^–1^. The spin density (SD) on the Ru atom is provided for the triplet states and transition state connecting them.

Similar localized ^3^MC states were previously identified by Jakubikova in related tpy-based Ru complexes,^27^ such as [Ru­(tpy)_2_]^2+^, [Ru­(tpy)­(bpy)­(H_2_O)]^2+^, and [Ru­(tpy)­(bpy)­Cl]^+^, where the ^3^MC state was found to be the lowest-lying triplet. Thermodynamically, our results indicate that in **1** the ^3^MC state is more stable than the ^3^MLCT state by 4.4 kcal mol^–1^, whereas in **2** the two states are nearly thermoneutral, differing by only 0.3 kcal mol^–1^ in favor of the ^3^MLCT. The observed exergonicity for **1** contrasts with the view that the ^3^MLCT is the lowest triplet state for this complex.^20^

To examine these photophysical mechanisms in more detail, we analyzed the IC pathway for both complexes. The corresponding energetic data are summarized in Table 1, together with schematic representations of the identified transition states (TSs, Figure 3), while fully optimized geometries for all minima and TSs are provided in the Supporting Information. The conversion proceeds through distortion of the tpy ligand, with calculated activation barriers of 1.2 kcal mol^–1^ for **1** and 3.6 kcal mol^–1^ for **2**. These values closely match Turro’s experimental estimates of 1.67 ± 0.10 kcal mol^–1^ and 3.49 ± 0.82 kcal mol^–1^, respectively.^21^

We made several attempts to locate ^3^MC excited states characterized by elongation of the Ru–ACN bond, but all optimizations converged to the tpy-distorted ^3^MC states described above. However, the accessibility of these states, and the fact that in **1** the ^3^MC lies below the ^3^MLCT in energy, does not necessarily imply that they promote ACN dissociation, unless a corresponding transition state is identified. Furthermore, examination of the singly occupied molecular orbitals (SOMOs, Figure S1) reveals a more complex scenario: in **2**, the SOMO displays σ-antibonding character with respect to the Ru–ACN bond, whereas in **1** no such antibonding contribution is observed. The presence of an antibonding orbital in **2** may therefore suggest a greater propensity for dissociation, in line with the quantum yields reported by the Turro’s group.^21^

Taken together, these elements provided a stronger rationale for carrying out a full mechanistic search for ACN photodissociation from the ^3^MC states and therefore, model the second step of the reaction.

### ^3^MC-Driven Photodissociation Pathway

Photodissociation of a monodentate ligand in Ru polypyridyl complexes can proceed via dissociative, associative, or associative interchange pathways, yielding penta- or heptacoordinated intermediates, or no discrete intermediate, respectively. Since recent work on Ru­(II) thioether derivatives suggests that analogues of **1** and **2** most likely dissociate via a pentacoordinated Ru intermediate;^28^ we therefore focus on this pathway. In contrast to thioether systems, where the reactive ^3^MC state features pronounced Ru–S elongation, **1** and **2** show only modest Ru–ACN elongation, limiting water access to the metal center and disfavoring associative and interchange mechanisms.

Figure 4 reports the free-energy profiles for both complexes, showing the pathway to the penta-coordinated ^3^Ru_P_ species with ACN released from the Ru center. The figure also depicts the full dissociation process, from the initial ^3^MLCT-to-^3^MC conversion to the pentacoordinated species (Ru_P_) and the final aquation product (WAT), with all the relative energies calculated with respect to the ground state (GS). Key structural parameters for the intermediates involved in the conversion from ^3^MC to WAT are summarized in Tables S2 and S3 for **1** and **2**, respectively.

**Figure 4:**
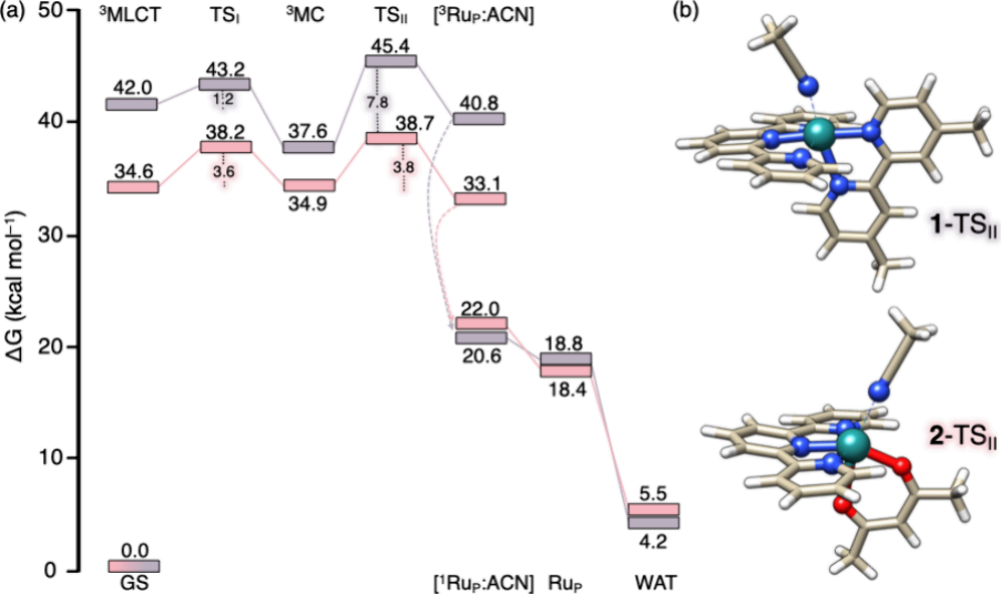
(a) Free-energy reaction profiles (kcal mol^–1^) for the ACN photodissociation mechanism of **1** (mauve) and **2** (pink). (b) TS_II_ structures of **1** and **2** corresponding to the ^3^MC → ^3^Ru_P_ step of acetonitrile dissociation.

The localized TS_II_ for the ^3^MC → ^3^Ru_P_ process shows imaginary frequencies of 96*i* cm^–1^ and 113*i* cm^–1^ for **1** and **2**, respectively; the associated modes couple ACN dissociation with tpy replanarization, suggesting that restoration of tpy planarity provides a driving force for ligand release. In **1**, ACN photodissociation proceeds with a 7.8 kcal mol^–1^ energy barrier, yielding [**1**-^3^Ru_P_:ACN], which is 3.2 kcal mol^–1^ less stable than **1**-^3^MC. Kinetically, this second step is rate-determining relative to the previously discussed internal conversion because it requires surmounting a higher barrier. In **2**, the corresponding barrier is 3.8 kcal mol^–1^; thus, the rate is governed by both steps, which have comparable barriers. The [**2**-^3^Ru_P_:ACN] product is 1.8 kcal mol^–1^ more stable than **2**-^3^MC, rendering this step slightly exergonic. Overall, for both **1** and **2**, the pathway from the ^3^MLCT entrance channel to the final [^3^Ru_p_:ACN] species is mildly favorable, consistent with formation of pentacoordinated Ru complexes.

These free-energy profiles support an alternative, plausible mechanism for ACN photodissociation in Ru-tpy complexes that differs from the ^3^MLCT-triggered pathway proposed by Turro. They indicate that ACN dissociation can be rate-determining or can compete with internal conversion. Consistent with experiments, the higher TS_II_ barrier in **1** than in **2** rationalizes the lower Φ of the former, despite its faster ^3^MLCT → ^3^MC conversion, as also predicted by our calculations. Thus, photodissociation efficiency likely depends on multiple factors, and Φ may not correlate directly with the ^3^MLCT → ^3^MC conversion alone. This interpretation is consistent with recent work by Buda and Bonnet, who combined static DFT and QM/MM calculations on related tridentate complexes and concluded that photosubstitution efficiency is governed by additional factors beyond the ^3^MLCT/^3^MC IC barrier.^28^

After forming the ^3^Ru_P_ intermediates, the systems relax to the ground-state ^1^Ru_P_ complexes. A water molecule then coordinates in a barrierless process, yielding the aqua complexes **1-WAT** and **2-WAT**. All attempts to locate [**1**/**2-** ^1^Ru_P_:**1**/**2-WAT**] adducts collapsed into the aqua species, and both reactions are exergonic by 14.6 and 12.9 kcal mol^–1^. A relaxed scan of water coordination (Figure S2) shows a continuous energy decrease for both **1** and **2** as the ligand approaches Ru, confirming the absence of a barrier.

To validate the scope of this mechanism, we examined complexes **3** and **4** (Figure 2). Although electronically similar to **2**, complex **3** exhibits a Φ value of only 0.0012, an atypical result within the acac-based series, about an order of magnitude lower than predicted by Turro’s Φ/^3^MLCT-^3^MC IC relationships and the experimental values reported for this family.^20^ Complex **4**, a derivative of **2**, was designed with structural modifications to reduce the energy required for the ^3^MLCT → ^3^MC process while maintaining the ACN dissociation energy characteristic of this class. Methyl substituents on the lateral pyridyl rings of the tpy introduce steric hindrance that facilitates IC between these two states.^9^ The rationale is that such a complex should combine a low conversion barrier, as in bpy-based systems, with the high quantum yield typical of the acac series. As for **1** and **2**, we computed the photodissociation pathways to the ^3^Ru_P_ of **3** and **4**. The calculated free-energy profiles are shown in Figure 5, and the corresponding energetic data and key structural parameters for the intermediates involved are summarized in Tables S4 and S5.

**Figure 5:**
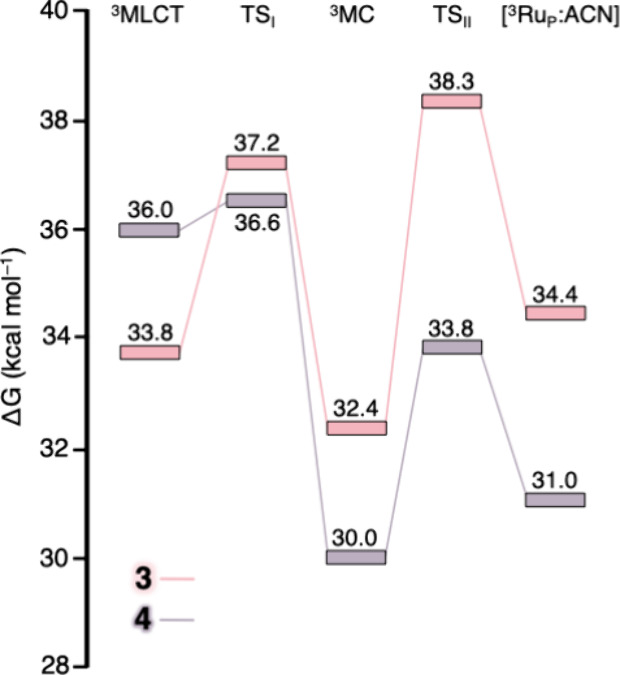
Free-energy reaction profiles (kcal mol^–1^) for the ^3^MLCT → ^3^MC → ^3^Ru_P_ of **3** and **4**. Energy values referred to the corresponding GS.

In close analogy with **2**, the ^3^MLCT-^3^MC conversion of **3** requires overcoming an activation barrier of 3.4 kcal mol^–1^. This process involves only a slight increase of the Ru–N1 bond (0.020 Å), while the Ru–N2 and Ru–N4 bonds elongate more significantly (0.295 Å and 0.311 Å, respectively). The ^3^MC intermediate is stabilized by 1.4 kcal mol^–1^ relative to the ^3^MLCT excited state. The calculated activation energy agrees with the experimental trend of internal conversion reported by Turro for this series,^21^ although no specific barrier has been published for this complex. Interestingly, ACN dissociation shows a different behavior compared to **2**. In **3**, the energy barrier is 5.9 kcal mol^–1^, higher than in the case of **2** (3.8 kcal mol^–1^) and similar to that of **1**, whose quantum yield also resembles **3**. Moreover, the reaction is endergonic by 2.0 kcal mol^–1^. These results may explain why **3** displays an anomalous value within the acac-based series. Altogether, the data reinforce the idea that the quantum yield of substitution is governed more by the ability of the ^3^MC state to promote acetonitrile dissociation than by the ^3^MLCT → ^3^MC IC, with dissociation acting as the rate-determining step in this complex.

In accordance with our design, the ^3^MLCT → ^3^MC process for **4** proceeds with a very low barrier of only 0.6 kcal mol^–1^ and is exergonic by 6.0 kcal mol^–1^. As in **2**, the highest SOMO shows σ-antibonding character on Ru–N1 bond, indicating that the resulting ^3^MC state is dissociative (Figure S3). The reaction then evolves from ^3^MC to ^3^Ru_P_, overcoming a 3.8 kcal mol^–1^ energy barrier, with the pentacoordinate product lying 1.0 kcal mol^–1^ higher in energy. Nevertheless, the overall process remains exergonic by 5.0 kcal mol^–1^.

In **2**, the rate-determining step is ambiguous, as both light-driven processes display similar barriers (3.6 vs 3.8 kcal mol^–1^). By contrast, the introduction of two ortho methyl groups in **4** lowers the ^3^MLCT-^3^MC IC barrier substantially, establishing ACN dissociation as the limiting step in the photoactivation pathway. If synthesized and studied under conditions similar to those adopted by Turro and co-workers,^21^ **4** would be expected to display a very low activation energy while achieving a quantum yield comparable to, or possibly exceeding, that of the parent acac series.

To confirm that these results arise solely from the steric effect of the methyl groups in the ortho positions, the same analysis was performed for **5** (Figure 2), an analogue of **4** with methyl groups in the meta positions. The free-energy profile for **5** (Figure S4) shows no significant differences compared to **2**, supporting the conclusion that steric hindrance in **4** facilitates ^3^MLCT → ^3^MC IC.

### Probing the ^3^MLCT-Driven Photodissociation Pathway

The results in the previous section show that photodissociation via the tpy-distorted ^3^MC state is an accessible pathway for this series of complexes. However, this does not exclude participation of a direct ^3^MLCT channel. It is therefore important to test explicitly whether ACN release can occur directly from the ^3^MLCT state, particularly because, to our knowledge, this pathway has not been located or characterized by DFT for Ru complexes. Dedicated calculations on the ^3^MLCT route are thus needed to assess its viability and clarify its possible contribution to the overall mechanism.

Optimization of ACN-release transition states originating directly from the ^3^MLCT pathway proved challenging (*vide infra*), as all attempts to optimize these transition states end up converging to the TS_II_ previously described. Therefore, to directly compare the two possible mechanisms, relaxed scans along the Ru–N1 bond were performed starting from both the ^3^MLCT and ^3^MC states, as shown in Figure 6 and Figure S5. The ^3^MC and ^3^MLCT scans are shown in pink and mauve, respectively. Structural data and SD data of the energy maximum in the relaxed energy scan are reported in Table S6 of the Supporting Information, while the molecular structures are reported in Figures S6 and S7.

**Figure 6:**
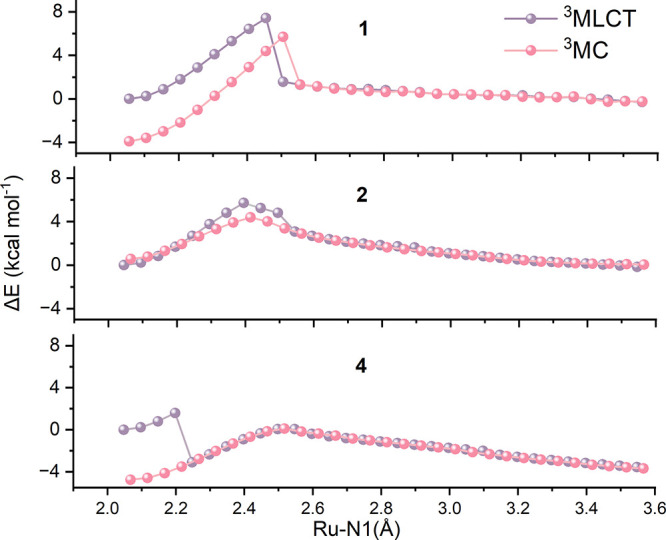
Relaxed energy scans for complexes **1, 2** and **4**. Pink: scan from ^3^MC triplet; mauve: scan from ^3^MLCT. Energies in kcal mol^–1^.

For **1**, given the energy difference highlighted above between the two triplet states, the relaxed ^3^MC scan starts at lower energy than the ^3^MLCT scan, and both profiles increase monotonically along the Ru–N1 coordinate up to 2.4 Å (^3^MLCT) and 2.5 Å (^3^MC). The ^3^MLCT profile reaches a maximum of 7.4 kcal mol^–1^, whereas the ^3^MC profile peaks at 5.7 kcal mol^–1^ (ΔE = 9.6 kcal mol^–1^), indicating that ACN dissociation is more favorable from ^3^MLCT than from ^3^MC. In contrast, for **2** the ^3^MC state lies close in energy to ^3^MLCT, only 0.6 kcal mol^–1^ higher, consistent with the internal conversion data (Table 1). In both relaxed scans, the maximum occurs at a Ru–N1 distance of 2.4 Å, with barriers of 5.7 and 3.8 kcal mol^–1^ from ^3^MLCT and ^3^MC, respectively. The mechanistic difference is evident at the maxima: the tpy ligand remains essentially planar in the ^3^MLCT scan, whereas the ^3^MC scan shows pronounced tpy distortion and Ru–N2/N4 elongation, as in TS_II_.

Ru spin densities at the maxima of the ^3^MLCT scans (along Ru–N1) are characteristic of ^3^MLCT character in both complexes (1.056 and 1.052 au for **1** and **2**, respectively). Moreover, after ∼ 2.4 Å, the energy decreases smoothly without trapping in another ^3^MC minimum, indicating that dissociation can proceed directly from ^3^MLCT to the ^3^RuP product. By contrast, the maxima in the ^3^MC scans, though similar in Ru–N1 distance and energy, show clear ^3^MC character, with Ru spin densities of 1.791 and 1.725 au for **1** and **2**. Beyond ∼ 2.6 Å the ^3^MC and ^3^MLCT profiles converge, further supporting access to ^3^RuP from the ^3^MLCT channel.

Overall, the relaxed scans of **1** and **2** support the viability of a ^3^MLCT pathway, but the small differences between the competing maxima are within typical DFT uncertainty, preventing a definitive assignment of the favored route. Both pathways, therefore, appear plausible and competitive for this class of compounds. Importantly, the scans remain consistent with the experiments: **2** shows a higher propensity for ACN dissociation than **1**, in line with its lower activation barriers.

Interestingly, **4** shows an unexpected behavior. Consistent with the free-energy plots in Figure 5, the ^3^MC scan starts 4.8 kcal mol^–1^ below the ^3^MLCT scan, rises to a maximum at Ru–N1 = 2.5 Å (ΔE = 4.8 kcal mol^–1^), and then decreases toward the ^3^RuP product. In contrast, the ^3^MLCT scan undergoes early internal conversion to the tpy-distorted ^3^MC state after only a few steps (Ru–N1 = 2.25 Å, ΔE = 1.6 kcal mol^–1^), as indicated by elongation of the Ru–N2 and Ru–N4 bonds from the fourth point onward; the distortion increases in subsequent steps and the two scan profiles begin to overlap. This behavior suggests that the bulky methyl substituents drive tpy distortion and favor ^3^MC population by relieving steric strain. The resulting overlap indicates that targeted ligand modifications can bias the dissociation mechanism. In particular, ortho-methyl substituents, known to promote internal conversion, appear to shift the balance toward the ^3^MC-mediated pathway, supporting the idea that structural tuning can selectively favor one accessible route over the other in this class of complexes. Energy scans for **3** (Figure S5) closely resemble those of **2**, with ^3^MC slightly below ^3^MLCT and barriers of 5.7 and 3.8 kcal mol^–1^ along the ^3^MLCT and ^3^MC pathways, respectively. This appears inconsistent with the free-energy profile in Figure 5, where **3** shows a higher ACN dissociation barrier than **2**. The difference arises because the scans omit thermal corrections: using electronic energies for **3**-^3^MC and **3**-TS_II_ gives ΔE ≈ 3.3 kcal mol^–1^, versus the ΔG^‡^ value of 5.9 kcal mol^–1^ reported in Figure 5, highlighting the importance of thermal contributions for **3.**

The relaxed scans reveal two possible routes for acetonitrile dissociation: a ^3^MC-mediated pathway, fully characterized in the previous section, and a ^3^MLCT-mediated pathway in which acetonitrile dissociates directly, without thermal population of a ^3^MC state. The maxima along the ^3^MLCT scans for **1**–**3** were used as starting points to locate the corresponding transition state. A distinct TS (TS_III_) was localized only for **1**, whereas for **2** and **3** the optimizations converged back to TS_II_. Figure 6 rationalizes this outcome. In **2** and **3**, the ^3^MC and ^3^MLCT scan maxima are nearly coincident in Ru–N1 distance and very close in energy (with the ^3^MC maximum slightly lower), so TS searches started from the ^3^MLCT maximum tend to converge to the lower-energy saddle point, TS_II_. In **1**, the maxima occur at clearly different Ru–N1 distances, allowing TS_III_ to be located.

Table 2 summarizes key geometric parameters and the activation and reaction free energies for **1**-TS_III_, and Figure 7 reports the spin-density surface, including **1**-TS_II_ for comparison. The SOMOs (Figure S8) show clear Ru–N1­(ACN) σ-antibonding character in both transition states. In **1**-TS_II_, an additional antibonding character appears on the Ru–N2/N4 interactions of tpy, consistent with reorganization of these bonds, whereas in **1-**TS_III_ they remain bonding.

**Table 2:** Selected Geometrical Parameters for TS_II_ (ACN Dissociation from the ^3^MC State) and TS_III_ (ACN Dissociation from the ^3^MLCT State) of **1** ^a^

1		
	TS_II_	TS_III_
Ru–N1	2.273	2.361
Ru–N2	2.228	2.106
Ru–N3	2.033	1.996
Ru–N4	2.153	2.108
Ru–N5	2.346	2.094
Ru–N6	2.125	2.103
N1–Ru–N3	87.5	81.6
N2–Ru–N4	146.6	156.8
N3–Ru–N5	103.2	104.4
N3–Ru–N6	176.4	178.3
N2–C–C–N3	–6.9	3.1
SD (Ru)	1.764	1.294
ΔG^‡^	7.8	6.4
ΔG_r_	5.1	0.9

a Bond distances are given in Å and angles in degrees. Activation (ΔG^‡^) and reaction (ΔG_r_) free energies are reported in kcal mol^–1^. SD on the Ru center is also provided. Atom numbering follows that used in Figure 3.

**Figure 7:**
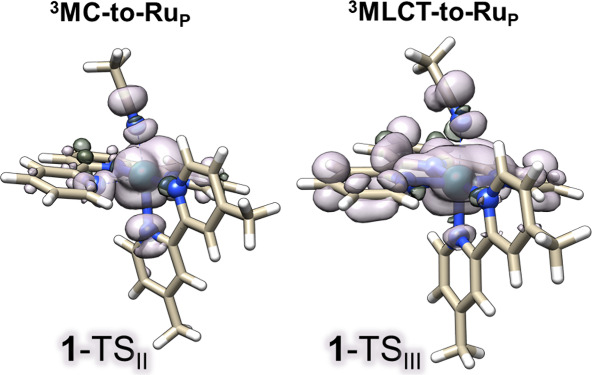
Spin density surfaces of TS_II_ and TS_III_ for complex **1**.

To our knowledge, **1-**TS_III_ is the first DFT-localized transition state for photodissociation initiated from a ^3^MLCT state in Ru polypyridyl complexes. **1-**TS_III_ lies 6.4 kcal mol^–1^ above the ^3^MLCT minimum, making this route slightly more accessible than the ^3^MC pathway, which involves sequential barriers of 1.2 and 7.8 kcal mol^–1^. TS_III_ is also the highest stationary point on the free-energy profile, consistent with the relaxed scan and supporting the scans as a qualitative mapping tool. Structurally, the key difference between the ^3^MLCT- and ^3^MC-derived transition states is the tpy framework: TS_II_ features pronounced tpy distortion, whereas TS_III_ largely preserves tpy planarity.

## Conclusions

Our calculations provide mechanistic insight into ACN photodissociation in Ru-tpy complexes, focusing on the representative bpy (**1**) and acac (**2, 3**) derivatives from Turro’s series. The computed ^3^MLCT-^3^MC internal-conversion barriers reproduce the experimental trends and indicate an atypical reactive ^3^MC minimum in which tpy distortion, rather than strong Ru–L elongation, is the dominant structural feature.

From this distorted ^3^MC state, we located a transition state for ACN loss leading to a pentacoordinated Ru species, showing that photosubstitution can proceed without invoking a canonical dissociative ^3^MC state characterized by a markedly elongated Ru–L bond. For **1** in particular, the ACN-photodissociation barrier from ^3^MC is higher in energy than internal conversion, confirming that population of the ^3^MC states is an important factor in photodissociation but not the sole determining process. This implies that the ligand substitution quantum yields cannot be directly correlated with the internal conversion process for this series, as ACN dissociation can strongly influence the overall quantum yield. Therefore, caution should be exercised when interpreting these results, since for some complexes the internal conversion may not represent the rate-determining step in the photodissociation process.

We also evaluated direct ACN dissociation from the ^3^MLCT state using relaxed scans and transition-state searches. The results suggest a competitive interplay between ^3^MLCT-mediated pathways, as previously hypothesized by Turro and co-workers, and ^3^MC-mediated pathways. Due to the small energetic differences between competing pathways and the inherent limitations of the DFT approach, the mechanistic conclusions should be regarded as qualitative and mechanistically suggestive.

Finally, we designed complex **4**, an ortho-dimethyl derivative of **2**, to probe steric effects. The methyl groups facilitate ^3^MLCT-^3^MC conversion and stabilize the distorted ^3^MC state, while leaving the ACN dissociation barrier comparable to **2**. The scans indicate that ligand-induced tpy distortion can bias the mechanism, illustrating how targeted tpy substitution can shift the balance between competing dissociation pathways. Overall, both ^3^MC- and ^3^MLCT-mediated pathways are consistent with the available evidence, although direct ^3^MLCT dissociation is most clearly supported for **1** rather than established generally across the series.

## Methods and Experimental Details

### Computational Methods

Geometry optimizations for all molecules were carried out in water to reproduce as better as possible physiological conditions, using the polarizable continuum model^29^ (PCM) of Tomasi and the B3LYP functional^30,31^ within the Gaussian16 computational package.^32^ Dispersion corrections for noncovalent interactions were accounted for through Grimme’s D3 atom-pairwise additive scheme.^33^

The 6–31G** basis set was adopted for all atoms other than ruthenium, which was described using the relativistic Stuttgart/Dresden (SDD)^34^ effective core potential (ECP) with its corresponding split-valence basis set. The use of ECPs for metal centers is a widely adopted approach in computational studies of Ru complexes, enabling a reliable description of geometries, relative energies, and ground- and excited-state electronic structure. Moreover, the reliability of the selected computational protocol in reproducing the behavior of these complexes is supported by comparison with experimental internal conversion data, where the protocol accurately reproduces the reaction energetics.

The identification of TSs was performed by designing plausible initial geometries resembling the expected TS structures. Vibrational frequency analyses at the same level of theory were then carried out to confirm the nature of each stationary point characterized by zero imaginary frequencies for minima and a single imaginary frequency for TSs. The TSs were properly connected to the corresponding energy minima through intrinsic reaction coordinate (IRC) analysis.^35^ Relaxed energy scans along the Ru–ACN bond distance were performed to investigate ACN dissociation from the ^3^MLCT and ^3^MC states. To reduce the computational cost, only electronic energies were evaluated for these scans.

Molecular graphics were generated using UCSF Chimera.^36^ Isodensity surfaces for spin densities were visualized with an isovalue of 0.02 e/Bohr^3^. Coordinates of all stationary points are provided as ; corresponding structures are shown in Figure S9.

## Supplementary Material





## Notes

Additional DFT data, including fully optimized geometries for all minima and transition states, selected geometrical parameters of ground- and excited-state structures and transition states, orbitals isosurfaces, free energy scans and profiles (PDF)
Cartesian Coordinates of all stationary points (TXT)
