# Supplementary material for: DFT Insights on Ligand Photodissociation Pathways in Ruthenium–Terpyridine Complexes: 3MLCT- or 3MC-Triggered?
Source: Inorg Chem. 2026 Apr 22;65(17):9568–76. doi: 10.1021/acs.inorgchem.6c00971 (PMC13147312; doi:10.1021/acs.inorgchem.6c00971)
Supplement: Supplementary file 1 [file ic6c00971_si_001.pdf]

Supporting Information

DFT Insights on Ligand Photodissociation Pathways in Ruthenium-Terpyridine Complexes: ^{3}MLCT- or ^{3}MC-Triggered?

Stefano Scoditti[a,b,c], Gloria Mazzone[c], Emilia Sicilia*,[c] Luca Salassa*[a,b,d]

[a] *Donostia International Physics Center, Paseo Manuel de Lardizabal 4, Donostia, 20018, Spain*

[b] *Polimero eta Material Aurreratuak: Fisika, Kimika eta Teknologia, Kimika Fakultatea, Euskal Herriko Unibertsitatea UPV/EHU, 20018 Donostia-San Sebastian, Spain*

[c] *Department of Chemistry and Chemical Technologies, Università della Calabria, Arcavacata di Rende (CS), 87036, Italy*

[d] *Ikerbasque, Basque Foundation for Science, Bilbao 48011, Spain*

Corresponding Authors
Emilia Sicilia – Email: emilia.sicilia@unical.it
Luca Salassa – Email: lsalassa@dipc.org

Table S1. Computed normal modes corresponding to the C–N stretching of acetonitrile, both in its isolated form and when coordinated in **1** and **2**, in their ground and ^{3}MLCT excited states. Reported values include C–N bond lengths (in Å); for the complexes, the Ru–N bond lengths are also provided.

	**ACN**	**1-GS**	**2-GS**	**1-^{3}MLCT**	**2-^{3}MLCT**
C-N (Å)	1.161	1.159	1.158	1.159	1.157
$V_{C\text{-}N}$ (cm^{-1})	2366	2364	2375	2392	2397
Ru-N (Å)	/	2.015	2.039	2.055	2.045

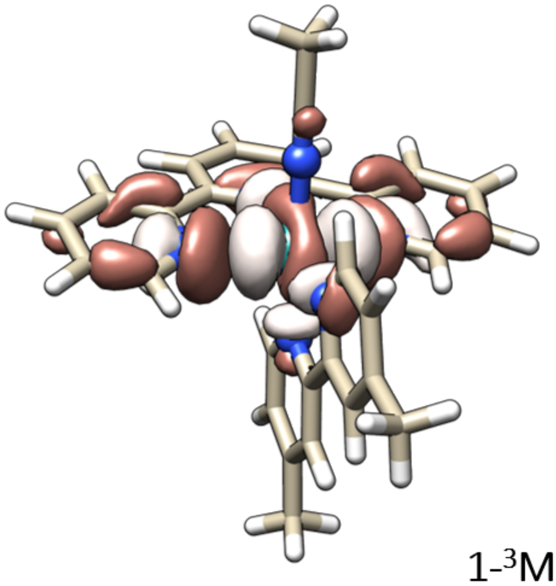

1-^{3}MC-SOMO

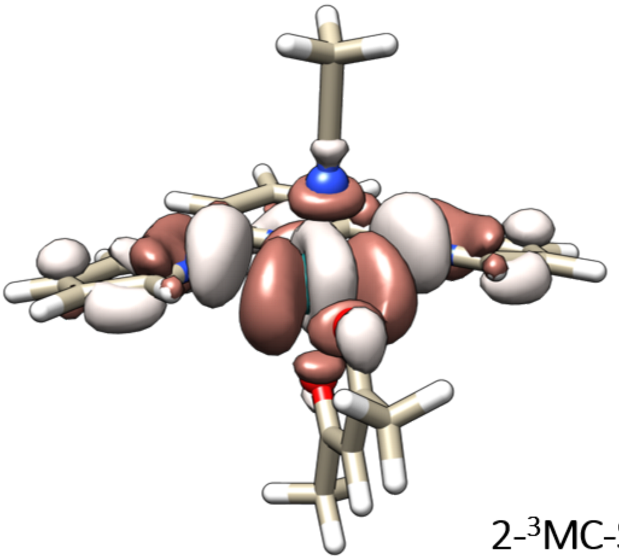

2-^{3}MC-SOMO

Figure S1. SOMOs plot for **1** and **2** complexes in their ^{3}MC excited state.

Table S2. Selected geometrical parameters for the stationary points shown in Figure 4 for **1** are reported. Bond distances are given in Å and angles in degrees. Activation ($\Delta G^{\ddagger}$) and reaction (ΔG_r) free energies for the ^{3}MC → ^{3}Ru$_P$ conversion in water are reported in kcal mol^{-1}. Spin densities (SD) on the Ru atom are provided for the triplet states.

^{3}MC → WAT			1			
	^{3}MC	TS$_{II}$	[^{3}RuP:ACN]	[^{1}RuP:ACN]	RuP	WAT
Ru–N1/O^{WAT}	2.055	2.273	3.581	3.596	/	2.191
Ru–N2	2.392	2.228	2.115	2.101	2.102	2.094
Ru–N3	2.256	2.033	1.998	1.999	1.999	1.989
Ru–N4	2.403	2.153	2.116	2.098	2.101	2.093
Ru–N5	2.085	2.346	2.272	2.084	2.008	2.044
Ru–N6	2.092	2.125	2.130	2.008	2.086	2.089
N1/O^{WAT}–Ru–N3	84.8	87.5	93.5	99.8	/	90.7
N2–Ru–N4	137.7	146.6	157.1	158.4	158.3	158.7
N3–Ru–N5	99.1	103.2	108.5	98.4	99.0	97.7
N3–Ru–N6	176.1	176.4	177.4	177.6	178.0	176.7
N2–C–C–N3	-11.1	-6.9	-0.3	1.4	1.7	1.4
SD (Ru)	1.789	1.764	1.845	/	/	/
$\Delta G^{\ddagger}$		7.8				
ΔG_r			3.2			

Table S3. Selected geometrical parameters for the stationary points shown in Figure 4 for **2** are reported. Bond distances are given in Å and angles in degrees. Activation ($\Delta G^{\ddagger}$) and reaction (ΔG_r) free energies for the ^{3}MC → ^{3}Ru$_P$ conversion in water are reported in kcal mol^{-1}. Spin densities (SD) on the Ru atom are provided for the triplet states.

^{3}MC → WAT			2			
	^{3}MC	TS$_{II}$	[^{3}RuP:ACN]	[^{1}RuP:ACN]	RuP	WAT
Ru–N1/O^{WAT}	2.065	2.407	3.754	3.590	/	2.182
Ru–N2	2.385	2.200	2.116	2.108	2.108	2.084
Ru–N3	2.188	2.032	1.994	1.978	1.977	1.960
Ru–N4	2.380	2.197	2.110	2.110	2.109	2.086
Ru–O5	2.043	2.086	2.165	2.019	2.017	2.043
Ru–O6	2.050	2.050	2.057	2.100	2.094	2.084
N1/O^{WAT}–Ru–N3	95.4	94.7	97.8	85.5	/	95.5
N2–Ru–N4	141.5	146.0	148.5	158.9	159.1	160.0
N3–Ru–O5	86.6	89.9	92.9	87.8	87.5	88.5
N3–Ru–O6	176.2	179.6	178.2	179.8	179.8	178.8
N2–C–C–N3	16.1	9.9	3.7	-1.4	-1.8	-1.4
SD (Ru)	1.762	1.630	1.803	/	/	/
$\Delta G^{\ddagger}$		3.8				
ΔG_r			1.8			

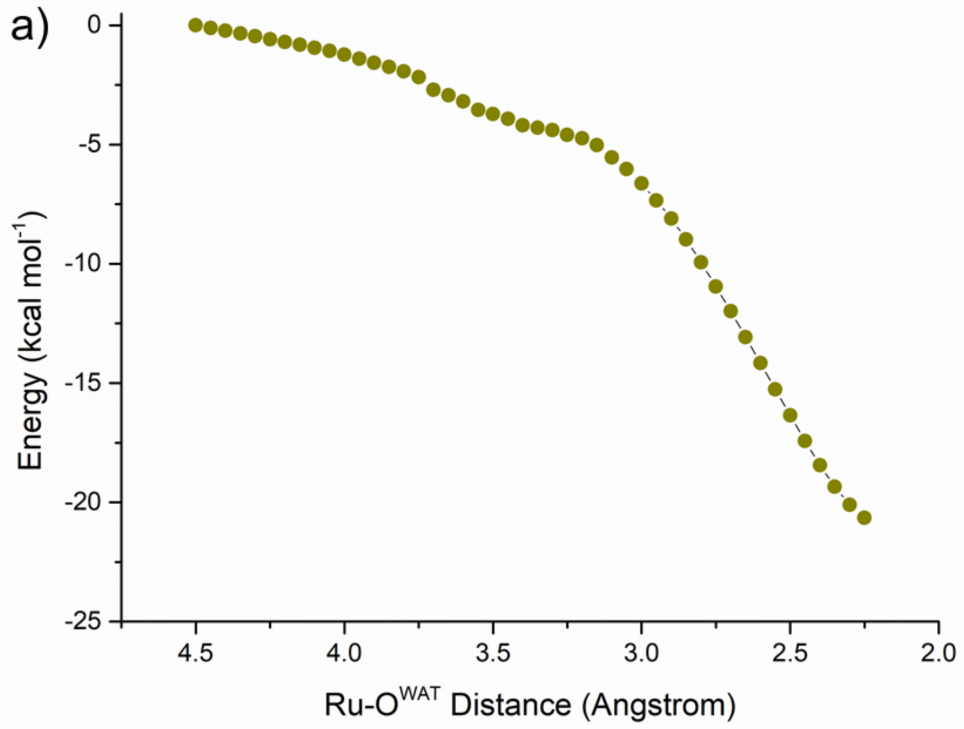

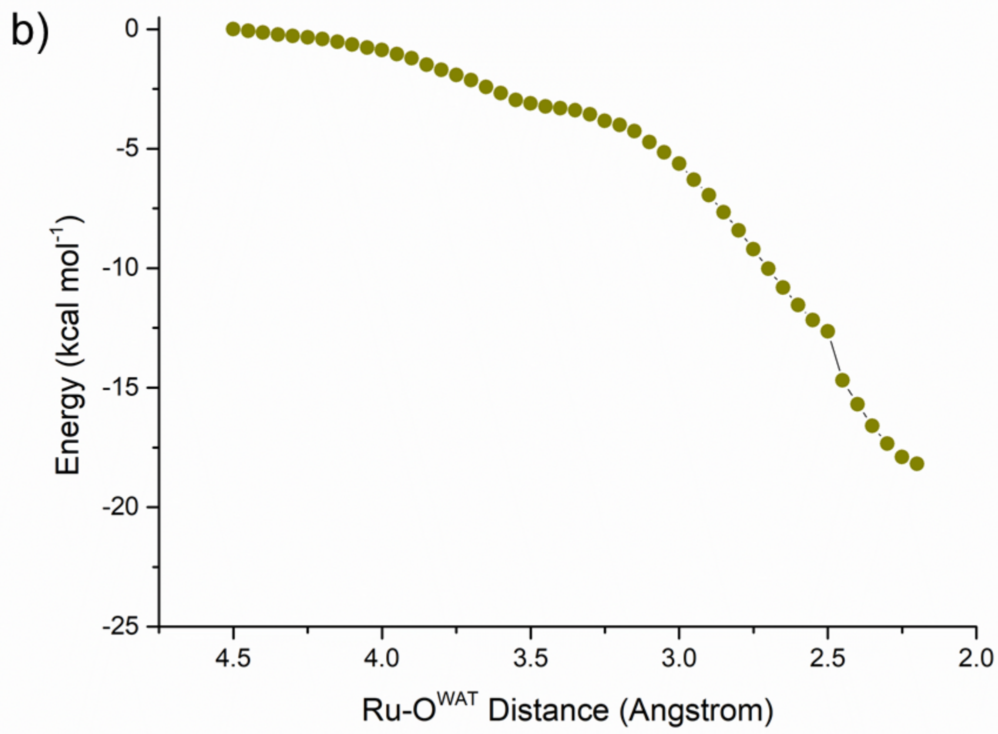

Figure S2. Relaxed scan of the Ru-WAT bond (0.05 Å each step) starting from 4.5 Å for (a) **1** and (b) **2**. Energies (in kcal mol^{-1}) are referenced to the first point of the scan at 4.5 Å.

Table S4. Selected geometrical parameters for the stationary points shown in Figure 5 for **3** are reported. Bond distances are given in Å and angles in degrees. Activation ($\Delta G^{\ddagger}$) and reaction (ΔG_r) free energies for ^{3}MLCT → ^{3}MC and ^{3}MC → ^3Ru_P conversions in water are reported in kcal mol^{-1}. Spin densities (SD) on the Ru atom are provided for the triplet states.

3						
	GS	^{3}MLCT	TS_I	^{3}MC	TS_{II}	[^{3}RuP:ACN]
Ru–N1	2.016	2.045	2.085	2.065	2.396	3.657
Ru–N2	2.093	2.085	2.216	2.380	2.211	2.087
Ru–N3	1.967	2.001	2.086	2.186	2.053	1.995
Ru–N4	2.089	2.081	2.202	2.392	2.378	2.175
Ru–O5	2.056	2.018	2.016	2.033	2.080	2.153
Ru–O6	2.094	2.041	2.041	2.041	2.051	2.052
N1–Ru–N3	93.8	95.0	96.0	95.7	97.5	87.8
N2–Ru–N4	159.9	155.4	147.5	141.8	142.5	145.3
N3–Ru–O5	87.0	92.4	86.0	86.3	89.6	93.3
N3–Ru–O6	177.5	177.0	177.2	177.0	177.2	178.0
N2–C–C–N3	-1.9	0.5	15.7	15.3	4.8	2.0
SD (Ru)	-	0.860	1.343	1.766	1.778	1.816
$\Delta G^{\ddagger}$			3.4		5.9	
ΔG_r				-1.4		2.0

Table S5. Selected geometrical parameters for the stationary points shown in Figure 5 for **4** are reported. Bond distances are given in Å and angles in degrees. Activation ($\Delta G^{\ddagger}$) and reaction (ΔG_r) free energies for ^{3}MLCT → ^{3}MC and ^{3}MC → ^3Ru_P conversions in water are reported in kcal mol^{-1}. Spin densities (SD) on the Ru atom are provided for the triplet states.

4						
	GS	^{3}MLCT	TS_I	^{3}MC	TS_{II}	[^{3}RuP:ACN]
Ru–N1	2.012	2.047	2.075	2.067	2.498	3.780
Ru–N2	2.155	2.128	2.193	2.416	2.234	2.148
Ru–N3	1.976	2.007	2.047	2.162	2.025	1.993
Ru–N4	2.155	2.128	2.193	2.416	2.287	2.209
Ru–O5	2.063	2.020	2.018	2.041	2.112	2.148
Ru–O6	2.097	2.053	2.050	2.049	2.053	2.060
N1–Ru–N3	94.3	96.6	96.3	96.5	96.0	103.0
N2–Ru–N4	159.0	154.7	149.9	141.3	144.4	142.6
N3–Ru–O5	86.8	84.1	85.0	85.6	88.7	88.1
N3–Ru–O6	177.3	174.6	175.7	176.4	178.0	176.8
N2–C–C–N3	-4.2	0.4	14.1	17.2	6.9	2.5
SD (Ru)	-	0.863	1.141	1.765	1.767	1.823
$\Delta G^{\ddagger}$			0.6		3.8	
ΔG_r				-6.0		1.0

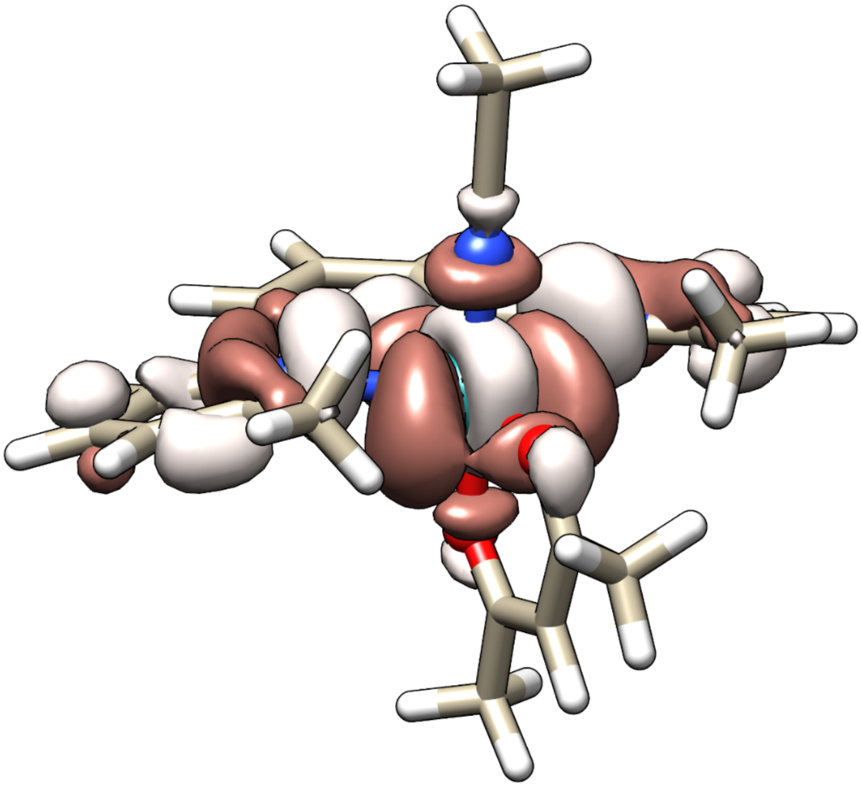

Figure S3. Singlet Occupied Molecular Orbital (SOMO) of **4**. The SOMO isosurfaces are shown at an isovalue of 0.02 e/Bohr^3.

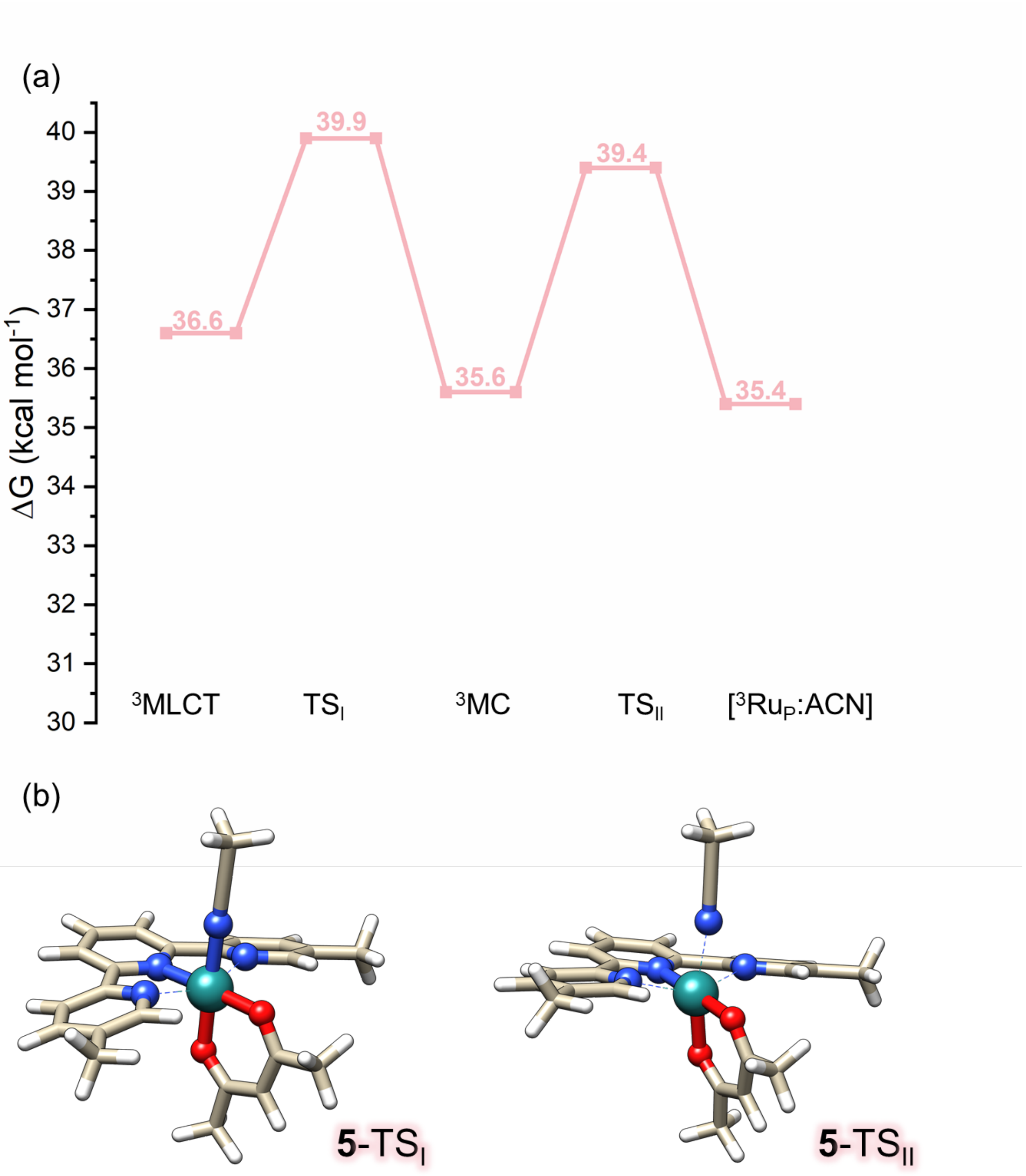

Figure S4. (a) Free-energy reaction profile (kcal mol^{-1}) for the ^{3}MLCT → ^{3}MC → ^{3}Ru$_P$ of **5**. Energy values referred to the corresponding GS. (b) TS$_I$ and TS$_{II}$ structures of **5** corresponding to ^{3}MLCT → ^{3}MC and ^{3}MC → ^{3}RuP steps respectively.

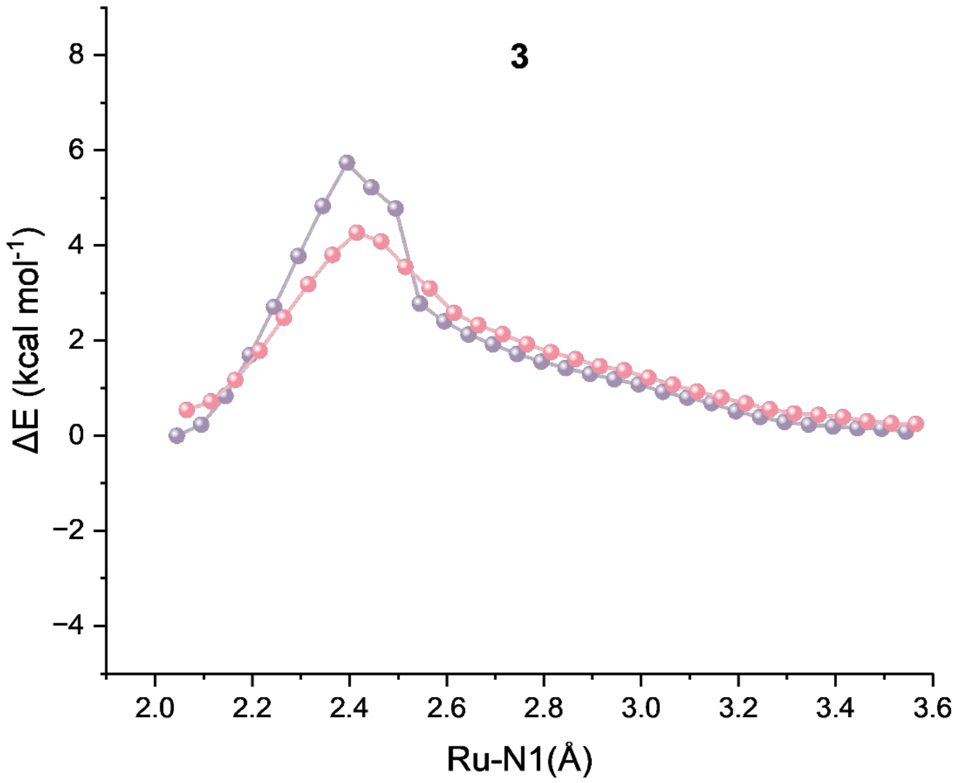

Figure S5. Relaxed energy scan for complex 3. Pink: scan from ^{3}MC triplet; mauve: scan from ^{3}MLCT. Energies in kcal mol^{-1}.

Table S6. Structural data and SD values corresponding to the energy maximum obtained from the relaxed energy scan for **1-4** complexes.

	**1**		**2**		**3**		**4**	
	^{3}MLCT	^{3}MC	^{3}MLCT	^{3}MC	^{3}MLCT	^{3}MC	^{3}MLCT[a]	^{3}MC
Ru–N1	2.404	2.505	2.395	2.415	2.395	2.415	2.197	2.517
Ru–N2	2.082	2.363	2.093	2.287	2.093	2.308	2.131	2.242
Ru–N3	2.011	2.187	2.007	2.078	2.008	2.079	2.010	2.018
Ru–N4	2.081	2.368	2.092	2.291	2.095	2.283	2.132	2.241
Ru–N5/O5	2.027	2.078	1.998	2.065	1.994	2.055	1.999	2.132
Ru–N6/O6	2.092	2.066	2.056	2.044	2.047	2.035	2.054	2.054
N1–Ru–N3	83.4	83.4	93.5	96.4	93.8	96.9	96.1	96.2
N2–Ru–N4	154.9	139.9	152.2	142.6	152.2	142.3	153.4	145.3
N3–Ru–N5/O5	100.8	100.5	88.4	88.3	88.3	88.0	84.5	89.2
N3–Ru–N6/O6	179.3	177.8	179.6	178.8	179.8	178.1	175.7	178.0
N2–C–C–N3	-1.7	-11.1	5.2	12.1	5.4	13.8	3.6	7.6
SD (Ru)	1.056	1.791	1.052	1.725	1.071	1.734	0.821	1.765

[a] here is reported the Maximum in energy relative to the conversion from the ^{3}MLCT to the ^{3}MC excited states.

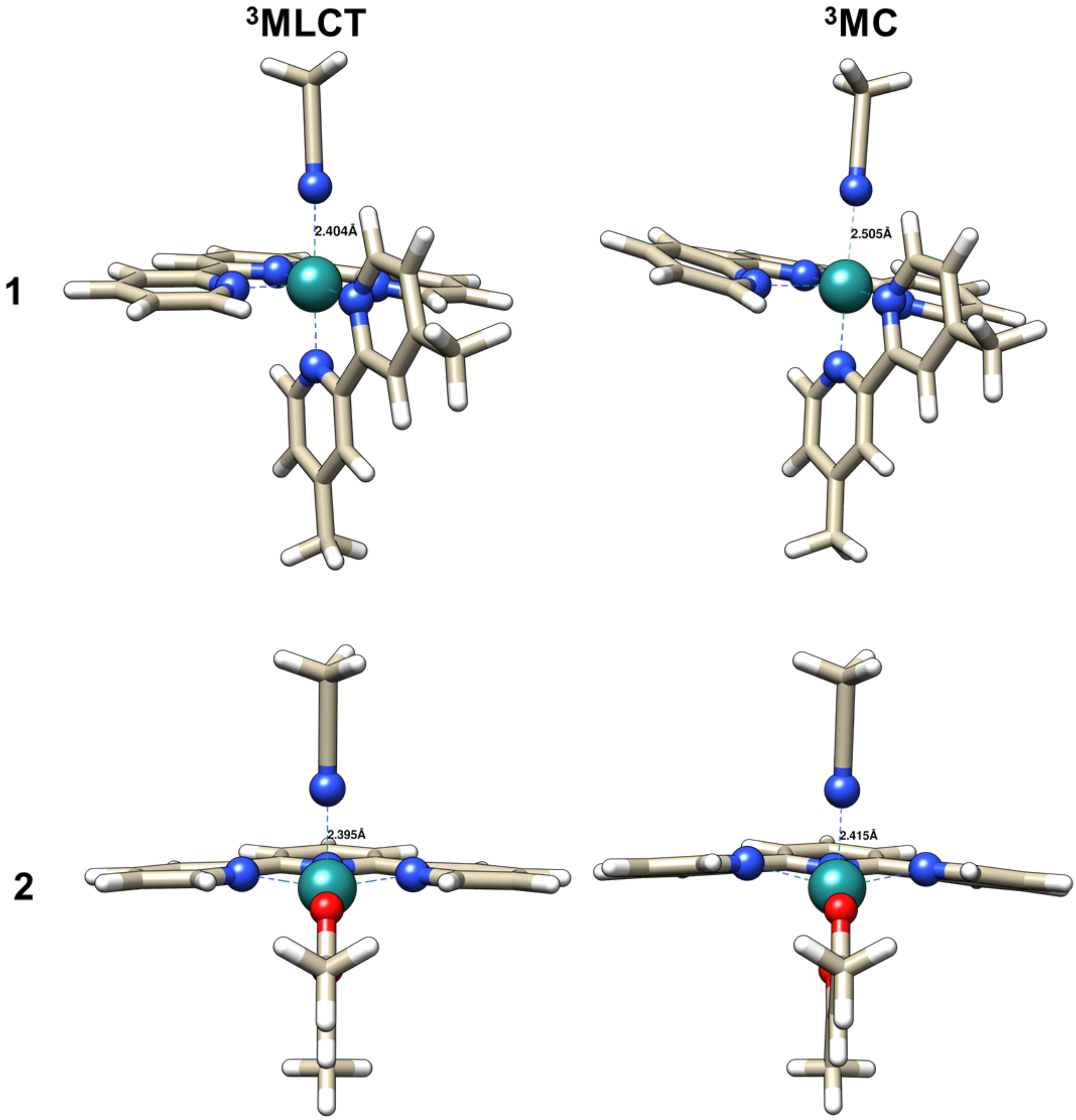

Figure S6. Molecular structures corresponding to the maximum energy points obtained from the relaxed scan along the Ru–N1 distance, starting from the ^{3}MLCT and ^{3}MC excited states, for complexes **1** and **2**. Ru-N1 distance is also showed in Angstrom (Å).

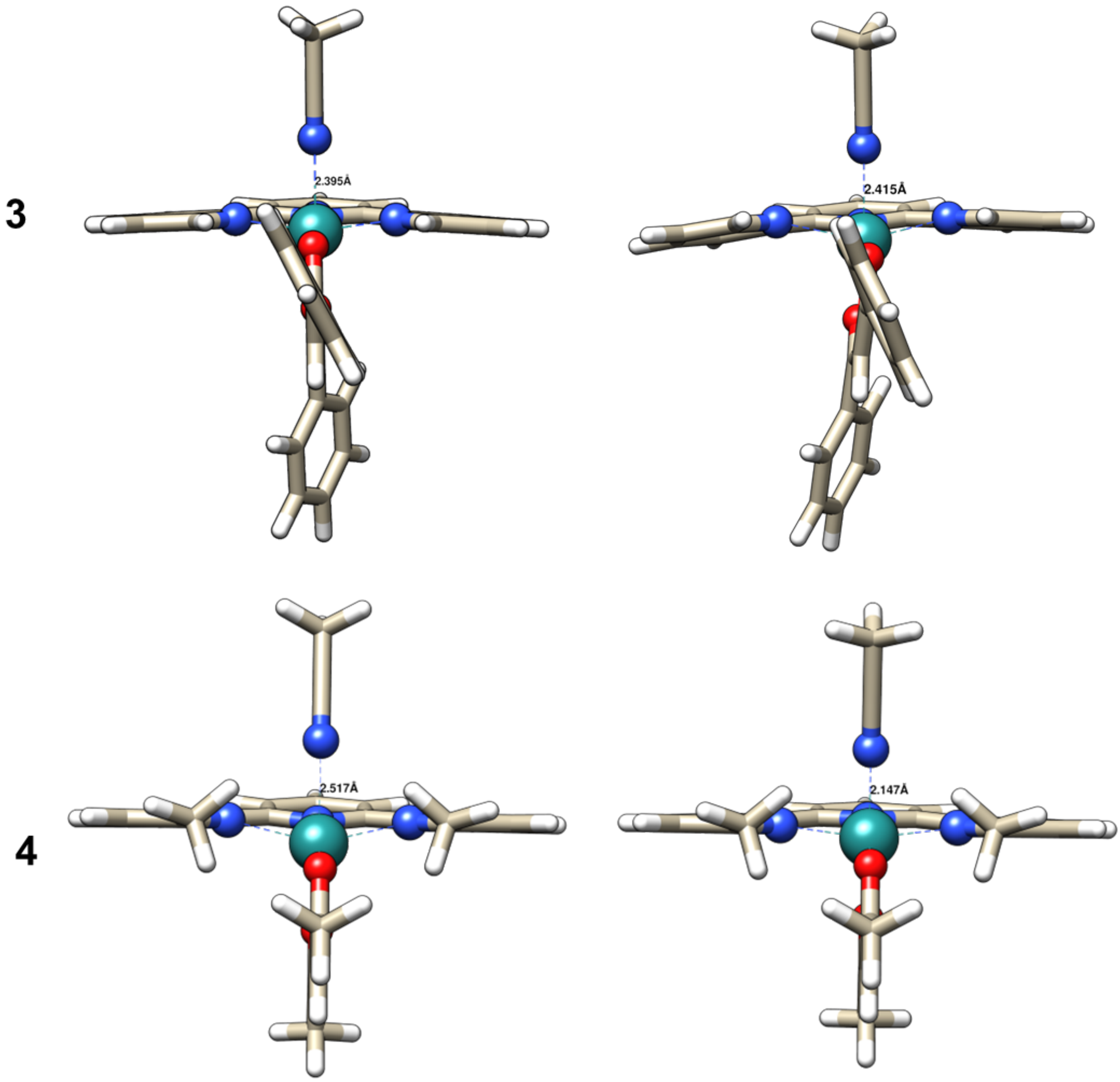

Figure S7. Molecular structures corresponding to the maximum energy points obtained from the relaxed scan along the Ru–N1 distance, starting from the ^{3}MLCT and ^{3}MC excited states, for complexes **3** and **4**. Ru-N1 distance is also showed in Angstrom (Å).

^{3}MC-to-Ru$_{P}$

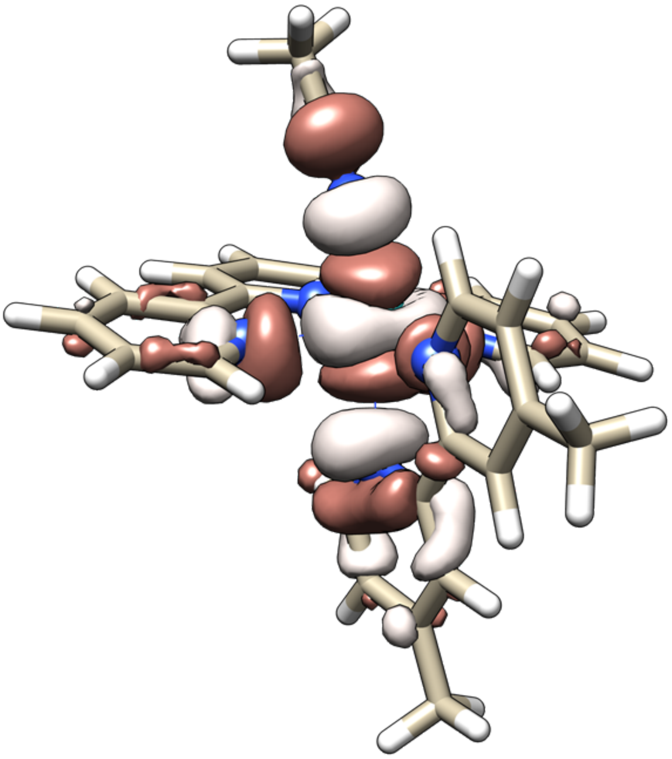

1-TS$_{II}$

^{3}MLCT-to-Ru$_{P}$

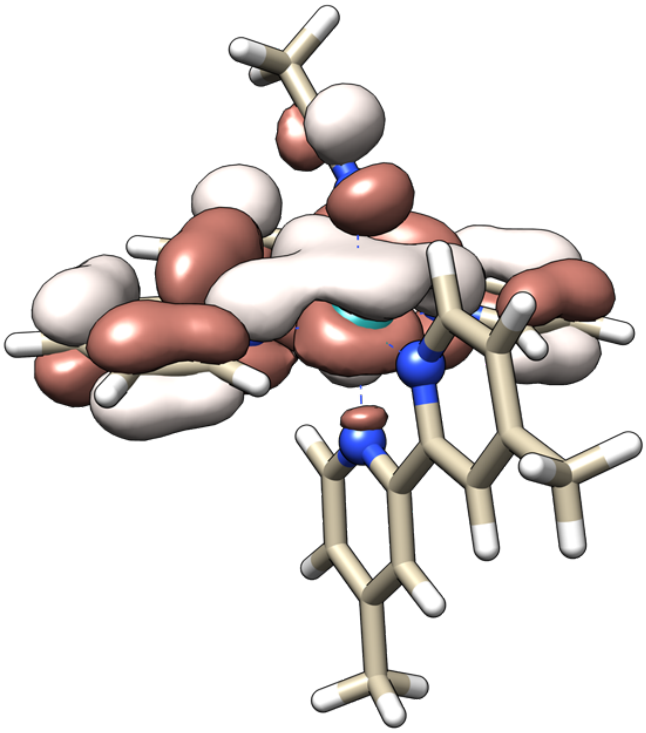

1-TS$_{III}$

Figure S8. SOMOs of TS$_{II}$ and TS$_{III}$ for complex **1**.

1-GS

2-GS

1-^{3}MLCT

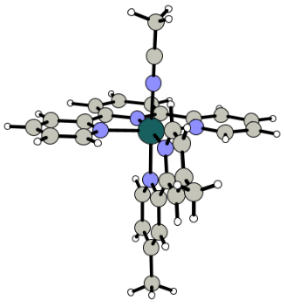

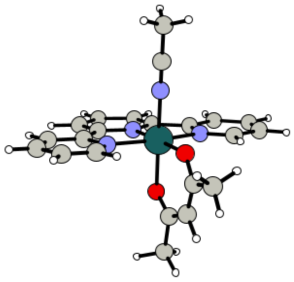

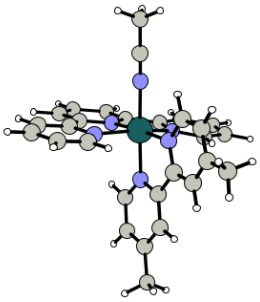

1-^{3}MC

2-^{3}MLCT

2-^{3}MC

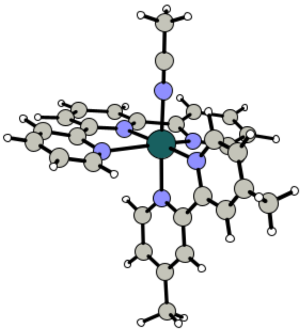

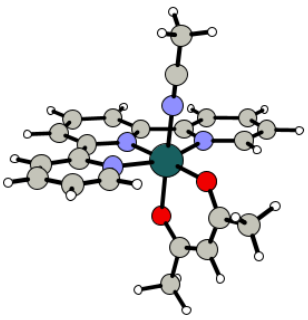

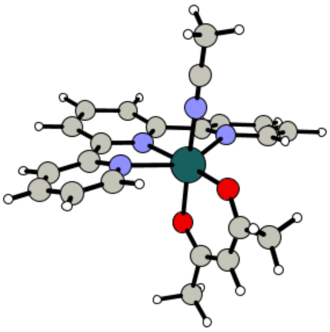

1-TS_I

2-TS_I

1-TS_{II}

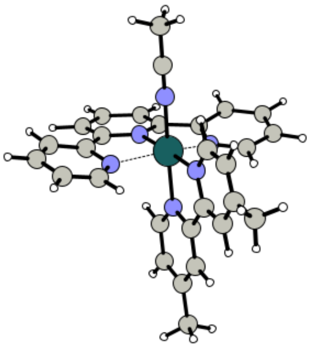

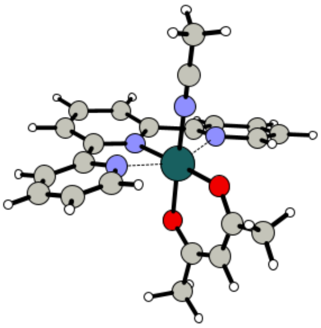

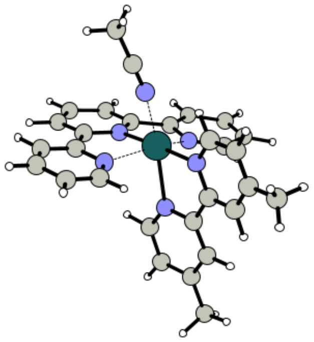

2-TS_{II}

1-[^3Ru_p:ACN]

2-[^3Ru_p:ACN]

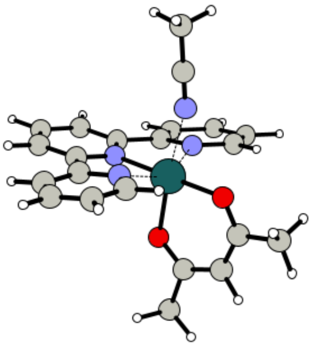

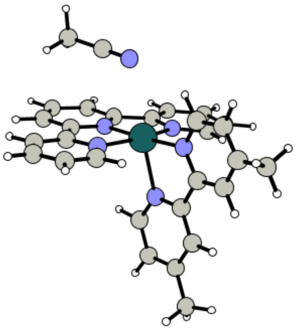

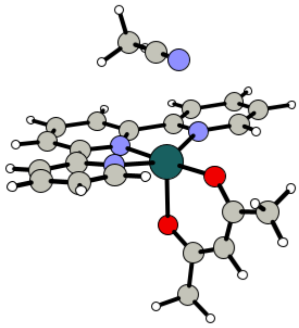

1-[^1Ru_p:ACN]

2-[^1Ru_p:ACN]

1-^1Ru_p

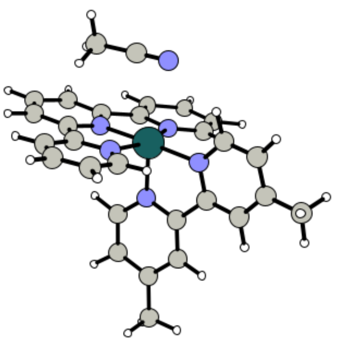

2-^{1}Ru$_{p}$

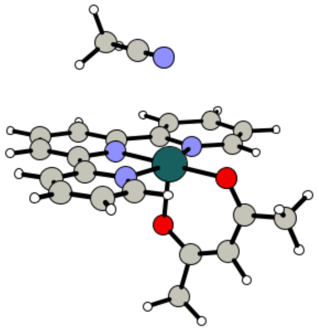

1-WAT

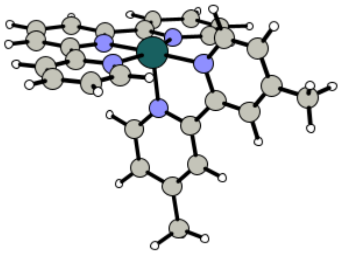

2-WAT

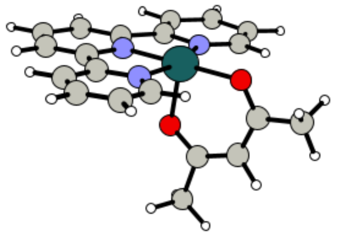

3-GS

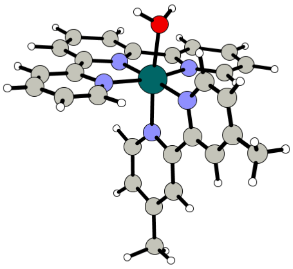

4-GS

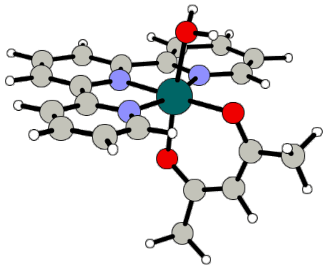

3-^{3}MLCT

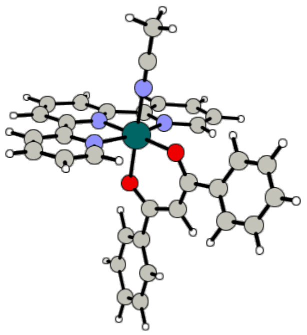

4-^{3}MLCT

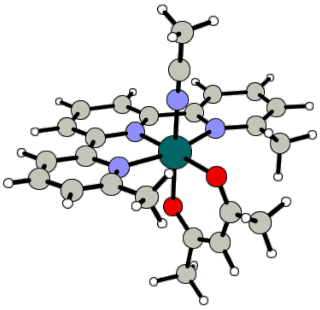

3-TS$_{I}$

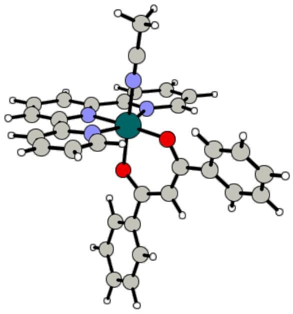

4-TS$_{I}$

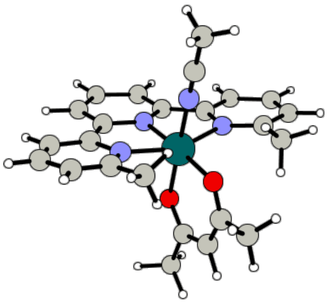

3-^{3}MC

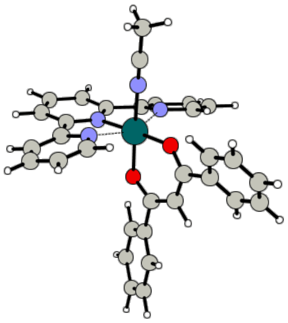

4-^{3}MC

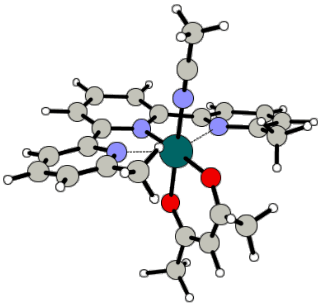

3-TS$_{II}$

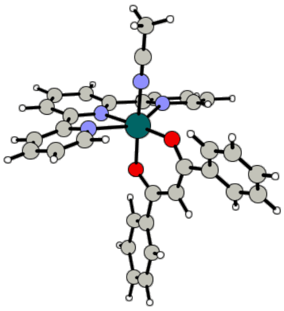

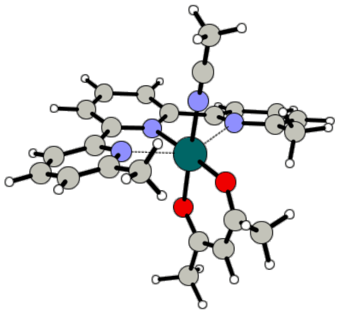

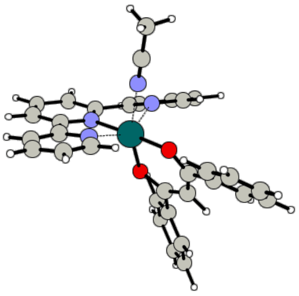

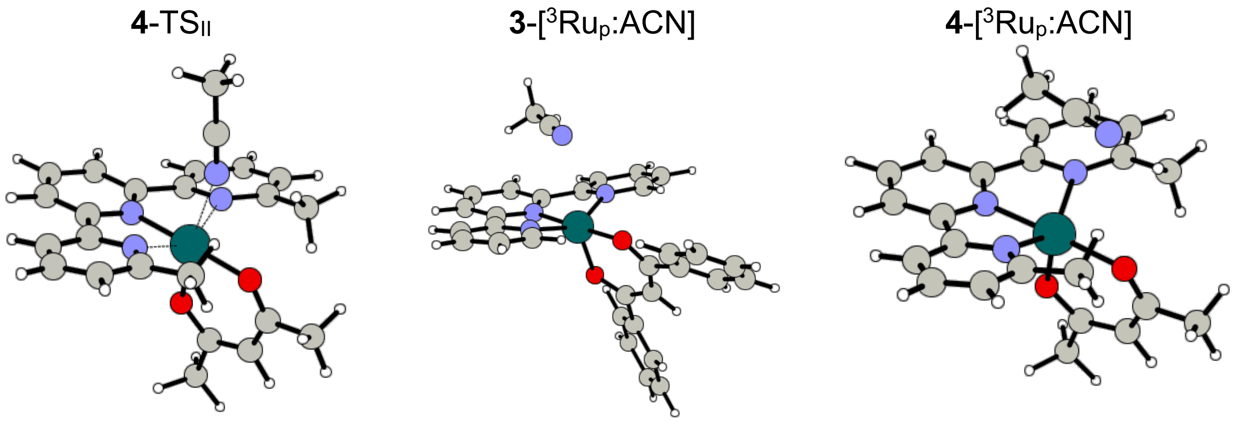

Figure S9. Optimized geometries of the stationary points of **1**-**4** discussed in this work.
