# Supplementary material for: DFT Insights on Ligand Photodissociation Pathways in Ruthenium–Terpyridine Complexes: 3MLCT- or 3MC-Triggered?
Source: Inorg Chem. 2026 Apr 22;65(17):9568–76. doi: 10.1021/acs.inorgchem.6c00971 (PMC13147312; doi:10.1021/acs.inorgchem.6c00971)
Supplement: Supplementary file 2 [file ic6c00971_si_002.txt]

1-GSRu                 0.26377800    0.00471600    0.43920100 C                 -0.22808400   -3.05636300    0.62228700 H                 -1.19082600   -2.75442600    1.01805400 C                  0.11299800   -4.39808700    0.47096300 H                 -0.59734400   -5.16681600    0.75174800 C                  1.36901700   -4.71744900   -0.04091400 C                  2.24321600   -3.68782900   -0.38628300 H                  3.22335700   -3.91610200   -0.78647100 C                  1.84658800   -2.36209600   -0.21404900 C                  2.69761300   -1.20108400   -0.55697100 C                  3.99112100   -1.23332200   -1.08203000 H                  4.48928800   -2.17436500   -1.27840500 C                  4.63678200   -0.02506300   -1.35085000 C                  4.00039100    1.19188100   -1.09969900 H                  4.50577500    2.12610300   -1.30964600 C                  2.70670400    1.17721700   -0.57433800 C                  1.86456300    2.34960100   -0.24867200 C                  2.27115700    3.66959500   -0.44077100 H                  3.25296600    3.88450500   -0.84428300 C                  1.40473300    4.71081900   -0.11093700 C                  0.14634300    4.40856900    0.40549100 H                 -0.55822400    5.18669900    0.67470000 C                 -0.20484600    3.07187500    0.57681400 H                 -1.16988500    2.78319200    0.97683800 N                  0.60866300   -2.05979500    0.29156600 N                  2.10391100   -0.00788400   -0.32531400 N                  0.62439500    2.06417300    0.26124800 C                 -2.17574900    0.02590800    2.34270700 H                 -1.40710700    0.03082400    3.10540500 C                 -3.52095600    0.03006600    2.67614100 C                 -4.49143400    0.02314600    1.66389900 C                 -4.03426200    0.01282800    0.34473500 H                 -4.75255300    0.00728400   -0.46589400 C                 -2.66576000    0.00942200    0.06547600 C                 -2.10673000   -0.00170800   -1.29919300 C                 -2.88562700   -0.00640900   -2.45758100 H                 -3.96597700   -0.00054200   -2.38110100 C                 -2.28925700   -0.01730500   -3.72081700 C                 -0.88876800   -0.01794800   -3.76564500 C                 -0.16166200   -0.01268900   -2.58561800 H                  0.92084900   -0.01234700   -2.59875900 N                 -1.74180100    0.01555700    1.06598100 N                 -0.74568000   -0.00534500   -1.37143800 C                  1.54523500    0.01764800    3.36767600 H                  5.64123800   -0.03186900   -1.75867500 H                 -3.80857400    0.03856200    3.72206200 H                 -0.36099600   -0.02097900   -4.71312500 C                  2.13554500    0.02073700    4.69741200 H                  2.93438800   -0.72381100    4.74865900 H                  2.54997100    1.00840900    4.91611500 H                  1.37099800   -0.22098700    5.44067200 N                  1.06459500    0.01430400    2.31410000 C                 -5.95861800    0.02496900    1.99508400 H                 -6.57637700    0.03681000    1.09473600 H                 -6.21916800   -0.86218100    2.58203700 H                 -6.21345400    0.89967300    2.60254500 C                 -3.11061600   -0.04946000   -4.98017800 H                 -4.14468400    0.24919000   -4.79439300 H                 -2.68409100    0.60880500   -5.74223700 H                 -3.12141700   -1.06407300   -5.39499600 H                  1.71132100    5.74071200   -0.25683000 H                  1.66787200   -5.75166700   -0.171435002-GSRu                -0.00006900   -0.32015200    0.27174500 O                  0.00016100   -2.34007200    0.85084300 O                 -0.00026500   -0.86581000   -1.72055200 N                 -2.05934800    0.01829800    0.13398000 N                 -0.00047000    1.55020100   -0.33542600 N                  2.05903300    0.01898800    0.13342300 C                  3.05706100   -0.83553800    0.40897500 C                  4.39779100   -0.48436200    0.27377300 C                  3.68171000    1.69578700   -0.44623100 H                  3.90748000    2.70056400   -0.78236000 C                  4.71325300    0.80283600   -0.16003800 H                  5.74684200    1.11101100   -0.27414600 C                  2.35627000    1.28662100   -0.29566500 C                  1.18951700    2.15053000   -0.58088700 C                  1.21119300    3.45721500   -1.07242700 H                  2.14946100    3.95882300   -1.27497500 C                 -0.00104300    4.10859600   -1.30940900 H                 -0.00126900    5.12334100   -1.69116900 C                 -1.21298800    3.45685400   -1.07195300 H                 -2.15148800    3.95817700   -1.27413800 C                 -1.19073100    2.15017300   -0.58041700 C                 -2.35712400    1.28586800   -0.29489900 C                 -3.68274500    1.69459100   -0.44510200 H                 -3.90894200    2.69932800   -0.78106200 C                 -4.71390500    0.80124700   -0.15877100 H                 -5.74762900    1.10907400   -0.27259300 C                 -4.39789000   -0.48591300    0.27475900 C                 -3.05701000   -0.83663300    0.40960500 C                  0.00042100   -2.19427200   -3.65625500 H                  0.00198100   -3.23052900   -3.99796500 H                 -0.88187800   -1.68558000   -4.05875300 C                  0.00038000   -2.07620100   -2.14815500 C                  0.00090600   -3.24406900   -1.36833800 H                  0.00136900   -4.18316100   -1.90804400 C                  0.00065000   -3.32673200    0.03888200 C                  0.00080400   -4.68906200    0.69737100 H                  0.00128700   -5.50767500   -0.02422000 H                 -0.88085600   -4.77938700    1.34066100 N                  0.00038000    0.18174100    2.22320100 C                  0.00097000    0.45968500    3.34861700 C                  0.00217100    0.80741700    4.76265000 H                 -1.01074100    0.72860600    5.16665700 H                  0.65982000    0.12828400    5.31200200 H                  0.35956000    1.83255500    4.89249400 H                  0.88098600   -1.68276700   -4.05895500 H                  0.88205100   -4.77888300    1.34129300 H                  5.16972100   -1.20890900    0.50596200 H                  2.74996400   -1.81988200    0.74497900 H                 -2.74947100   -1.82090100    0.74543100 H                 -5.16951500   -1.21076600    0.507012001-3MLCTRu                 0.25262400    0.00722900    0.44723100 C                 -0.30249300   -3.00601300    0.39145900 H                 -1.29491800   -2.68245300    0.68255000 C                  0.02411000   -4.34387700    0.29062300 H                 -0.72657800   -5.09653700    0.49895300 C                  1.33647600   -4.69490400   -0.08747200 C                  2.25763800   -3.69908800   -0.35952300 H                  3.26792700   -3.95103500   -0.65963600 C                  1.88424800   -2.34850600   -0.25427000 C                  2.72740500   -1.21457600   -0.55997700 C                  4.03550800   -1.23046300   -1.03617000 H                  4.54164600   -2.17187400   -1.21476100 C                  4.69033600   -0.01774500   -1.29165100 C                  4.03529700    1.20107000   -1.07216400 H                  4.54101800    2.13706700   -1.27837800 C                  2.72660700    1.19936900   -0.59571400 C                  1.88315400    2.34079100   -0.32458000 C                  2.25510500    3.68843400   -0.47005000 H                  3.26569900    3.93234200   -0.77588300 C                  1.33243300    4.69078800   -0.23035100 C                  0.01912600    4.34997600    0.15545400 H                 -0.73289400    5.10784200    0.33858400 C                 -0.30612800    3.01570100    0.29676500 H                 -1.29899500    2.69976900    0.59490500 N                  0.59823900   -2.02504300    0.14617500 N                  2.10596600   -0.00483400   -0.36132700 N                  0.59638400    2.02846900    0.08420700 C                 -2.08959400    0.03960000    2.44785300 H                 -1.28541100    0.05040600    3.17294400 C                 -3.41779700    0.04664700    2.83739800 C                 -4.43009000    0.03182600    1.86540300 C                 -4.03539400    0.01071900    0.52411300 H                 -4.79013500   -0.00167000   -0.25208300 C                 -2.68388300    0.00511700    0.18426000 C                 -2.17864600   -0.01702600   -1.19976100 C                 -2.99235300   -0.03142300   -2.33149600 H                 -4.06985800   -0.02536300   -2.22330900 C                 -2.42916800   -0.05163100   -3.61152700 C                 -1.03040600   -0.05147300   -3.69981100 C                 -0.26531200   -0.03639500   -2.54364500 H                  0.81706000   -0.03459600   -2.57702100 N                 -1.72003300    0.01943800    1.14976200 N                 -0.82321000   -0.02010500   -1.32037300 C                  1.80016600    0.04798500    3.25953100 H                  5.70655000   -0.02315600   -1.66808300 H                 -3.66155100    0.06358500    3.89383400 H                 -0.53309400   -0.06111700   -4.66324900 C                  2.52357600    0.06554700    4.51835600 H                  3.37802000   -0.61355000    4.45753400 H                  2.88011900    1.07957000    4.71809800 H                  1.86085200   -0.25280600    5.32722100 N                  1.22017100    0.03433500    2.25975600 C                 -5.87927500    0.03568500    2.26035600 H                 -6.53689100    0.05493400    1.38937800 H                 -6.11159500   -0.85514400    2.85356200 H                 -6.10039300    0.90628700    2.88633600 C                 -3.28927300   -0.09762600   -4.84313500 H                 -4.28982800    0.29515900   -4.64918800 H                 -2.83621400    0.46911500   -5.66047700 H                 -3.39563700   -1.13493800   -5.18163200 H                  1.61683700    5.73094700   -0.34575800 H                  1.62194400   -5.73774900   -0.171682001-3MCRu                 0.06824200   -0.02662500   -0.49051500 C                 -0.13603300    3.27196600   -0.12180500 H                 -1.18334700    3.04487700   -0.29825700 C                  0.33761000    4.58227400   -0.13636200 H                 -0.34327300    5.40587600   -0.31818700 C                  1.69898200    4.79455100    0.08139400 C                  2.53155500    3.70296700    0.32116800 H                  3.59167800    3.85699700    0.47798300 C                  1.97754500    2.41741100    0.33775900 C                  2.76420400    1.19021400    0.61747500 C                  4.05769000    1.23928300    1.14735600 H                  4.52601500    2.18840700    1.37236100 C                  4.72955100    0.05080000    1.40983500 C                  4.09835500   -1.15974000    1.14728400 H                  4.59956300   -2.09207300    1.37135200 C                  2.80351800   -1.15403900    0.61840000 C                  2.05478700   -2.40507700    0.34172400 C                  2.64191500   -3.67584000    0.35036800 H                  3.70304300   -3.80045900    0.52511900 C                  1.84162400   -4.79193200    0.11434400 C                  0.47818500   -4.61833100   -0.12358700 H                 -0.17852400   -5.46190900   -0.30255500 C                 -0.02998300   -3.32104800   -0.13251800 H                 -1.08078600   -3.12430000   -0.32371100 N                  0.66447600    2.22343500    0.10487900 N                  2.15706700    0.00744600    0.36187800 N                  0.74018700   -2.24862800    0.08874900 C                 -2.28630000    0.05758000   -2.46434700 H                 -1.48470200    0.06606800   -3.19234600 C                 -3.61486100    0.08735900   -2.85217700 C                 -4.62602400    0.07484900   -1.87921800 C                 -4.22821200    0.03345000   -0.54058700 H                 -4.98122300    0.02364500    0.23769100 C                 -2.87579700    0.00438000   -0.20089800 C                 -2.37150900   -0.03958000    1.18098400 C                 -3.18666100   -0.06364800    2.31385500 H                 -4.26440600   -0.05197300    2.20590000 C                 -2.62439700   -0.10364000    3.59194300 C                 -1.22471100   -0.12228700    3.67656500 C                 -0.46264100   -0.09783500    2.51914200 H                  0.61968300   -0.11219300    2.55476700 N                 -1.91062400    0.01685100   -1.16737500 N                 -1.01433200   -0.05742600    1.29152000 C                  1.57939500    0.02914200   -3.32508600 H                  5.72930200    0.06770600    1.82938200 H                 -3.85950300    0.11991100   -3.90822600 H                 -0.72628200   -0.15740300    4.63921300 C                  2.28729900    0.05621800   -4.59465300 H                  3.11718100    0.76571600   -4.53834200 H                  2.68004100   -0.93939700   -4.81797200 H                  1.60443900    0.36165400   -5.39186000 N                  1.01405200    0.00765400   -2.31444200 C                 -6.07666000    0.10064300   -2.27136500 H                 -6.73155000    0.13468700   -1.39852100 H                 -6.28721300    0.97103800   -2.90121800 H                 -6.32637300   -0.78983500   -2.85824700 C                 -3.47936700   -0.10397000    4.82919700 H                 -4.51115200   -0.38440400    4.60604900 H                 -3.08066400   -0.79210200    5.57983200 H                 -3.49044300    0.89637400    5.27730800 H                  2.28056500   -5.78391500    0.11556300 H                  2.11175900    5.79754500    0.063119002-3MLCTRu                -0.00039000   -0.36918300    0.22006800 O                  0.00054100   -2.35086200    0.73692600 O                 -0.00295600   -0.83670600   -1.75083700 N                 -2.03638100    0.07915000    0.19839800 N                 -0.00114200    1.55309300   -0.36384800 N                  2.03547200    0.07906200    0.19312700 C                  3.01935400   -0.78164500    0.53758000 C                  4.35686700   -0.45764000    0.40966100 C                  3.69724100    1.70850300   -0.42871600 H                  3.94361200    2.69310600   -0.80892900 C                  4.69906400    0.81695800   -0.08808500 H                  5.74014600    1.10012300   -0.19995700 C                  2.34803000    1.34033700   -0.28191000 C                  1.20556600    2.17877800   -0.57364900 C                  1.21353800    3.49842700   -1.01795500 H                  2.15261100    4.01352100   -1.18419000 C                 -0.00222700    4.15915600   -1.24102000 H                 -0.00266000    5.18793800   -1.58223900 C                 -1.21744700    3.49840100   -1.01499300 H                 -2.15693000    4.01349300   -1.17891800 C                 -1.20836800    2.17876200   -0.57071400 C                 -2.35011100    1.34030500   -0.27619200 C                 -3.69966500    1.70828800   -0.42017300 H                 -3.94696900    2.69278500   -0.80006600 C                 -4.70066300    0.81665400   -0.07731700 H                 -5.74200800    1.09967300   -0.18707800 C                 -4.35725800   -0.45783300    0.41983600 C                 -3.01942300   -0.78164000    0.54498000 C                 -0.00475100   -2.07921800   -3.74112000 H                 -0.00585400   -3.10022900   -4.12432200 H                 -0.88656600   -1.55085700   -4.11751000 C                 -0.00306700   -2.02861000   -2.23442000 C                 -0.00173600   -3.22290400   -1.49231400 H                 -0.00207200   -4.14564700   -2.05840800 C                 -0.00017800   -3.33739400   -0.09530600 C                  0.00087000   -4.69723200    0.55319400 H                 -0.00006000   -5.50870000   -0.17555300 H                 -0.87984900   -4.78872800    1.19722000 N                  0.00332500    0.06739500    2.21792200 C                  0.00825200    0.30183200    3.34962200 C                  0.01599900    0.59310200    4.77455900 H                 -1.00549300    0.55794200    5.16201400 H                  0.62629700   -0.14761800    5.29781200 H                  0.43243600    1.58940200    4.94410600 H                  0.87705100   -1.55215400   -4.11939800 H                  0.88340600   -4.78856500    1.19475800 H                  5.11486400   -1.17768000    0.69384500 H                  2.69195000   -1.74426000    0.91508900 H                 -2.69111600   -1.74418900    0.92188100 H                 -5.11454600   -1.17796400    0.705677002-3MCRu                 0.04755200   -0.53195300    0.28462700 O                  0.38152600   -2.50721300    0.71831000 O                  0.05291500   -0.93899100   -1.71753300 N                 -2.27901000   -0.01076000    0.34778400 N                 -0.20344200    1.56750800   -0.27716400 N                  2.18976400    0.50486900    0.31215500 C                  3.33819800   -0.15715500    0.50509500 C                  4.57074200    0.35216700    0.10283700 C                  3.40400400    2.28354300   -0.73625100 H                  3.40912800    3.23757700   -1.24894800 C                  4.59751800    1.59342600   -0.53538100 H                  5.53491600    2.01836100   -0.87849600 C                  2.20651200    1.71277100   -0.28727000 C                  0.88375800    2.36259200   -0.43920600 C                  0.75038400    3.72497300   -0.72474700 H                  1.62699600    4.35297900   -0.81855100 C                 -0.52096600    4.27238400   -0.85858100 H                 -0.64471700    5.32997500   -1.06368800 C                 -1.63134900    3.44792000   -0.71361600 H                 -2.62977600    3.85739400   -0.79824500 C                 -1.44497000    2.09191100   -0.42753200 C                 -2.58002700    1.15541300   -0.25928500 C                 -3.88027700    1.43145400   -0.69995400 H                 -4.10831400    2.35421100   -1.21931100 C                 -4.88101500    0.48682000   -0.48293700 H                 -5.89378000    0.68089900   -0.82002700 C                 -4.56348700   -0.70859700    0.16412500 C                 -3.24340700   -0.91669900    0.55752900 C                  0.17241300   -2.10688400   -3.75479600 H                  0.31704100   -3.10439900   -4.17231300 H                 -0.79479900   -1.71554900   -4.08700000 C                  0.22295200   -2.09756000   -2.24578900 C                  0.44392400   -3.29789100   -1.54643300 H                  0.57377100   -4.19282100   -2.14223500 C                  0.51403600   -3.44841000   -0.15476400 C                  0.76622900   -4.81165500    0.44240400 H                  0.87142900   -5.58879500   -0.31612500 H                 -0.06024900   -5.07241200    1.11183200 N                  0.02982900   -0.19209400    2.32108100 C                  0.02169300   -0.00198200    3.46367700 C                  0.01058400    0.24220500    4.89903400 H                 -0.00407000    1.31840500    5.09135200 H                 -0.87691300   -0.21340400    5.34634100 H                  0.90424000   -0.19096100    5.35620700 H                  0.94554600   -1.43807900   -4.14731200 H                  1.67673100   -4.77734700    1.04993000 H                  5.47895300   -0.21170100    0.28330900 H                  3.25243400   -1.12302800    0.99484100 H                 -2.93532700   -1.83211200    1.05462300 H                 -5.31575500   -1.46477200    0.358083001-3TSIfreq = -212.78 cm-1Ru                 0.21349500    0.00064100    0.45056600 C                 -0.24510200   -3.07093300    0.25912200 H                 -1.27592700   -2.78148300    0.43317900 C                  0.15862300   -4.39381900    0.31302900 H                 -0.56941900   -5.16964100    0.51918700 C                  1.51155900   -4.69988400    0.08677600 C                  2.40060700   -3.67504200   -0.20511000 H                  3.45014100   -3.88756400   -0.37179700 C                  1.93414900   -2.35503400   -0.27327200 C                  2.74491300   -1.19276400   -0.61125800 C                  4.01228400   -1.21293500   -1.19305800 H                  4.48834400   -2.15564900   -1.43421300 C                  4.64822100   -0.00359600   -1.48352200 C                  4.01420900    1.20727600   -1.19496200 H                  4.49181500    2.14882100   -1.43761500 C                  2.74685100    1.19014000   -0.61308600 C                  1.93842300    2.35481200   -0.27687300 C                  2.40751800    3.67401000   -0.21138600 H                  3.45740300    3.88419500   -0.37877800 C                  1.52051100    4.70116500    0.07876500 C                  0.16706600    4.39820900    0.30598700 H                 -0.55941600    5.17582900    0.51087500 C                 -0.23920300    3.07597100    0.25477900 H                 -1.27051400    2.78881400    0.42974900 N                  0.61621800   -2.06694200   -0.01804300 N                  2.14637900   -0.00072100   -0.32627500 N                  0.62015800    2.06992200   -0.02069600 C                 -2.13298400    0.00572500    2.42929200 H                 -1.33076500    0.00567300    3.15635400 C                 -3.46123600    0.00920200    2.81925800 C                 -4.47350600    0.00964800    1.84781300 C                 -4.07731100    0.00779500    0.50733300 H                 -4.83160900    0.00919700   -0.26953800 C                 -2.72559500    0.00466000    0.16602000 C                 -2.22500500    0.00272000   -1.21978600 C                 -3.04308000    0.00515500   -2.35006300 H                 -4.12051100    0.00961600   -2.24017100 C                 -2.48240000    0.00341000   -3.63066800 C                 -1.08336700    0.00394900   -3.72142300 C                 -0.31786700    0.00169500   -2.56513900 H                  0.76471200    0.00307400   -2.60170800 N                 -1.76039300    0.00350800    1.13147100 N                 -0.87155500    0.00076600   -1.34101500 C                  1.71064800   -0.00664000    3.27993500 H                  5.62561200   -0.00474600   -1.95244800 H                 -3.70399700    0.01220000    3.87612500 H                 -0.58827600    0.00793200   -4.68620500 C                  2.41960700   -0.01213000    4.54764900 H                  3.26978900   -0.69678700    4.48828800 H                  2.78197300    0.99581300    4.76693200 H                  1.74576500   -0.33672500    5.34482300 N                  1.14188300   -0.00221300    2.27259100 C                 -5.92342100    0.00277800    2.24146300 H                 -6.57875900    0.11209600    1.37527000 H                 -6.17137800   -0.93736300    2.74637400 H                 -6.13349700    0.81280800    2.94683200 C                 -3.34362400   -0.02321600   -4.86274200 H                 -4.35307400    0.33898100   -4.65569400 H                 -2.90581500    0.58207600   -5.66082600 H                 -3.42501200   -1.05029300   -5.23727100 H                  1.87091800    5.72611000    0.13089200 H                  1.85994800   -5.72540700    0.141001002-3TSIfreq = -187.92 cm-1Ru                -0.00075500   -0.43182000    0.26613900 O                 -0.00219900   -2.41947900    0.75771000 O                 -0.00369200   -0.87648800   -1.70923300 N                 -2.12433700    0.19552800    0.33068600 N                  0.00032500    1.57239500   -0.32836200 N                  2.12427800    0.19155000    0.32449800 C                  3.14414200   -0.66258300    0.54428100 C                  4.44536500   -0.37929400    0.15638600 C                  3.65780100    1.71626100   -0.73330600 H                  3.83153500    2.64818900   -1.25862500 C                  4.70473000    0.83245500   -0.50133100 H                  5.70845600    1.07620200   -0.83244400 C                  2.36837400    1.38195900   -0.29883600 C                  1.18552500    2.22588700   -0.47739100 C                  1.20819300    3.58865000   -0.77815800 H                  2.15262100    4.10959500   -0.87952000 C                  0.00188800    4.27462200   -0.92370200 H                  0.00253700    5.33751300   -1.13798400 C                 -1.20521100    3.59074400   -0.77508300 H                 -2.14900700    4.11330900   -0.87401000 C                 -1.18413200    2.22793800   -0.47433800 C                 -2.36793800    1.38606000   -0.29268400 C                 -3.65775900    1.72218800   -0.72458000 H                 -3.83109400    2.65411700   -1.25003400 C                 -4.70557900    0.84012900   -0.49003100 H                 -5.70960700    1.08525900   -0.81920500 C                 -4.44670500   -0.37173800    0.16769900 C                 -3.14513500   -0.65689500    0.55299100 C                 -0.00661500   -2.09424000   -3.71743400 H                 -0.00786500   -3.11051000   -4.11378100 H                 -0.88826000   -1.56182500   -4.08894900 C                 -0.00512000   -2.06311800   -2.20908900 C                 -0.00533600   -3.26520000   -1.48324000 H                 -0.00668800   -4.18199300   -2.05922500 C                 -0.00400100   -3.39341000   -0.08634300 C                 -0.00459400   -4.76299600    0.54565200 H                 -0.00595200   -5.56555100   -0.19324200 H                 -0.88568900   -4.86348200    1.18797200 N                  0.00382100   -0.04021700    2.31501200 C                  0.00934300    0.16991600    3.45268400 C                  0.01792700    0.43431100    4.88404300 H                 -1.00457600    0.41184400    5.26986800 H                  0.61242000   -0.32654700    5.39656100 H                  0.45259300    1.41901900    5.07476600 H                  0.87524200   -1.56335200   -4.09063400 H                  0.87748900   -4.86497800    1.18637700 H                  5.23866500   -1.08637300    0.37056100 H                  2.88457900   -1.58764700    1.04963100 H                 -2.88596700   -1.58216000    1.05817200 H                 -5.24065300   -1.07749200    0.383842001-3TSIIfreq = -95.91 cm-1Ru                 0.32153400   -0.21590400   -0.40549600 C                 -0.88845500    2.28952500   -1.84799600 H                 -1.79376300    1.69341400   -1.82552400 C                 -0.83245400    3.50974700   -2.51305000 H                 -1.71093300    3.88574600   -3.02407600 C                  0.36718100    4.22268700   -2.50270600 H                  0.44767400    5.17491100   -3.01543600 C                  1.46624100    3.70239400   -1.82233700 H                  2.40296000    4.24531600   -1.80467600 C                  1.34540600    2.47559300   -1.16731200 C                  2.41680800    1.84526300   -0.37564100 C                  3.65545100    2.42183500   -0.08882800 H                  3.91549100    3.39526400   -0.48473500 C                  4.54823100    1.73499100    0.73122600 C                  4.18385300    0.50551500    1.27671500 H                  4.85578000   -0.01354900    1.94824500 C                  2.93437200   -0.03409600    0.96538200 C                  2.38506700   -1.28293000    1.52911100 C                  3.11240200   -2.17184500    2.32348200 H                  4.15948400   -1.99498900    2.53569500 C                  2.47379400   -3.29910900    2.83814300 H                  3.02514100   -3.99978800    3.45576700 C                  1.12499100   -3.51324000    2.55332600 H                  0.59345200   -4.37371500    2.94259100 C                  0.46171200   -2.59194200    1.74663500 H                 -0.58462200   -2.70957500    1.48534500 N                  0.17182200    1.77988800   -1.19805500 N                  2.09531200    0.62475200    0.12571100 N                  1.07520900   -1.51177700    1.24326400 C                 -1.75865400   -1.89312600   -1.88649800 H                 -0.86184100   -2.16686700   -2.42856400 C                 -2.99026600   -2.43062100   -2.22297100 C                 -4.12919000   -2.06636200   -1.49219200 C                 -3.94871100   -1.15847300   -0.44700300 H                 -4.80195800   -0.86665900    0.15255600 C                 -2.68639900   -0.63468600   -0.15645600 C                 -2.45806300    0.34041600    0.93517400 C                 -3.49290300    0.96971000    1.63802500 H                 -4.52892400    0.77241200    1.39013300 C                 -3.19684500    1.87420200    2.66083400 C                 -1.84315100    2.11672100    2.93989100 C                 -0.86836800    1.46904500    2.19283100 H                  0.18905800    1.64062600    2.37171800 N                 -1.59123200   -1.01198200   -0.87657700 N                 -1.16523200    0.60309900    1.21437600 C                  2.38112900   -2.16758400   -2.26962200 H                  5.51443300    2.16775400    0.96420500 H                 -3.05592000   -3.13418500   -3.04588400 H                 -1.55283800    2.80572700    3.72625900 C                  3.48632100   -2.85250300   -2.92140000 H                  3.88160800   -2.22804100   -3.72684800 H                  4.27787500   -3.04355200   -2.19195100 H                  3.14073100   -3.80244000   -3.33732000 N                  1.50156300   -1.62247100   -1.74531500 C                 -5.48477300   -2.62093900   -1.83219400 H                 -6.21154100   -2.42412700   -1.04110400 H                 -5.85619600   -2.16365900   -2.75650800 H                 -5.43266700   -3.70012900   -2.00328000 C                 -4.28235400    2.57550700    3.43216200 H                 -5.27546500    2.22694700    3.14058900 H                 -4.15762300    2.41158700    4.50743000 H                 -4.23320400    3.65662300    3.262964002-3TSIIfreq = -112.99 cm-1Ru                 0.02255300   -0.43497800    0.21489300 O                  0.23097800   -2.31186500    1.01348000 O                  0.16343800   -1.23057300   -1.70824800 N                 -2.13510800   -0.02135200    0.32308300 N                 -0.16938000    1.42741400   -0.57432700 N                  2.04751300    0.40815000    0.33548100 C                  3.13331400   -0.25029700    0.78000000 C                  4.42580100    0.16101600    0.47730400 C                  3.47970200    1.97957100   -0.78707300 H                  3.59561100    2.85115500   -1.42003600 C                  4.59925400    1.29394600   -0.32455900 H                  5.59413300    1.63626100   -0.58825700 C                  2.20571700    1.52149000   -0.43570000 C                  0.94661100    2.16190400   -0.84061900 C                  0.83971500    3.42596500   -1.42312300 H                  1.72773500    4.01301800   -1.62192100 C                 -0.42257900    3.93530600   -1.72253800 H                 -0.52178200    4.91944700   -2.16631600 C                 -1.55754600    3.18322500   -1.42492900 H                 -2.54506700    3.58020300   -1.62474300 C                 -1.40941400    1.92310400   -0.84301400 C                 -2.51447300    1.04249600   -0.44056800 C                 -3.85558400    1.24063400   -0.78516800 H                 -4.14413700    2.07492500   -1.41324000 C                 -4.81513100    0.34513500   -0.32193500 H                 -5.85935800    0.48475700   -0.57992200 C                 -4.41705600   -0.73398900    0.47413600 C                 -3.06732200   -0.88287800    0.76934600 C                  0.40408600   -2.78626900   -3.45879700 H                  0.54009400   -3.85015700   -3.65966700 H                 -0.51336400   -2.44257700   -3.94789800 C                  0.32661700   -2.47783900   -1.98172500 C                  0.43304300   -3.52097900   -1.04820800 H                  0.56839800   -4.51876600   -1.44676200 C                  0.38305500   -3.40329900    0.35202700 C                  0.51655100   -4.63800000    1.21134600 H                  0.64481500   -5.54740500    0.62245200 H                 -0.37471000   -4.73949500    1.83935900 N                 -0.06218000    0.30825600    2.50284200 C                 -0.08102900    1.03373200    3.40760700 C                 -0.10174700    1.95021300    4.54026700 H                 -0.10190000    2.98229600    4.17947300 H                 -0.99987600    1.78002400    5.13944900 H                  0.78134600    1.78874200    5.16375200 H                  1.23603500   -2.22805500   -3.90079300 H                  1.37427100   -4.52024800    1.88182600 H                  5.27379400   -0.39255600    0.86395800 H                  2.93632000   -1.12552800    1.39072500 H                 -2.69863200   -1.70414000    1.37546900 H                 -5.13682400   -1.44573400    0.861930001-[3Rup:ACN]Ru                 0.28231800   -0.01604700   -0.33183500 C                 -0.54683400    2.71865500   -1.57479400 H                 -1.48246700    2.20416300   -1.76001500 C                 -0.34639600    4.03884700   -1.96198300 H                 -1.13973500    4.57789400   -2.46576500 C                  0.88320900    4.63705100   -1.68664500 H                  1.07275100    5.66551000   -1.97361300 C                  1.87215500    3.90325000   -1.03334900 H                  2.82944100    4.35722900   -0.81012200 C                  1.61785600    2.58113400   -0.67112300 C                  2.58324800    1.71948200    0.03929400 C                  3.86259000    2.08014100    0.46299600 H                  4.25296800    3.07056600    0.26609400 C                  4.63420200    1.14476100    1.15435000 C                  4.12416900   -0.12473200    1.42789800 H                  4.71775700   -0.84434000    1.97715800 C                  2.83946900   -0.44524700    0.98593800 C                  2.13352800   -1.72401000    1.19007100 C                  2.67717700   -2.81772200    1.86378400 H                  3.67646300   -2.76417700    2.27704400 C                  1.92429800   -3.98298300    1.99802100 H                  2.34012500   -4.83959300    2.51689400 C                  0.63677100   -4.03319300    1.46314300 H                  0.02135200   -4.92072100    1.54865600 C                  0.14208200   -2.91128600    0.80811100 H                 -0.85191500   -2.89274200    0.37688500 N                  0.40707500    2.00253000   -0.95290200 N                  2.11535600    0.47182800    0.29581000 N                  0.86797500   -1.78741400    0.66491500 C                 -1.78407400   -1.09493900   -2.31712600 H                 -0.86890100   -1.24743400   -2.87943400 C                 -3.02296000   -1.44125300   -2.83298300 C                 -4.17869000   -1.23400600   -2.06679600 C                 -4.01195900   -0.67790500   -0.79650600 H                 -4.88320000   -0.50312400   -0.17760300 C                 -2.74037800   -0.34792100   -0.32210600 C                 -2.49733700    0.23949800    1.01517400 C                 -3.51152400    0.52190500    1.93501000 H                 -4.54659000    0.31477300    1.69237100 C                 -3.20034700    1.07651000    3.17988900 C                 -1.84951900    1.33445300    3.45518300 C                 -0.88959800    1.03065800    2.49968300 H                  0.16502300    1.21675200    2.67701200 N                 -1.63469900   -0.55691200   -1.08747600 N                 -1.20244500    0.49688800    1.30932500 C                  2.46689100   -1.90589400   -2.70588000 H                  5.63075800    1.40905400    1.48876300 H                 -3.08384700   -1.86964100   -3.82768500 H                 -1.54741200    1.76850800    4.40239200 C                  3.69089600   -2.22542400   -1.97898700 H                  3.47913800   -2.95810100   -1.19609900 H                  4.43444100   -2.64216700   -2.66292900 H                  4.09919900   -1.32053000   -1.52143000 N                  1.49028600   -1.65018800   -3.27997000 C                 -5.53649100   -1.60911100   -2.59331900 H                 -5.70111300   -1.17116600   -3.58278200 H                 -5.61167700   -2.69639400   -2.70518200 H                 -6.33505900   -1.27717700   -1.92657400 C                 -4.26898500    1.36874400    4.19753400 H                 -4.10545600    2.34334700    4.66635500 H                 -5.26573500    1.35513200    3.75123300 H                 -4.24380600    0.61653900    4.994315002-[3Rup:ACN] Ru                 0.20701700    0.40236400    0.17248700 O                  0.97768900    2.08950700    1.06088000 O                  0.66287000    1.26366300   -1.76128300 N                  1.80257500   -0.97707300    0.34367300 N                 -0.56408700   -1.25105000   -0.63177900 N                 -1.86793600    0.77489700    0.27255800 C                 -2.44325600    1.87133400    0.80279300 C                 -3.79693900    2.14223000    0.64441500 C                 -3.99187600    0.11284500   -0.63898600 H                 -4.58473600   -0.58906500   -1.21260600 C                 -4.58092300    1.24919400   -0.09038600 H                 -5.63980300    1.43446900   -0.23358800 C                 -2.62798400   -0.11193600   -0.44406800 C                 -1.89360100   -1.29105300   -0.92237800 C                 -2.43956500   -2.41133200   -1.54929100 H                 -3.49685500   -2.45099800   -1.78040600 C                 -1.61061600   -3.49165100   -1.85282000 H                 -2.02034400   -4.36939000   -2.33907400 C                 -0.26114900   -3.45605900   -1.49916400 H                  0.37809900   -4.30860600   -1.69247600 C                  0.24742600   -2.31773700   -0.87294800 C                  1.61089400   -2.13929200   -0.35451200 C                  2.64797500   -3.06192600   -0.50146500 H                  2.49238500   -3.97428800   -1.06405700 C                  3.88553200   -2.79484000    0.07873100 H                  4.69881000   -3.50441600   -0.02737800 C                  4.06602000   -1.60992200    0.79646000 C                  3.00033400   -0.72466600    0.90499300 C                  1.43682300    2.79703000   -3.37513700 H                  1.89870400    3.77977300   -3.48454100 H                  2.07527700    2.04543000   -3.85149500 C                  1.21101500    2.41050900   -1.93026300 C                  1.60408600    3.30297200   -0.91358500 H                  2.05224700    4.23490200   -1.23544200 C                  1.47811500    3.11939900    0.47337700 C                  1.95756900    4.19711500    1.41625900 H                  2.37113500    5.06074300    0.89344200 H                  2.72040900    3.78044200    2.08234800 N                 -0.21297500   -0.54405500    3.78045600 C                 -0.82947300   -1.35423600    3.22125100 C                 -1.60042300   -2.36272300    2.50099300 H                 -0.95634300   -2.88005400    1.78574200 H                 -2.01385800   -3.09321500    3.20037900 H                 -2.42081500   -1.88438300    1.96077600 H                  0.47772000    2.78899200   -3.90347200 H                  1.12175100    4.52266000    2.04430500 H                 -4.22215200    3.03419500    1.08918600 H                 -1.78181600    2.52970400    1.35495900 H                  3.08093300    0.21421400    1.44149500 H                  5.01310800   -1.37107300    1.26571300 1-[Rup:ACN]Ru                 0.19625400   -0.09117600   -0.40612100 C                 -0.75149600    2.40469100   -1.99220600 H                 -1.65210500    1.81965600   -2.13772300 C                 -0.62581700    3.69233600   -2.50786300 H                 -1.44470500    4.12700000   -3.06888700 C                  0.55857500    4.39129400   -2.28615400 H                  0.69021800    5.39580900   -2.67273600 C                  1.57938300    3.78355500   -1.55537000 H                  2.50609700    4.31242400   -1.36951800 C                  1.39719100    2.49302000   -1.06201600 C                  2.41631500    1.76817200   -0.26984100 C                  3.69307300    2.21858300    0.07052200 H                  4.04487600    3.19084200   -0.25064600 C                  4.51673800    1.38794700    0.83272000 C                  4.07342800    0.12929100    1.24407700 H                  4.71913000   -0.51276200    1.82964300 C                  2.78966600   -0.28090900    0.88009100 C                  2.14266600   -1.57324000    1.20150400 C                  2.75168300   -2.57359500    1.95742700 H                  3.74841200   -2.42676000    2.35453600 C                  2.06918400   -3.76634400    2.19702400 H                  2.53469500   -4.55048800    2.78366800 C                  0.78846300   -3.93226900    1.67434900 H                  0.22302000   -4.84225800    1.83702400 C                  0.22982300   -2.89882600    0.92645800 H                 -0.76176000   -2.97888600    0.49657200 N                  0.22753100    1.81152000   -1.28864300 N                  2.00919300    0.54602000    0.14522000 N                  0.88133800   -1.74795900    0.68905300 C                 -2.07125400   -1.41155100   -2.01188100 H                 -1.25220500   -1.69052300   -2.66586600 C                 -3.38608500   -1.76070100   -2.28210300 C                 -4.40790400   -1.38577300   -1.39658700 C                 -4.03757100   -0.66451200   -0.25879900 H                 -4.79763800   -0.35689200    0.44922900 C                 -2.69843400   -0.34083100   -0.03495300 C                 -2.20070500    0.40407600    1.13245300 C                 -3.01082900    0.87881200    2.16405600 H                 -4.07960400    0.70744600    2.12447900 C                 -2.46537800    1.57194100    3.24660400 C                 -1.07699200    1.76520400    3.24528000 C                 -0.30943700    1.27833900    2.20043600 H                  0.76258300    1.41910900    2.18064200 N                 -1.72790300   -0.71130800   -0.91271000 N                 -0.84889600    0.61129800    1.15769200 C                  2.17817400   -2.40599500   -2.55457900 H                  5.51144300    1.72193400    1.10536800 H                 -3.61164800   -2.32309200   -3.18187300 H                 -0.58696200    2.29646900    4.05380200 C                  3.50134300   -2.45747000   -1.94175800 H                  3.46181200   -3.04994000   -1.02417500 H                  4.21607700   -2.91476000   -2.63032500 H                  3.83907000   -1.44624700   -1.70024600 N                  1.12042300   -2.36620700   -3.03330400 C                 -5.83995300   -1.76448500   -1.65885300 H                 -6.11672300   -1.54566300   -2.69451400 H                 -5.98001800   -2.84061200   -1.50612700 H                 -6.52538500   -1.23531300   -0.99330100 C                 -3.32197700    2.07270900    4.37616900 H                 -3.02637800    3.08338500    4.67172800 H                 -4.38014900    2.07963800    4.10602000 H                 -3.20037300    1.42787200    5.254139002-[Rup:ACN]Ru                 0.20697400    0.41165500    0.35592000 O                  0.98403800    2.13719000    1.26666600 O                  0.53208300    1.14300700   -1.49782100 N                  1.94991000   -0.76409200    0.20007900 N                 -0.52935300   -1.21378900   -0.49780900 N                 -1.82558800    0.95106800    0.18822800 C                 -2.41857900    2.08573400    0.59678900 C                 -3.78088700    2.31978500    0.42851400 C                 -3.95179200    0.15950000   -0.61390900 H                 -4.54122400   -0.61410600   -1.09075200 C                 -4.55862200    1.33964700   -0.18636600 H                 -5.62317700    1.48720300   -0.33156800 C                 -2.58207300   -0.01904300   -0.42132500 C                 -1.84698700   -1.23759100   -0.82874100 C                 -2.37553800   -2.35395400   -1.47913300 H                 -3.42401800   -2.39512600   -1.74663000 C                 -1.53012000   -3.42312000   -1.78089200 H                 -1.92655500   -4.29914200   -2.28149600 C                 -0.17422000   -3.36734400   -1.44944500 H                  0.48130200   -4.19313800   -1.69596800 C                  0.31795800   -2.23101300   -0.80442700 C                  1.71803600   -1.98037400   -0.39304100 C                  2.75616000   -2.89310800   -0.57870500 H                  2.56339900   -3.85208900   -1.04398900 C                  4.04525200   -2.56323100   -0.16202900 H                  4.85890800   -3.26614800   -0.30387700 C                  4.27056500   -1.32382200    0.43510800 C                  3.19550600   -0.45403400    0.59807000 C                  1.15110900    2.61585400   -3.20474100 H                  1.58136600    3.60053700   -3.39221600 H                  1.77377100    1.85403800   -3.68476900 C                  1.03361000    2.31100400   -1.72907300 C                  1.45229800    3.26153700   -0.79000800 H                  1.84912600    4.18585900   -1.19090600 C                  1.41857700    3.14777800    0.61787300 C                  1.92903200    4.28821400    1.46741700 H                  2.27567200    5.13432200    0.87216100 H                  2.75230400    3.93036300    2.09468200 N                 -0.32388200   -1.31814400    3.45705100 C                 -0.99048000   -2.07225400    2.87655200 C                 -1.82945100   -3.02127300    2.15233100 H                 -1.27463700   -3.44262900    1.31025200 H                 -2.13532000   -3.83296600    2.81719700 H                 -2.72112700   -2.51511400    1.77492800 H                  0.15931200    2.56370300   -3.66554600 H                  1.13172900    4.62367200    2.13873200 H                 -4.21292900    3.25149200    0.77480800 H                 -1.76534300    2.81117200    1.06912700 H                  3.31044800    0.52240200    1.05572900 H                  5.25744600   -1.02735300    0.771113001-1RupRu                 0.26253700    0.00114900   -0.74603200 C                 -0.22540100    3.07086400   -0.80636000 H                 -1.24776200    2.78064500   -1.01893700 C                  0.14594900    4.40836900   -0.69284500 H                 -0.60014800    5.18406900   -0.81897600 C                  1.47679200    4.71390300   -0.41714100 C                  2.39426500    3.67485900   -0.26225200 H                  3.43263900    3.89243600   -0.04462800 C                  1.96535100    2.35461700   -0.38545000 C                  2.85921400    1.18498400   -0.22757800 C                  4.23066500    1.20574500    0.03188000 H                  4.76484500    2.14204300    0.13264200 C                  4.90723800   -0.00867300    0.16128000 C                  4.22564800   -1.22021400    0.03120500 H                  4.75602400   -2.15874700    0.13131900 C                  2.85427800   -1.19363800   -0.22819300 C                  1.95566700   -2.35957100   -0.38668600 C                  2.37918100   -3.68153700   -0.26332300 H                  3.41651900   -3.90332700   -0.04506000 C                  1.45761200   -4.71687700   -0.41872000 C                  0.12817200   -4.40597200   -0.69514000 H                 -0.62097900   -5.17865400   -0.82169000 C                 -0.23771100   -3.06696900   -0.80877400 H                 -1.25880100   -2.77273200   -1.02190300 N                  0.65260600    2.06436000   -0.66017100 N                  2.22147100   -0.00297900   -0.34619000 N                  0.64423500   -2.06401400   -0.66202300 C                 -2.39001100    0.00448300   -2.30082600 H                 -1.72505700    0.00519400   -3.15814000 C                 -3.76885800    0.00079400   -2.44657100 C                 -4.59131500   -0.00221500   -1.30964900 C                 -3.96186800   -0.00332500   -0.06206000 H                 -4.56335900   -0.00896200    0.83897300 C                 -2.56870400    0.00129900    0.02432100 C                 -1.80374600    0.00050200    1.28063400 C                 -2.38201200    0.00047400    2.54994300 H                 -3.46102400    0.00084700    2.64377600 C                 -1.59140300    0.00027800    3.70043200 C                 -0.20111100    0.00007000    3.51830100 C                  0.33168900    0.00040900    2.24002600 H                  1.40109200    0.00027200    2.07854300 N                 -1.79435800    0.00458200   -1.09308200 N                 -0.44363500    0.00074100    1.13428400 H                  5.97214500   -0.01093800    0.36449200 H                 -4.19857300   -0.00157300   -3.44244800 H                  0.47224900   -0.00030400    4.36847600 C                 -6.09002200    0.01425000   -1.43738300 H                 -6.57807100   -0.23245600   -0.49203300 H                 -6.43087400    1.00917700   -1.74605700 H                 -6.42378100   -0.69416500   -2.20125700 C                 -2.19568600    0.00191700    5.07704900 H                 -3.28673200   -0.01404200    5.03877100 H                 -1.85479100   -0.86949400    5.64570900 H                 -1.88094700    0.89262300    5.63100900 H                  1.77745700   -5.74854500   -0.32250000 H                  1.80085600    5.74427000   -0.321113002-1RupRu                -0.00002600    0.33721300   -0.59498600 O                  0.00053900    2.38247400   -1.04625100 O                  0.00001300    0.69218400    1.39090300 N                 -2.07357900   -0.03319900   -0.50184200 N                 -0.00073900   -1.59211600   -0.16301600 N                  2.07338600   -0.03491700   -0.50178300 C                  3.08129800    0.83083100   -0.70393700 C                  4.41877800    0.46013100   -0.59332500 C                  3.68374000   -1.76411300   -0.04937200 H                  3.90236900   -2.79318200    0.20858700 C                  4.72304700   -0.85923900   -0.26110700 H                  5.75375700   -1.18336700   -0.16707100 C                  2.36180900   -1.33577800   -0.17147700 C                  1.18913700   -2.21379900    0.04233200 C                  1.20965700   -3.55238100    0.43746600 H                  2.14748000   -4.06693900    0.60634500 C                 -0.00184400   -4.21957600    0.62692100 H                 -0.00227500   -5.25860300    0.93649500 C                 -1.21279500   -3.55140300    0.43733400 H                 -2.15105400   -4.06521000    0.60609300 C                 -1.19115500   -2.21283000    0.04225000 C                 -2.36310100   -1.33381100   -0.17148200 C                 -3.68538900   -1.76095500   -0.04915500 H                 -3.90488700   -2.78982900    0.20884700 C                 -4.72392000   -0.85514900   -0.26071600 H                 -5.75490300   -1.17835000   -0.16649500 C                 -4.41852900    0.46394900   -0.59298200 C                 -3.08074200    0.83346800   -0.70383700 C                  0.00142000    1.85863700    3.41721400 H                  0.00163900    2.86246200    3.84416300 H                 -0.88044400    1.31669200    3.77379800 C                  0.00107300    1.87727200    1.90589300 C                  0.00189400    3.10339900    1.22984500 H                  0.00281300    3.99125000    1.84969200 C                  0.00157300    3.30864800   -0.16766600 C                  0.00288800    4.71675100   -0.71548900 H                  0.00116100    5.47468500    0.06930700 H                 -0.87705300    4.85710800   -1.35200000 H                  0.88334900    1.31651100    3.77338200 H                  0.88594300    4.85702700   -1.34774800 H                  5.19642800    1.19537300   -0.76461000 H                  2.78510000    1.84241600   -0.95913500 H                 -2.78363300    1.84476600   -0.95911100 H                 -5.19556600    1.19987200   -0.764122001-WATRu                 0.26088000    0.00531000   -0.64605400 C                 -0.22725500    3.06712900   -0.73991600 H                 -1.23886500    2.77441100   -0.99614600 C                  0.13852600    4.40480400   -0.61783400 H                 -0.60087900    5.17963600   -0.78305100 C                  1.45579500    4.71292900   -0.28283100 C                  2.36490200    3.67579700   -0.07955500 H                  3.39246900    3.89516900    0.18327200 C                  1.94244100    2.35374400   -0.21399700 C                  2.82590500    1.18441500   -0.01287000 C                  4.18375900    1.20221500    0.31105400 H                  4.71282500    2.13810400    0.43969100 C                  4.85371900   -0.01267300    0.46666400 C                  4.17737400   -1.22228400    0.29841100 H                  4.70162500   -2.16219200    0.41702200 C                  2.81952000   -1.19403500   -0.02506100 C                  1.93039400   -2.35688300   -0.23910900 C                  2.34594300   -3.68232300   -0.11667300 H                  3.37159300   -3.90959900    0.14688100 C                  1.43207800   -4.71266900   -0.33259400 C                  0.11737300   -4.39443300   -0.66820700 H                 -0.62551000   -5.16381000   -0.84302700 C                 -0.24126600   -3.05373000   -0.77784200 H                 -1.25073500   -2.75348900   -1.03378800 N                  0.64309100    2.06082900   -0.54684200 N                  2.19086700   -0.00238100   -0.16535300 N                  0.63360400   -2.05405200   -0.57220800 C                 -2.36696800    0.01393700   -2.25093900 H                 -1.68340500    0.01512200   -3.09199200 C                 -3.74310400    0.01265200   -2.42339200 C                 -4.58797500    0.00739500   -1.30415200 C                 -3.98141900   -0.00080600   -0.04541200 H                 -4.60100500   -0.01035600    0.84316000 C                 -2.59035800    0.00155300    0.07072900 C                 -1.86335400   -0.00787500    1.35276900 C                 -2.48594900   -0.01751100    2.60227700 H                 -3.56729000   -0.01748200    2.66387200 C                 -1.73311700   -0.02760600    3.77805400 C                 -0.33776300   -0.02676100    3.64261500 C                  0.23378800   -0.01626600    2.38073900 H                  1.30877900   -0.01522600    2.25434100 N                 -1.79227300    0.00964200   -1.03239900 N                 -0.50216300   -0.00744000    1.25017100 H                  5.90819400   -0.01676500    0.71826700 H                 -4.15275000    0.01366500   -3.42779300 H                  0.30657700   -0.03454700    4.51509200 C                 -6.08449500    0.03266900   -1.45625500 H                 -6.58748800   -0.28769800   -0.54099100 H                 -6.42195600    1.04973000   -1.68757300 H                 -6.40594400   -0.61177800   -2.27932900 C                 -2.38146300   -0.03476000    5.13494600 H                 -3.46975000   -0.08605400    5.06243300 H                 -2.03096700   -0.88893400    5.72338400 H                 -2.11415900    0.87096100    5.68992900 H                  1.74538800   -5.74651000   -0.23811000 H                  1.77475100    5.74415600   -0.17913900 O                  0.76547900    0.01877700   -2.77826200 H                  1.35211500   -0.69841000   -3.06313600 H                  1.17975300    0.84010500   -3.084134002-WATRu                 0.00298000    0.31403100    0.44108100 O                  0.00542300    2.33644300    0.94343600 O                  0.00627200    0.71320900   -1.56225400 N                  2.05514200   -0.04060700    0.36514600 N                  0.00025900   -1.59754700    0.00936800 N                 -2.05163500   -0.03755000    0.36452900 C                 -3.04922300    0.83562300    0.58715300 C                 -4.39069400    0.47707500    0.49090500 C                 -3.68284000   -1.75083100   -0.07582800 H                 -3.91267600   -2.77712100   -0.33580700 C                 -4.71102500   -0.83784700    0.15282500 H                 -5.74583200   -1.15164200    0.06917100 C                 -2.35528100   -1.33508100    0.03349100 C                 -1.19099600   -2.22052000   -0.18265100 C                 -1.21297400   -3.56714600   -0.54932900 H                 -2.15181900   -4.08402000   -0.70626600 C                 -0.00143800   -4.24089800   -0.71931300 H                 -0.00199700   -5.28685900   -1.00457300 C                  1.21062900   -3.57246000   -0.53472900 H                  2.14890600   -4.09343000   -0.68121200 C                  1.19080400   -2.22528500   -0.16871000 C                  2.35594500   -1.34252500    0.05088200 C                  3.68245500   -1.76512100   -0.04696400 H                  3.90909400   -2.79556100   -0.29305000 C                  4.71342300   -0.85377300    0.17439700 H                  5.74738200   -1.17264000    0.09986700 C                  4.39603900    0.46684800    0.49227300 C                  3.05539000    0.83138200    0.57849300 C                  0.00463300    1.95127500   -3.55292800 H                  0.00803100    2.97019600   -3.94294700 H                  0.88351300    1.41945300   -3.93199400 C                  0.00609200    1.90596000   -2.04099000 C                  0.00645900    3.11263000   -1.31924600 H                  0.00691000    4.01982200   -1.91089300 C                  0.00677300    3.28111000    0.08003700 C                  0.01061900    4.67666500    0.66094800 H                 -0.00351100    5.45304300   -0.10564200 H                  0.90170700    4.80625100    1.28444900 H                 -0.87950900    1.42650600   -3.92976000 H                 -0.86112800    4.79973100    1.31217000 H                 -5.16010800    1.21746000    0.67704200 H                 -2.73837700    1.84182500    0.84655400 H                  2.74895900    1.84265800    0.82283900 H                  5.16714100    1.20730200    0.67123100 O                  0.02068100    0.04110900    2.60582300 H                 -0.79783000   -0.35705300    2.93925000 H                  0.04592400    0.93807100    2.978329003-GSRu                 0.26377800    0.00471600    0.43920100 C                 -0.22808400   -3.05636300    0.62228700 H                 -1.19082600   -2.75442600    1.01805400 C                  0.11299800   -4.39808700    0.47096300 H                 -0.59734400   -5.16681600    0.75174800 C                  1.36901700   -4.71744900   -0.04091400 C                  2.24321600   -3.68782900   -0.38628300 H                  3.22335700   -3.91610200   -0.78647100 C                  1.84658800   -2.36209600   -0.21404900 C                  2.69761300   -1.20108400   -0.55697100 C                  3.99112100   -1.23332200   -1.08203000 H                  4.48928800   -2.17436500   -1.27840500 C                  4.63678200   -0.02506300   -1.35085000 C                  4.00039100    1.19188100   -1.09969900 H                  4.50577500    2.12610300   -1.30964600 C                  2.70670400    1.17721700   -0.57433800 C                  1.86456300    2.34960100   -0.24867200 C                  2.27115700    3.66959500   -0.44077100 H                  3.25296600    3.88450500   -0.84428300 C                  1.40473300    4.71081900   -0.11093700 C                  0.14634300    4.40856900    0.40549100 H                 -0.55822400    5.18669900    0.67470000 C                 -0.20484600    3.07187500    0.57681400 H                 -1.16988500    2.78319200    0.97683800 N                  0.60866300   -2.05979500    0.29156600 N                  2.10391100   -0.00788400   -0.32531400 N                  0.62439500    2.06417300    0.26124800 C                 -2.17574900    0.02590800    2.34270700 H                 -1.40710700    0.03082400    3.10540500 C                 -3.52095600    0.03006600    2.67614100 C                 -4.49143400    0.02314600    1.66389900 C                 -4.03426200    0.01282800    0.34473500 H                 -4.75255300    0.00728400   -0.46589400 C                 -2.66576000    0.00942200    0.06547600 C                 -2.10673000   -0.00170800   -1.29919300 C                 -2.88562700   -0.00640900   -2.45758100 H                 -3.96597700   -0.00054200   -2.38110100 C                 -2.28925700   -0.01730500   -3.72081700 C                 -0.88876800   -0.01794800   -3.76564500 C                 -0.16166200   -0.01268900   -2.58561800 H                  0.92084900   -0.01234700   -2.59875900 N                 -1.74180100    0.01555700    1.06598100 N                 -0.74568000   -0.00534500   -1.37143800 C                  1.54523500    0.01764800    3.36767600 H                  5.64123800   -0.03186900   -1.75867500 H                 -3.80857400    0.03856200    3.72206200 H                 -0.36099600   -0.02097900   -4.71312500 C                  2.13554500    0.02073700    4.69741200 H                  2.93438800   -0.72381100    4.74865900 H                  2.54997100    1.00840900    4.91611500 H                  1.37099800   -0.22098700    5.44067200 N                  1.06459500    0.01430400    2.31410000 C                 -5.95861800    0.02496900    1.99508400 H                 -6.57637700    0.03681000    1.09473600 H                 -6.21916800   -0.86218100    2.58203700 H                 -6.21345400    0.89967300    2.60254500 C                 -3.11061600   -0.04946000   -4.98017800 H                 -4.14468400    0.24919000   -4.79439300 H                 -2.68409100    0.60880500   -5.74223700 H                 -3.12141700   -1.06407300   -5.39499600 H                  1.71132100    5.74071200   -0.25683000 H                  1.66787200   -5.75166700   -0.171435004-GSRu                -0.00006900   -0.32015200    0.27174500 O                  0.00016100   -2.34007200    0.85084300 O                 -0.00026500   -0.86581000   -1.72055200 N                 -2.05934800    0.01829800    0.13398000 N                 -0.00047000    1.55020100   -0.33542600 N                  2.05903300    0.01898800    0.13342300 C                  3.05706100   -0.83553800    0.40897500 C                  4.39779100   -0.48436200    0.27377300 C                  3.68171000    1.69578700   -0.44623100 H                  3.90748000    2.70056400   -0.78236000 C                  4.71325300    0.80283600   -0.16003800 H                  5.74684200    1.11101100   -0.27414600 C                  2.35627000    1.28662100   -0.29566500 C                  1.18951700    2.15053000   -0.58088700 C                  1.21119300    3.45721500   -1.07242700 H                  2.14946100    3.95882300   -1.27497500 C                 -0.00104300    4.10859600   -1.30940900 H                 -0.00126900    5.12334100   -1.69116900 C                 -1.21298800    3.45685400   -1.07195300 H                 -2.15148800    3.95817700   -1.27413800 C                 -1.19073100    2.15017300   -0.58041700 C                 -2.35712400    1.28586800   -0.29489900 C                 -3.68274500    1.69459100   -0.44510200 H                 -3.90894200    2.69932800   -0.78106200 C                 -4.71390500    0.80124700   -0.15877100 H                 -5.74762900    1.10907400   -0.27259300 C                 -4.39789000   -0.48591300    0.27475900 C                 -3.05701000   -0.83663300    0.40960500 C                  0.00042100   -2.19427200   -3.65625500 H                  0.00198100   -3.23052900   -3.99796500 H                 -0.88187800   -1.68558000   -4.05875300 C                  0.00038000   -2.07620100   -2.14815500 C                  0.00090600   -3.24406900   -1.36833800 H                  0.00136900   -4.18316100   -1.90804400 C                  0.00065000   -3.32673200    0.03888200 C                  0.00080400   -4.68906200    0.69737100 H                  0.00128700   -5.50767500   -0.02422000 H                 -0.88085600   -4.77938700    1.34066100 N                  0.00038000    0.18174100    2.22320100 C                  0.00097000    0.45968500    3.34861700 C                  0.00217100    0.80741700    4.76265000 H                 -1.01074100    0.72860600    5.16665700 H                  0.65982000    0.12828400    5.31200200 H                  0.35956000    1.83255500    4.89249400 H                  0.88098600   -1.68276700   -4.05895500 H                  0.88205100   -4.77888300    1.34129300 H                  5.16972100   -1.20890900    0.50596200 H                  2.74996400   -1.81988200    0.74497900 H                 -2.74947100   -1.82090100    0.74543100 H                 -5.16951500   -1.21076600    0.507012003-3MLCTRu                -0.46644100   -0.71012700    0.03746000 O                  1.54011400   -1.07305500    0.06542100 O                 -0.14376600    1.27817000   -0.08018000 N                 -0.87383500   -0.84151800   -1.99922000 N                 -2.42111700   -0.25677500   -0.02558000 N                 -0.94687200   -0.57583200    2.06176700 C                 -0.08383800   -0.80151800    3.07742300 C                 -0.43431500   -0.59605900    4.39848700 C                 -2.63239400    0.07309900    3.65589100 H                 -3.64035000    0.41332500    3.86321900 C                 -1.73925300   -0.14663000    4.68962600 H                 -2.04427500    0.02177900    5.71683600 C                 -2.23581200   -0.14628900    2.32475100 C                 -3.06942100    0.02437300    1.15439300 C                 -4.40206100    0.42596700    1.11202600 H                 -4.93614900    0.64612300    2.02914700 C                 -5.04910900    0.54249500   -0.12585600 H                 -6.08734600    0.85129900   -0.16484900 C                 -4.35974000    0.26153700   -1.31352900 H                 -4.86077600    0.35429400   -2.27023200 C                 -3.02710800   -0.13782300   -1.25449200 C                 -2.15300600   -0.45920500   -2.36198900 C                 -2.50323400   -0.42566000   -3.72338300 H                 -3.50304400   -0.12070200   -4.00981000 C                 -1.57527100   -0.78275200   -4.68571700 H                 -1.84452600   -0.75811300   -5.73617000 C                 -0.28192300   -1.18414900   -4.29105100 C                  0.02348500   -1.20345900   -2.94329100 C                  1.00710600    1.85956200   -0.10183100 C                  2.25385200    1.20938100   -0.03528800 H                  3.12969800    1.84061300   -0.05326100 C                  2.47189800   -0.17443500    0.04845400 N                 -0.75896100   -2.73045500    0.15779200 C                 -0.90791800   -3.87462300    0.22409800 C                 -1.09266400   -5.31485700    0.30679900 H                 -1.95594300   -5.60999100   -0.29527300 H                 -0.20001800   -5.82258300   -0.06764900 H                 -1.26252800   -5.60379500    1.34727200 H                  0.28852500   -0.78556500    5.18292300 H                  0.90287800   -1.14782800    2.78958600 H                  0.99885500   -1.50285500   -2.57530100 H                  0.46682500   -1.47777100   -5.01696800 C                  0.94082400    3.34417800   -0.18592200 C                  1.97168700    4.10750400   -0.76001800 C                 -0.19920200    3.99915000    0.31304300 C                  1.86545400    5.49619700   -0.82559100 H                  2.84375600    3.62089300   -1.18386500 C                 -0.29584200    5.38751700    0.25663700 H                 -0.99587600    3.40943100    0.75325800 C                  0.73590400    6.13991600   -0.31313000 H                  2.66283400    6.07467400   -1.28175200 H                 -1.17488400    5.88333100    0.65704000 H                  0.65832400    7.22194300   -0.36086600 C                  3.85692600   -0.71501300    0.10387800 C                  4.09381800   -2.01883800   -0.36657900 C                  4.93089100    0.02942000    0.62187200 C                  5.37849300   -2.55673500   -0.33896700 H                  3.26477300   -2.59513400   -0.76219000 C                  6.21287400   -0.51701500    0.65926500 H                  4.76541500    1.02307100    1.02458400 C                  6.44165000   -1.80766200    0.17428800 H                  5.55118300   -3.55966000   -0.71740900 H                  7.03210400    0.06336700    1.07227100 H                  7.44215200   -2.22884700    0.200221004-3MLCTRu                 0.00009700   -0.28181000    0.11899700 O                  0.00136000   -2.33426300    0.09803700 O                  0.00085300   -0.24204300   -1.90067300 N                 -2.07698300    0.16909100    0.22865200 N                 -0.00111900    1.71853800   -0.04902400 N                  2.07609800    0.17107900    0.23017200 C                  3.10748900   -0.67957500    0.50941200 C                  4.42657100   -0.25174200    0.41136700 C                  3.67769000    1.94282500   -0.20497100 H                  3.87398700    2.97365400   -0.47327700 C                  4.71935600    1.06783500    0.03189400 H                  5.74837100    1.39994200   -0.05528700 C                  2.35224500    1.49648400   -0.08320300 C                  1.20105700    2.35618500   -0.23241200 C                  1.21019600    3.71795400   -0.52823800 H                  2.14808000    4.24489100   -0.65625600 C                 -0.00216800    4.40379300   -0.66511400 H                 -0.00268900    5.46437000   -0.88874300 C                 -1.21427100    3.71654000   -0.52877600 H                 -2.15262800    4.24245200   -0.65736600 C                 -1.20347300    2.35512800   -0.23292200 C                 -2.35429400    1.49404300   -0.08401900 C                 -3.67982900    1.93947300   -0.20562100 H                 -3.87696100    2.97036200   -0.47301000 C                 -4.72091700    1.06328700    0.03030000 H                 -5.75018700    1.39460200   -0.05680400 C                 -4.42707200   -0.25613100    0.40855800 C                 -3.10750500   -0.68283100    0.50674500 C                  0.00345200   -0.91807100   -4.14715400 H                  0.00276800   -1.80217400   -4.78569200 H                 -0.87745800   -0.30846900   -4.37267400 C                  0.00239400   -1.26701500   -2.68092600 C                  0.00317900   -2.61086800   -2.27692800 H                  0.00430200   -3.35748000   -3.06085600 C                  0.00252800   -3.07509900   -0.95248800 C                  0.00302400   -4.55512300   -0.66786100 H                  0.00555600   -5.15771900   -1.57682200 H                 -0.87928700   -4.80539100   -0.06970900 N                 -0.00082000   -0.34800800    2.16523600 C                 -0.00133200   -0.40211400    3.31988400 C                 -0.00190800   -0.47949100    4.77188300 H                 -0.89159600   -1.01741600    5.10968400 H                  0.89122400   -1.01102900    5.11070400 H                 -0.00574300    0.52810800    5.19545800 H                  0.88615700   -0.31069600   -4.37177700 H                  0.88280300   -4.80407600   -0.06548500 H                  5.22214800   -0.95177700    0.63805400 H                 -5.22205600   -0.95716500    0.63423700 C                 -2.81223700   -2.09889500    0.91236600 H                 -2.51714000   -2.69634900    0.04555200 H                 -1.99562300   -2.14863400    1.63369600 H                 -3.70389900   -2.55279100    1.34828500 C                  2.81350200   -2.09560300    0.91621500 H                  1.99624700   -2.14562300    1.63680300 H                  2.52002500   -2.69439600    0.04977300 H                  3.70529900   -2.54793100    1.353505003-3TSIfreq = -192.68 cm-1 Ru                -0.42377100   -0.73448700    0.04455000 O                  1.58037000   -1.11356800    0.05675800 O                 -0.08337600    1.24786300   -0.09182000 N                 -1.04854300   -0.99279000   -2.06011900 N                 -2.45074900   -0.24276600    0.01414800 N                 -1.02480600   -0.65010700    2.16798400 C                 -0.14822800   -0.73420800    3.18997500 C                 -0.43104200   -0.23391900    4.45194200 C                 -2.57319200    0.48813600    3.61915900 H                 -3.52407300    0.98977300    3.75669500 C                 -1.66689700    0.39491800    4.66818700 H                 -1.91125300    0.81131000    5.63945900 C                 -2.23778400   -0.05366700    2.37138500 C                 -3.09993800   -0.01680800    1.18969100 C                 -4.47369100    0.23011900    1.20029000 H                 -4.99166000    0.39412200    2.13756800 C                 -5.17322400    0.24166100   -0.00694100 H                 -6.24398700    0.41207900   -0.01368500 C                 -4.49236000    0.01815200   -1.20460600 H                 -5.02532800    0.01686100   -2.14778700 C                 -3.11826200   -0.22321000   -1.17257200 C                 -2.27364500   -0.46022000   -2.34454700 C                 -2.63774300   -0.15934600   -3.66366400 H                 -3.59873000    0.29559600   -3.87360600 C                 -1.74677700   -0.43142500   -4.69471900 H                 -2.01377900   -0.20330300   -5.72094900 C                 -0.49793900   -0.99427800   -4.39135800 C                 -0.18694800   -1.25159600   -3.06448500 C                  1.07278000    1.81987900   -0.14135200 C                  2.31358200    1.16187900   -0.08331300 H                  3.19555900    1.78414000   -0.11946000 C                  2.51708400   -0.22395700    0.01938000 N                 -0.70591900   -2.79579700    0.20116500 C                 -0.84616400   -3.94070300    0.28916400 C                 -1.02180100   -5.38134900    0.39932100 H                 -2.00165300   -5.60189700    0.83076300 H                 -0.95426100   -5.83770500   -0.59175900 H                 -0.24305600   -5.80020300    1.04198000 H                  0.29508500   -0.34073000    5.24975400 H                  0.79425000   -1.22348500    2.96479700 H                  0.76666400   -1.67889500   -2.77007000 H                  0.21677800   -1.23531500   -5.16994300 C                  1.01701500    3.30489000   -0.24637400 C                  2.03996300    4.05035200   -0.85667100 C                 -0.10391800    3.97902900    0.26947200 C                  1.94555000    5.43914300   -0.94068500 H                  2.89688200    3.54813500   -1.29328700 C                 -0.18948100    5.36751000    0.19398800 H                 -0.89518700    3.40354500    0.73758500 C                  0.83484000    6.10183900   -0.41154200 H                  2.73732000    6.00282100   -1.42457100 H                 -1.05430900    5.87752900    0.60758100 H                  0.76600400    7.18374500   -0.47411600 C                  3.89991100   -0.77533500    0.07437100 C                  4.12686400   -2.08328100   -0.38889500 C                  4.98041700   -0.03759800    0.58778300 C                  5.40705400   -2.63211000   -0.35800500 H                  3.29264700   -2.65451500   -0.78125200 C                  6.25829400   -0.59419000    0.62820000 H                  4.82249400    0.95990700    0.98416600 C                  6.47675700   -1.88941400    0.15100300 H                  5.57121500   -3.63873100   -0.73069100 H                  7.08228400   -0.01796400    1.03782800 H                  7.47371200   -2.31888500    0.17956300 4-3TSI freq = -179.29 cm-1Ru                 0.00001800   -0.30660300    0.13337500 O                  0.00022800   -2.34811200    0.31971000 O                 -0.00002700   -0.46468200   -1.87817500 N                 -2.11812800    0.24469700    0.27526300 N                 -0.00011600    1.71287300   -0.20393000 N                  2.11808600    0.24496700    0.27525800 C                  3.16465500   -0.60163800    0.48264200 C                  4.46541600   -0.21316000    0.17273600 C                  3.65385900    1.92621400   -0.55175600 H                  3.81462700    2.91282900   -0.96923100 C                  4.71491500    1.05476300   -0.36807600 H                  5.72428900    1.35258300   -0.63123200 C                  2.36175600    1.51539300   -0.19979500 C                  1.19087600    2.37214600   -0.30111400 C                  1.20819600    3.75543200   -0.48177100 H                  2.14999900    4.28751900   -0.53916400 C                 -0.00028400    4.45054000   -0.56613100 H                 -0.00035000    5.52822700   -0.68459000 C                 -1.20867900    3.75528700   -0.48173500 H                 -2.15054700    4.28726100   -0.53910200 C                 -1.19118700    2.37200300   -0.30108000 C                 -2.36196200    1.51510500   -0.19974800 C                 -3.65412200    1.92577800   -0.55167200 H                 -3.81502100    2.91238800   -0.96910700 C                 -4.71506500    1.05418400   -0.36801300 H                 -5.72448000    1.35188800   -0.63114300 C                 -4.46539800   -0.21373300    0.17273300 C                 -3.16458300   -0.60205600    0.48260900 C                 -0.00006800   -1.36338100   -4.04660900 H                  0.00017000   -2.30721000   -4.59331200 H                 -0.88205800   -0.78123000   -4.33272000 C                  0.00005300   -1.56363800   -2.55218400 C                  0.00022300   -2.85996200   -2.01650800 H                  0.00029200   -3.68157900   -2.72149900 C                  0.00031100   -3.18963500   -0.65209100 C                  0.00053700   -4.63491000   -0.22231700 H                  0.00053800   -5.32409900   -1.06770400 H                 -0.88062900   -4.82480600    0.39931400 N                  0.00000400   -0.19028200    2.20542000 C                 -0.00002900   -0.14461400    3.36094000 C                 -0.00008300   -0.09308400    4.81475200 H                 -0.89171600   -0.59456400    5.20015400 H                  0.89082300   -0.59577200    5.20025800 H                  0.00061000    0.94787900    5.14804400 H                  0.88155400   -0.78071900   -4.33280400 H                  0.88189100   -4.82458000    0.39911900 H                  5.28033900   -0.90106300    0.36833800 H                 -5.28023000   -0.90175300    0.36830200 C                 -2.89446900   -1.96346500    1.05935000 H                 -2.54688500   -2.64926100    0.28120500 H                 -2.11859500   -1.92389300    1.82538200 H                 -3.80816300   -2.37705800    1.49090600 C                  2.89473800   -1.96305100    1.05947000 H                  2.11884200   -1.92355000    1.82548200 H                  2.54728400   -2.64896200    0.28136700 H                  3.80848900   -2.37646700    1.49107500 3-3MC Ru                -0.34739700   -0.75963900    0.04378600 O                  1.65885400   -1.13630400    0.03441600 O                  0.00044300    1.23592900   -0.12961300 N                 -1.16653100   -1.01837300   -2.17585800 N                 -2.47209300   -0.24664100    0.03893200 N                 -1.08564900   -0.60493200    2.31415900 C                 -0.27162300   -0.68000000    3.37504900 C                 -0.61664700   -0.15838500    4.61995700 C                 -2.70172600    0.55303200    3.64978700 H                 -3.65468800    1.06078400    3.73382000 C                 -1.85361200    0.47453000    4.75242600 H                 -2.15415100    0.90783800    5.70052600 C                 -2.28976200   -0.01021100    2.43564500 C                 -3.11492200    0.01258500    1.20478500 C                 -4.48727700    0.27972700    1.23232700 H                 -4.99290700    0.45259000    2.17359600 C                 -5.20315400    0.29368900    0.04030700 H                 -6.27097900    0.48238700    0.04306600 C                 -4.53370100    0.05242200   -1.15427200 H                 -5.07547400    0.04702900   -2.09131200 C                 -3.16035300   -0.20940700   -1.12940300 C                 -2.38218400   -0.46603500   -2.36407700 C                 -2.84563100   -0.15286000   -3.64796800 H                 -3.80793600    0.32252100   -3.79292200 C                 -2.03759300   -0.44022300   -4.74601400 H                 -2.37817000   -0.20178300   -5.74808600 C                 -0.78867700   -1.02881400   -4.54075600 C                 -0.39148800   -1.29579400   -3.23255000 C                  1.15432300    1.80576400   -0.18355900 C                  2.39479300    1.14246500   -0.11895800 H                  3.27974300    1.76056400   -0.15794000 C                  2.59378900   -0.24201000   -0.00587100 N                 -0.62964800   -2.79747000    0.22436000 C                 -0.77623100   -3.94225500    0.32143700 C                 -0.96257500   -5.38103100    0.44253100 H                 -0.28922500   -5.77933100    1.20618200 H                 -1.99558900   -5.59836200    0.72697000 H                 -0.74529100   -5.86464000   -0.51367000 H                  0.57380800   -1.74140900   -3.00949100 H                  0.68214700   -1.17239900    3.20813200 H                  0.06791200   -0.24505200    5.45613200 H                 -0.13422600   -1.27312800   -5.36980200 C                  1.10673200    3.29158900   -0.29711300 C                  2.13771100    4.03232300   -0.89953000 C                 -0.01539100    3.97257200    0.20702600 C                  2.05040800    5.42157800   -0.98681400 H                  2.99693500    3.52580400   -1.32663300 C                 -0.09487200    5.36130300    0.12795100 H                 -0.81306200    3.40100200    0.66912900 C                  0.93802000    6.09050500   -0.46919600 H                  2.84943100    5.98088800   -1.46397400 H                 -0.96139300    5.87565000    0.53279500 H                  0.87468000    7.17266000   -0.53390200 C                  3.97768000   -0.79323900    0.05957200 C                  4.20980500   -2.10195700   -0.39867800 C                  5.05412800   -0.05530100    0.58120900 C                  5.48992200   -2.65063000   -0.35594500 H                  3.37885000   -2.67459900   -0.79590000 C                  6.33206500   -0.61119300    0.63341400 H                  4.89212300    0.94270000    0.97484000 C                  6.55545000   -1.90711600    0.16050100 H                  5.65716700   -3.65814100   -0.72508200 H                  7.15216200   -0.03386800    1.04948800 H                  7.55220700   -2.33639700    0.198572004-3MC Ru                 0.00000900   -0.42783500    0.14198100 O                 -0.00007600   -2.47086300    0.29704500 O                 -0.00018500   -0.55547600   -1.89470300 N                 -2.27965600    0.34702000    0.34005100 N                  0.00007900    1.71265100   -0.15640100 N                  2.27972800    0.34666700    0.33973300 C                  3.35713100   -0.44707200    0.49248600 C                  4.62875200   -0.01083200    0.10170800 C                  3.65685000    2.07263000   -0.60115200 H                  3.75041900    3.05393500   -1.04902700 C                  4.77513800    1.25499800   -0.46056100 H                  5.74986500    1.60343700   -0.78608700 C                  2.41666900    1.58690000   -0.17290100 C                  1.17289500    2.38834500   -0.25233900 C                  1.19821700    3.77841900   -0.40482100 H                  2.14183600    4.30704300   -0.44249200 C                  0.00021700    4.47979000   -0.47631100 H                  0.00027600    5.55956700   -0.57653000 C                 -1.19785600    3.77854000   -0.40485400 H                 -2.14144100    4.30722600   -0.44255700 C                 -1.17265500    2.38847100   -0.25234900 C                 -2.41649000    1.58713400   -0.17288200 C                 -3.65659300    2.07281700   -0.60139900 H                 -3.75006100    3.05402300   -1.04951800 C                 -4.77492000    1.25524100   -0.46076300 H                 -5.74959800    1.60364500   -0.78647400 C                 -4.62864600   -0.01047400    0.10179300 C                 -3.35707800   -0.44667600    0.49281800 C                 -0.00040900   -1.43799900   -4.07442500 H                  0.00055300   -2.37897400   -4.62660400 H                 -0.88277600   -0.85569200   -4.35976800 C                 -0.00021400   -1.64400800   -2.57882500 C                 -0.00009400   -2.94783300   -2.05295500 H                 -0.00004300   -3.76272900   -2.76615400 C                 -0.00010600   -3.29477500   -0.69426300 C                 -0.00026500   -4.74847900   -0.28760800 H                  0.00157600   -5.42415200   -1.14423700 H                 -0.88289500   -4.95068400    0.32827700 N                  0.00003000   -0.37723800    2.20841400 C                 -0.00001800   -0.36922900    3.36656500 C                 -0.00005600   -0.35778800    4.82222500 H                  0.00012200    0.67327500    5.18563400 H                 -0.89081400   -0.86931400    5.19666600 H                  0.89047400   -0.86966800    5.19670800 H                  0.88065200   -0.85376300   -4.35980500 H                  0.88009800   -4.94998100    0.33170300 H                  5.48531500   -0.66181900    0.23833700 H                 -5.48525400   -0.66139100    0.23846800 C                 -3.13457600   -1.80925500    1.08994600 H                 -2.58564300   -2.44723200    0.39069000 H                 -2.52494900   -1.73385400    1.99415000 H                 -4.08331800   -2.29155000    1.33333200 C                  3.13451200   -1.80976200    1.08931800 H                  2.52503800   -1.73446700    1.99363800 H                  2.58536300   -2.44749000    0.39000500 H                  4.08321800   -2.29225900    1.332449003-3TSII freq = -77.16 cm-1Ru                -0.60821600   -0.49958200    0.10356100 O                  1.21033500   -1.44244100    0.20751600 O                  0.33036300    1.19799100   -0.64856100 N                 -1.77733500   -1.85836100   -1.19120200 N                 -2.42570800    0.43391300   -0.10008400 N                 -0.80915500    0.98463900    1.95030800 C                  0.16605600    1.26011700    2.82707200 C                  0.32959100    2.52191900    3.39567800 C                 -1.57291800    3.25684100    2.11678300 H                 -2.25441200    4.03749100    1.80041200 C                 -0.55568900    3.53639200    3.02791000 H                 -0.45166100    4.53573700    3.43699800 C                 -1.67591600    1.96337100    1.59429200 C                 -2.70330100    1.55977800    0.61174300 C                 -3.90849700    2.24034400    0.43018500 H                 -4.12597100    3.12573800    1.01457700 C                 -4.84066500    1.74987000   -0.48108100 H                 -5.78460600    2.26253800   -0.62797000 C                 -4.56190700    0.57932700   -1.18102800 H                 -5.28962400    0.17100400   -1.87065300 C                 -3.34359500   -0.07060600   -0.97215200 C                 -2.95162400   -1.33073900   -1.62768900 C                 -3.70435700   -1.97558300   -2.61496800 H                 -4.63332500   -1.54690400   -2.97006300 C                 -3.24332100   -3.17822700   -3.14341100 H                 -3.81603700   -3.68962700   -3.90955900 C                 -2.04073800   -3.71548200   -2.67760600 C                 -1.33735700   -3.02119800   -1.69957100 C                  1.60397500    1.36264600   -0.76485300 C                  2.58266300    0.38305100   -0.52652700 H                  3.60933700    0.67337900   -0.69638800 C                  2.35785800   -0.93589100   -0.08669400 N                 -1.27998300   -2.14436400    1.71178200 C                 -1.62865400   -2.87940100    2.53669000 C                 -2.06535700   -3.80268000    3.57598100 H                 -1.38157700   -3.75108300    4.42713000 H                 -3.07225200   -3.53655800    3.90773400 H                 -2.07441600   -4.82298900    3.18417400 H                 -0.39598200   -3.38430400   -1.30012600 H                  0.83317400    0.43762300    3.06726500 H                  1.13162800    2.70006800    4.10298200 H                 -1.65332500   -4.65227500   -3.06130400 C                  2.01289100    2.73971500   -1.16608000 C                  3.16657300    2.99201300   -1.92636900 C                  1.20530400    3.81999600   -0.76929700 C                  3.50637700    4.29841400   -2.27791400 H                  3.78435500    2.16810000   -2.26884800 C                  1.55551400    5.12484800   -1.11006200 H                  0.31540900    3.62354100   -0.18080900 C                  2.70606800    5.36792000   -1.86682800 H                  4.39345600    4.48037200   -2.87710900 H                  0.93198400    5.95265600   -0.78545600 H                  2.97567500    6.38450500   -2.13740300 C                  3.50864700   -1.87369000    0.06621100 C                  3.27655300   -3.25454300   -0.05923100 C                  4.81210800   -1.42737900    0.34362900 C                  4.32424000   -4.16415500    0.06859300 H                  2.27044500   -3.60147600   -0.26776000 C                  5.85687400   -2.34069200    0.48295700 H                  5.01086800   -0.36970000    0.48055600 C                  5.61839600   -3.71018700    0.34019000 H                  4.13202700   -5.22704900   -0.04382700 H                  6.85670400   -1.98209800    0.70820500 H                  6.43458000   -4.41893900    0.443973004-3TSIIfreq = -68.72 cm-1Ru                -0.01490500   -0.38142600    0.01800000 O                  0.39959100   -2.34011200    0.47478400 O                  0.74421700   -0.69906300   -1.92756100 N                 -2.24507700   -0.25499300    0.01640300 N                 -0.39408900    1.54040400   -0.49477600 N                  1.90962600    0.73924400    0.53696600 C                  3.04645700    0.22645900    1.05363700 C                  4.26529900    0.90012700    0.90890900 C                  3.13262500    2.63451500   -0.30614700 H                  3.14753300    3.56849300   -0.85353800 C                  4.31031800    2.10510300    0.21400200 H                  5.25037700    2.63040400    0.08177000 C                  1.94015200    1.93325900   -0.11541200 C                  0.63100800    2.43211000   -0.57493400 C                  0.40386800    3.73540200   -1.02184600 H                  1.21828800    4.44671600   -1.07169600 C                 -0.88560300    4.12303600   -1.37653500 H                 -1.07761800    5.13349900   -1.71930400 C                 -1.93076900    3.21118600   -1.26031700 H                 -2.94243800    3.51177800   -1.50071000 C                 -1.66354800    1.91608200   -0.81042600 C                 -2.68656100    0.87947900   -0.60002600 C                 -4.02254700    1.03873900   -0.97443500 H                 -4.35175800    1.93388800   -1.48620300 C                 -4.92725300    0.02385900   -0.68244800 H                 -5.97066900    0.12347500   -0.96216000 C                 -4.47710300   -1.11471400   -0.01948400 C                 -3.12611900   -1.23721200    0.32133200 C                  1.75387800   -1.75058200   -3.77815200 H                  2.16720600   -2.70318900   -4.11373200 H                  0.93504600   -1.45972100   -4.44451100 C                  1.24477800   -1.80318300   -2.35582000 C                  1.34657000   -3.00066300   -1.62832700 H                  1.78973800   -3.84624200   -2.13978100 C                  0.92929400   -3.21314600   -0.30187400 C                  1.10794600   -4.57457900    0.32871300 H                  1.54849400   -5.30142600   -0.35536100 H                  0.13572300   -4.94468000    0.67030700 N                 -0.42264300   -0.08261300    2.46408700 C                 -0.64738700    0.48933200    3.44810900 C                 -0.92869400    1.21427100    4.68064800 H                 -1.75602700    1.91031500    4.51909300 H                 -1.20128600    0.51168000    5.47228200 H                 -0.04311900    1.77579900    4.98941700 H                  2.52521900   -0.97715600   -3.85590200 H                  1.74723400   -4.47991100    1.21288900 H                  5.16359500    0.47335700    1.34117500 H                 -5.16305000   -1.91340900    0.23961400 C                 -2.61378800   -2.45771000    1.03053700 H                 -1.99503800   -3.06024500    0.35977700 H                 -1.97900600   -2.17339200    1.87210800 H                 -3.44327500   -3.07068500    1.38802800 C                  2.96809400   -1.09203200    1.77076600 H                  2.03905700   -1.17110300    2.33671700 H                  2.97950100   -1.91384900    1.04765300 H                  3.82139800   -1.21735800    2.440722003-[3Rup:ACN]Ru                -0.60078900   -0.42502800   -0.00958500 O                  1.11435100   -1.53012700    0.20963000 O                  0.59865200    1.18415600   -0.78998900 N                 -1.73380700   -1.67760300   -1.23493900 N                 -2.30359700    0.59717200   -0.19823100 N                 -0.62130600    0.84587100    1.75588100 C                  0.33514800    0.89880100    2.69732400 C                  0.47094900    1.98662500    3.55448000 C                 -1.40727800    3.00939100    2.44555000 H                 -2.09452300    3.83731000    2.32043800 C                 -0.41298400    3.05868300    3.42066500 H                 -0.32903600    3.92621400    4.06619600 C                 -1.49634800    1.88627700    1.62138600 C                 -2.52007300    1.69149400    0.58141000 C                 -3.67504900    2.45414300    0.41042100 H                 -3.85562100    3.32316100    1.03097400 C                 -4.61485700    2.06128800   -0.54441900 H                 -5.51923800    2.64175700   -0.68581500 C                 -4.41050200    0.89729300   -1.28535000 H                 -5.15850400    0.55829100   -1.99123500 C                 -3.23946000    0.16375900   -1.08811400 C                 -2.89993500   -1.12596000   -1.69885300 C                 -3.68394200   -1.79954900   -2.63740100 H                 -4.59976300   -1.35232700   -3.00449700 C                 -3.27776000   -3.05029700   -3.09372500 H                 -3.87812600   -3.58452100   -3.82183000 C                 -2.09359000   -3.60802000   -2.60270800 C                 -1.34741400   -2.89148000   -1.67683100 C                  1.88384100    1.19270800   -0.81003100 C                  2.72464500    0.12898800   -0.42595500 H                  3.78749800    0.30617000   -0.50165100 C                  2.33038700   -1.13933400    0.03896800 N                 -2.46632600   -2.89712100    1.93554900 C                 -3.14252800   -1.98498200    2.17950300 C                 -3.97808800   -0.82653200    2.47863600 H                 -3.36060400   -0.02371500    2.88899100 H                 -4.46688900   -0.47193000    1.56756400 H                 -4.74501500   -1.09338800    3.20974300 H                 -0.41608200   -3.26653200   -1.26770300 H                  1.00120600    0.04411300    2.74523100 H                  1.25375200    1.98855500    4.30389000 H                 -1.74878400   -4.58137000   -2.93147800 C                  2.49633400    2.47843700   -1.26147500 C                  3.73144600    2.53130200   -1.92855000 C                  1.80002900    3.67475200   -1.01769100 C                  4.26002900    3.75594100   -2.33650900 H                  4.26798100    1.61579400   -2.15578100 C                  2.33765100    4.89800600   -1.41289700 H                  0.84478600    3.63102600   -0.50517400 C                  3.56866000    4.94197600   -2.07487600 H                  5.20952800    3.78316800   -2.86256100 H                  1.79760300    5.81712100   -1.20580200 H                  3.98505400    5.89472900   -2.38825400 C                  3.36406400   -2.16234900    0.37422600 C                  3.02226400   -3.52336300    0.29104100 C                  4.65849700   -1.81204400    0.79477800 C                  3.95708200   -4.50913000    0.60013200 H                  2.02156800   -3.79632900   -0.02543600 C                  5.58848200   -2.80052800    1.11569800 H                  4.93512200   -0.76863300    0.90297000 C                  5.24357400   -4.15084900    1.01404300 H                  3.68258900   -5.55668900    0.51940700 H                  6.58096700   -2.51513800    1.45103500 H                  5.97083500   -4.91875600    1.260305004-[3Rup:ACN]Ru                -0.05898500   -0.40651000   -0.03550900 O                  0.41935000   -2.37059800    0.36064500 O                  0.79755500   -0.64308300   -1.99095900 N                 -2.20152200   -0.34579800    0.11142700 N                 -0.48123700    1.47909200   -0.52285100 N                  1.76996000    0.69419100    0.53255400 C                  2.87399500    0.21824100    1.15309500 C                  4.07326200    0.93933100    1.12271400 C                  2.99402200    2.64989400   -0.17069700 H                  3.01897500    3.59766100   -0.69325500 C                  4.13892100    2.15244500    0.44488500 H                  5.06733200    2.71211900    0.40550600 C                  1.81640800    1.90690500   -0.09997600 C                  0.53030600    2.37950800   -0.63459800 C                  0.27990300    3.65044600   -1.15424600 H                  1.07876100    4.37547600   -1.24495200 C                 -1.01879600    3.98668500   -1.53504300 H                 -1.22760100    4.96971700   -1.94146200 C                 -2.05368100    3.06942100   -1.36044100 H                 -3.07083700    3.33859000   -1.61579200 C                 -1.76174100    1.80897900   -0.83625200 C                 -2.73333500    0.76097900   -0.50428200 C                 -4.10263200    0.88536800   -0.73385300 H                 -4.49724100    1.75848400   -1.23766700 C                 -4.95271800   -0.12463800   -0.29748300 H                 -6.02192600   -0.05187700   -0.46467800 C                 -4.41389100   -1.21791300    0.37513200 C                 -3.03378800   -1.31379300    0.57614100 C                  1.90024900   -1.61822000   -3.83038000 H                  2.34189100   -2.55328700   -4.17897500 H                  1.10247900   -1.31833000   -4.51786400 C                  1.33764700   -1.72143700   -2.43052200 C                  1.44024400   -2.93986900   -1.73529000 H                  1.91898700   -3.75887500   -2.25763500 C                  0.98923200   -3.20544000   -0.43030400 C                  1.17790200   -4.58358400    0.15920600 H                  1.67951900   -5.26995800   -0.52445600 H                  0.20016300   -4.99633100    0.42946000 N                 -0.44483200   -0.42314200    3.72387000 C                 -0.34337700    0.69720800    3.43470600 C                 -0.21563000    2.09610200    3.04095400 H                 -0.80978800    2.27570400    2.14160400 H                 -0.56798400    2.74974000    3.84244200 H                  0.83090000    2.32536200    2.82556400 H                  2.66090700   -0.83078700   -3.85351400 H                  1.76078300   -4.50310400    1.08265300 H                  4.94298300    0.54006900    1.63195500 H                 -5.05512000   -2.00539600    0.75397000 C                 -2.44104500   -2.47403500    1.31946700 H                 -1.84851900   -3.10298800    0.65089200 H                 -1.76759100   -2.11877400    2.10442300 H                 -3.23010100   -3.07933400    1.76919700 C                  2.78192500   -1.09824200    1.86913100 H                  1.82559600   -1.19311800    2.38648700 H                  2.85349300   -1.92349900    1.15498300 H                  3.59712500   -1.19784000    2.588629005-GSRu                -0.00031700   -0.22217700    0.22657600 O                 -0.00038900   -2.25955700    0.74320200 O                 -0.00140500   -0.70645200   -1.78226300 N                 -2.06004500    0.12183000    0.09767500 N                 -0.00040400    1.66827400   -0.31895000 N                  2.05932300    0.12144800    0.09574200 C                  3.05518400   -0.74514500    0.33688500 C                  4.40708800   -0.41397500    0.21328000 C                  3.68343300    1.80433000   -0.43010200 H                  3.91743800    2.81819100   -0.73234100 C                  4.70752700    0.89715300   -0.17881400 H                  5.74240300    1.20735000   -0.28651500 C                  2.35440600    1.40086300   -0.29021600 C                  1.19011600    2.27618100   -0.54236000 C                  1.21195000    3.60066100   -0.98439000 H                  2.15060500    4.10915200   -1.16735200 C                 -0.00058200    4.26036100   -1.19613800 H                 -0.00064900    5.28900000   -1.53901200 C                 -1.21303000    3.60090500   -0.98318200 H                 -2.15176700    4.10957600   -1.16522200 C                 -1.19102200    2.27642100   -0.54116100 C                 -2.35524100    1.40128600   -0.28805800 C                 -3.68431300    1.80491000   -0.42707600 H                 -3.91838900    2.81882400   -0.72908100 C                 -4.70834600    0.89781500   -0.17526500 H                 -5.74325700    1.20812500   -0.28230900 C                 -4.40779600   -0.41341100    0.21642600 C                 -3.05585600   -0.74470800    0.33924800 C                 -0.00108900   -1.97524500   -3.75761600 H                 -0.00472600   -3.00064200   -4.13072700 H                 -0.88028300   -1.44972100   -4.14493500 C                 -0.00130900   -1.90302500   -2.24649000 C                 -0.00101300   -3.09444500   -1.50284000 H                 -0.00108800   -4.01642600   -2.07128500 C                 -0.00063900   -3.22052200   -0.09874300 C                 -0.00043400   -4.60269400    0.51735600 H                 -0.00047200   -5.39882100   -0.22903700 H                 -0.88179400   -4.71267100    1.15802400 N                  0.00164300    0.21822500    2.19128400 C                  0.00408200    0.46019900    3.32511800 C                  0.00842300    0.76209300    4.74972600 H                 -1.00693100    0.69140900    5.14911000 H                  0.65037400    0.05214200    5.27837300 H                  0.38658700    1.77482600    4.91367700 H                  0.88253500   -1.45657000   -4.14421300 H                  0.88111400   -4.71252400    1.15779200 H                 -2.74256500   -1.73872900    0.64207700 H                  2.74198100   -1.73911100    0.63997900 C                  5.48521200   -1.42748300    0.48833500 H                  5.06508200   -2.38132400    0.81611100 H                  6.08386600   -1.60833300   -0.41076500 H                  6.16705200   -1.06693100    1.26548000 C                 -5.48584900   -1.42692800    0.49173500 H                 -6.08395300   -1.60863100   -0.40756700 H                 -5.06568800   -2.38042600    0.82046500 H                 -6.16821000   -1.06591700    1.268201005-3MLCTRu                -0.00035200   -0.27426400    0.15436400 O                 -0.00084200   -2.28146700    0.56307600 O                 -0.00253000   -0.63424100   -1.83951000 N                 -2.03553800    0.17476100    0.15408700 N                 -0.00005300    1.67624900   -0.32684800 N                  2.03517500    0.17327600    0.15027000 C                  3.01764200   -0.70747800    0.45048100 C                  4.36421500   -0.39910900    0.34556600 C                  3.69866700    1.82137900   -0.38223400 H                  3.95302700    2.82330300   -0.70872500 C                  4.69341400    0.90909000   -0.08785100 H                  5.73619100    1.19593200   -0.18395500 C                  2.34706700    1.45438800   -0.25862800 C                  1.20725300    2.30946900   -0.50902700 C                  1.21616500    3.64803800   -0.89207800 H                  2.15580400    4.16896600   -1.03590500 C                  0.00019600    4.31883400   -1.08436800 H                  0.00030500    5.36215200   -1.37842100 C                 -1.21587400    3.64885200   -0.89012500 H                 -2.15540000    4.17041000   -1.03240400 C                 -1.20726900    2.31025400   -0.50707100 C                 -2.34721900    1.45596300   -0.25482700 C                 -3.69878100    1.82363500   -0.37697100 H                 -3.95297900    2.82559200   -0.70349600 C                 -4.69367200    0.91192500   -0.08133700 H                 -5.73640400    1.19926400   -0.17643400 C                 -4.36467500   -0.39639100    0.35195200 C                 -3.01816300   -0.70542200    0.45552200 C                 -0.00568700   -1.76774900   -3.89405200 H                 -0.00321700   -2.76677600   -4.33147200 H                 -0.88976300   -1.22347500   -4.24144100 C                 -0.00371300   -1.79820100   -2.38671100 C                 -0.00363300   -3.03087400   -1.71041500 H                 -0.00472200   -3.92155600   -2.32558400 C                 -0.00230300   -3.22098700   -0.32168400 C                 -0.00250400   -4.61430000    0.25178200 H                 -0.00348100   -5.38508000   -0.51998000 H                 -0.88367300   -4.74007100    0.88939900 N                  0.00288300    0.05350500    2.17259900 C                  0.00730100    0.22674900    3.31531000 C                  0.01418200    0.44156700    4.75378800 H                 -1.01123100    0.42768000    5.13208500 H                  0.58943300   -0.34989400    5.24120700 H                  0.46881700    1.40979600    4.97886800 H                  0.87386000   -1.21776600   -4.24380200 H                  0.87953200   -4.74090500    0.88803000 H                 -2.68414100   -1.68500300    0.78220100 H                  2.68346400   -1.68693800    0.77736100 C                  5.43003900   -1.40738400    0.68090900 H                  4.99679800   -2.35686600    1.00565800 H                  6.06850600   -1.60372000   -0.18773000 H                  6.08004000   -1.03746900    1.48171300 C                 -5.43067700   -1.40409300    0.68846100 H                 -6.07010300   -1.60018800   -0.17955800 H                 -4.99758000   -2.35378300    1.01286700 H                 -6.07972800   -1.03377600    1.489884005-TSI? = -180.93 cm-1Ru                -0.00053600   -0.33550000    0.22693800 O                 -0.00144800   -2.33471000    0.67529700 O                 -0.00308700   -0.73564100   -1.75835300 N                 -2.11977400    0.28086000    0.30387300 N                  0.00008300    1.67758900   -0.31977700 N                  2.11970100    0.27857000    0.29920800 C                  3.13551900   -0.58640600    0.49622400 C                  4.44825800   -0.31789200    0.12294900 C                  3.66038700    1.81855300   -0.70917100 H                  3.84564000    2.76368800   -1.20665600 C                  4.69710500    0.92123300   -0.50142600 H                  5.70289900    1.16964100   -0.82633800 C                  2.36537300    1.48389500   -0.29063900 C                  1.18644500    2.33346000   -0.45413800 C                  1.20812300    3.70153100   -0.72973700 H                  2.15242600    4.22459200   -0.82176300 C                  0.00084400    4.38894200   -0.86357000 H                  0.00117400    5.45553900   -1.05895800 C                 -1.20681000    3.70268000   -0.72753700 H                 -2.15080200    4.22661900   -0.81778800 C                 -1.18592600    2.33456900   -0.45196300 C                 -2.36529300    1.48609600   -0.28635000 C                 -3.66052800    1.82146600   -0.70364300 H                 -3.84560100    2.76648300   -1.20142600 C                 -4.69763800    0.92493900   -0.49450100 H                 -5.70358200    1.17383800   -0.81857100 C                 -4.44894300   -0.31412700    0.13011000 C                 -3.13605400   -0.58334200    0.50225700 C                 -0.00594400   -1.90984100   -3.79227300 H                 -0.00716900   -2.91732800   -4.21050000 H                 -0.88762500   -1.36958100   -4.15220800 C                 -0.00440500   -1.91126100   -2.28350900 C                 -0.00447400   -3.12904900   -1.58429600 H                 -0.00571800   -4.03269600   -2.18063900 C                 -0.00308700   -3.28897600   -0.19051700 C                 -0.00345600   -4.67277800    0.40993400 H                 -0.00459900   -5.45838000   -0.34700700 H                 -0.88457900   -4.78798900    1.04975400 N                  0.00332400    0.01151000    2.28430700 C                  0.00838200    0.19830100    3.42604000 C                  0.01631000    0.43391700    4.86243700 H                 -1.00693800    0.41050400    5.24621400 H                  0.60498500   -0.34082400    5.36066100 H                  0.45703400    1.41179700    5.07338900 H                  0.87588000   -1.37100100   -4.15398900 H                  0.87858800   -4.78910600    1.04827900 H                 -2.86795100   -1.51973400    0.98338900 H                  2.86723900   -1.52281700    0.97722700 C                  5.55532400   -1.30305300    0.38728100 H                  5.17542900   -2.22164800    0.84151300 H                  6.06903800   -1.56777000   -0.54304900 H                  6.30599800   -0.87477100    1.06061700 C                 -5.55639700   -1.29851100    0.39575200 H                 -6.07108400   -1.56320800   -0.53405700 H                 -5.17659700   -2.21718900    0.84988800 H                 -6.30625600   -0.86962900    1.069613005-3MCRu                 0.00394000    0.45985000    0.25612700 O                 -0.22041400    2.45268000    0.67871500 O                 -0.02294100    0.84975200   -1.75086100 N                  2.29371600   -0.21566400    0.26475200 N                  0.10247900   -1.66292900   -0.28105200 N                 -2.20567500   -0.41788800    0.30642300 C                 -3.29966000    0.33177200    0.48921200 C                 -4.58185800   -0.07404000    0.10835900 C                 -3.55430200   -2.11540500   -0.69489900 H                 -3.63986500   -3.07654600   -1.18727200 C                 -4.68656400   -1.33147200   -0.50349600 H                 -5.65541100   -1.69282300   -0.83552800 C                 -2.31104500   -1.63161800   -0.26703800 C                 -1.04187600   -2.37879800   -0.41807900 C                 -1.01077900   -3.75208000   -0.68095600 H                 -1.93157100   -4.31622600   -0.75625600 C                  0.21636700   -4.39208400   -0.81682000 H                  0.26103700   -5.45919200   -1.00491000 C                  1.38556400   -3.64897700   -0.69649000 H                  2.35035000   -4.13173400   -0.78480500 C                  1.30149800   -2.27792700   -0.43259400 C                  2.50191800   -1.42288900   -0.29428800 C                  3.78141600   -1.80518100   -0.71780100 H                  3.94731900   -2.76090700   -1.20003900 C                  4.84395600   -0.92731000   -0.53543200 H                  5.83962200   -1.20945200   -0.86481600 C                  4.63394100    0.32256000    0.06413600 C                  3.32138900    0.62404000    0.43964000 C                 -0.13383800    2.00586100   -3.79552600 H                 -0.24910600    3.00497300   -4.21849800 H                  0.80999800    1.57508900   -4.14603800 C                 -0.15016500    2.01006100   -2.28552700 C                 -0.29766600    3.22569300   -1.59206700 H                 -0.39920700    4.12065000   -2.19332200 C                 -0.32792700    3.39144600   -0.20115400 C                 -0.50092800    4.77039300    0.38820400 H                 -0.58663000    5.54526400   -0.37514100 H                  0.35230300    4.99718000    1.03613600 N                  0.04432200    0.13528500    2.28950400 C                  0.06298300   -0.04351500    3.43400400 C                  0.08553200   -0.27283000    4.87176200 H                  1.02920000    0.08704400    5.29065500 H                 -0.74255900    0.26079300    5.34600500 H                 -0.01311800   -1.34202200    5.07825200 H                 -0.94062500    1.36417800   -4.16479000 H                 -1.39768100    4.78483700    1.01666700 H                  3.07848700    1.57950200    0.89825100 H                 -3.13554000    1.29927500    0.95749600 C                 -5.78554100    0.79770300    0.34545600 H                 -6.48781700    0.31152600    1.03136400 H                 -5.50322500    1.76212300    0.77490300 H                 -6.32234600    0.98249600   -0.59082000 C                  5.76141300    1.29101800    0.30057200 H                  5.39140300    2.26121000    0.64127700 H                  6.44955200    0.90484800    1.06077900 H                  6.34203900    1.44493800   -0.614407005-TSIIfreq = -97.02 cm-1Ru                 0.05514000    0.39149500    0.05580400 O                  0.00726900    2.39710900    0.50508800 O                 -0.68410200    0.81063100   -1.86467000 N                  2.23577300   -0.08968800    0.04080000 N                  0.14709400   -1.60149300   -0.43917000 N                 -2.02827400   -0.47429400    0.56293100 C                 -3.08342700    0.23116600    0.99454700 C                 -4.40742400   -0.17261000    0.80459600 C                 -3.51934800   -2.11819400   -0.33445300 H                 -3.67888000   -3.04076300   -0.87982000 C                 -4.60690100   -1.37849200    0.11601600 H                 -5.61581400   -1.73388200   -0.07110600 C                 -2.22543000   -1.64340500   -0.09244500 C                 -0.99429800   -2.34107700   -0.50798100 C                 -0.95410300   -3.67840800   -0.90780200 H                 -1.86492600   -4.26251600   -0.94752200 C                  0.27025300   -4.26164000   -1.22443100 H                  0.31803800   -5.30086600   -1.52984600 C                  1.43568200   -3.50762800   -1.11727400 H                  2.39801000   -3.95681600   -1.32798400 C                  1.35342000   -2.17261100   -0.71537400 C                  2.51672500   -1.29088800   -0.52490900 C                  3.83165900   -1.62251100   -0.87069000 H                  4.05778900   -2.57294400   -1.33872600 C                  4.85070300   -0.71313100   -0.61607000 H                  5.87382900   -0.96080300   -0.88239500 C                  4.56424400    0.52388500   -0.01745300 C                  3.22717100    0.78146700    0.28797300 C                 -1.56457900    1.99785400   -3.69709800 H                 -1.81147900    3.00408500   -4.03957700 H                 -0.83757400    1.55238600   -4.38411900 C                 -1.00235100    1.98050500   -2.29438500 C                 -0.86782600    3.18627000   -1.58630900 H                 -1.16967700    4.08977300   -2.10152100 C                 -0.37821900    3.34156800   -0.27749200 C                 -0.27768400    4.72416000    0.32262300 H                 -0.61487400    5.50430400   -0.36151000 H                  0.76195200    4.92011400    0.60537100 N                  0.44260400    0.10045100    2.43073400 C                  0.67976500   -0.20629600    3.52278700 C                  0.98127400   -0.59128600    4.89561500 H                  2.03287200   -0.38758200    5.11356300 H                  0.35474100   -0.02192300    5.58687600 H                  0.78695100   -1.65831200    5.03142200 H                 -2.46560400    1.37633700   -3.73097200 H                 -0.87631400    4.76606200    1.23857600 H                  2.92420000    1.71824400    0.74669200 H                 -2.84956000    1.15885800    1.50981100 C                 -5.55995300    0.64813300    1.31785300 H                 -6.13061300    0.08959400    2.06772300 H                 -5.21666500    1.57917200    1.77550900 H                 -6.25018900    0.89888600    0.50570900 C                  5.64478900    1.52653800    0.28575500 H                  5.23321100    2.44051300    0.72075200 H                  6.37288700    1.11026600    0.99017300 H                  6.19095600    1.79506100   -0.624515005-3[RuPenta:ACN]Ru                -0.05223300    0.39301300    0.08003900 O                 -0.05872200    2.27299900    0.91642700 O                  0.06424500    1.31361800   -1.88268100 N                  1.98901600   -0.15602900    0.23390800 N                 -0.04376500   -1.45721400   -0.66444300 N                 -2.07986200   -0.16655300    0.23281900 C                 -3.06235500    0.59513300    0.75109600 C                 -4.41281800    0.26329400    0.64944400 C                 -3.72280800   -1.71824000   -0.56532500 H                 -3.97001900   -2.63206200   -1.09195100 C                 -4.72931600   -0.92315100   -0.03101000 H                 -5.76713500   -1.22212900   -0.13997100 C                 -2.39045900   -1.32618300   -0.42206600 C                 -1.22895500   -2.08648300   -0.89790400 C                 -1.24440900   -3.35480000   -1.47988000 H                 -2.18290600   -3.86300500   -1.66377200 C                 -0.03353900   -3.96936100   -1.80173900 H                 -0.02957300   -4.95437600   -2.25406200 C                  1.17253500   -3.33164500   -1.50865900 H                  2.11587300   -3.82247000   -1.71372000 C                  1.14653000   -2.06628300   -0.92152800 C                  2.30473700   -1.29753700   -0.44879700 C                  3.63971200   -1.67239500   -0.61401900 H                  3.89149800   -2.57252500   -1.16166500 C                  4.64317100   -0.87731600   -0.07340300 H                  5.68296600   -1.16281700   -0.19920800 C                  4.32136200    0.29092400    0.63485000 C                  2.96791700    0.60567400    0.75648700 C                  0.18800300    2.98816600   -3.53755500 H                  0.22329300    4.07052500   -3.67290300 H                  1.08250500    2.54060800   -3.98321500 C                  0.10106200    2.57930400   -2.08318300 C                  0.06743800    3.58100200   -1.09280900 H                  0.10523200    4.60564200   -1.44154200 C                 -0.00919000    3.40005900    0.29806600 C                 -0.03745400    4.60611300    1.20708800 H                  0.00682900    5.54816000    0.65862400 H                  0.80784400    4.55390100    1.90125500 N                 -0.01386600   -0.77905700    3.56537500 C                  0.20799300   -1.78796100    3.03446600 C                  0.48519900   -3.05088700    2.35809800 H                  1.46650800   -3.00813400    1.87887800 H                  0.47815700   -3.87293000    3.07800000 H                 -0.27662500   -3.23934600    1.59751100 H                 -0.67735600    2.58681800   -4.07544800 H                 -0.95244300    4.58274200    1.80825600 H                  2.63740800    1.49555500    1.28228400 H                 -2.73604400    1.49919500    1.25468300 C                 -5.47902700    1.14169900    1.24584000 H                 -6.05415800    0.59280500    1.99896400 H                 -5.05064700    2.02743800    1.72061200 H                 -6.18318400    1.47121300    0.47482300 C                  5.38394300    1.16700200    1.24084000 H                  4.95228700    2.04796600    1.72141500 H                  5.95752200    0.61338400    1.99174800 H                  6.08993800    1.50353200    0.47472400
